# Mathematical Derivation of Wave Propagation Properties in Hierarchical Neural Networks with Predictive Coding Feedback Dynamics

**DOI:** 10.1007/s11538-023-01186-9

**Published:** 2023-07-28

**Authors:** Grégory Faye, Guilhem Fouilhé, Rufin VanRullen

**Affiliations:** 1 Institut de Mathématiques de Toulouse, UMR5219, UPS IMT, Université de Toulouse, 31062 Toulouse Cedex 9, France; 2 Centre de Recherche Cerveau et Cognition (CerCo), UMR5549, Université de Toulouse, 31052 Toulouse, France; 3 ANITI, Université de Toulouse, 31062 Toulouse, France

**Keywords:** Hierarchical neural networks, Predictive coding, Wave propagation

## Abstract

Sensory perception (e.g., vision) relies on a hierarchy of cortical areas, in which neural activity propagates in both directions, to convey information not only about sensory inputs but also about cognitive states, expectations and predictions. At the macroscopic scale, neurophysiological experiments have described the corresponding neural signals as both forward and backward-travelling waves, sometimes with characteristic oscillatory signatures. It remains unclear, however, how such activity patterns relate to specific functional properties of the perceptual apparatus. Here, we present a mathematical framework, inspired by neural network models of predictive coding, to systematically investigate neural dynamics in a hierarchical perceptual system. We show that stability of the system can be systematically derived from the values of hyper-parameters controlling the different signals (related to bottom-up inputs, top-down prediction and error correction). Similarly, it is possible to determine in which direction, and at what speed neural activity propagates in the system. Different neural assemblies (reflecting distinct eigenvectors of the connectivity matrices) can simultaneously and independently display different properties in terms of stability, propagation speed or direction. We also derive continuous-limit versions of the system, both in time and in neural space. Finally, we analyze the possible influence of transmission delays between layers, and reveal the emergence of oscillations.

## Introduction

The brain’s anatomy is characterized by a strongly hierarchical architecture, with a succession of brain regions that process increasingly complex information. This functional strategy is mirrored by the succession of processing layers found in modern deep neural networks (and for this reason, we use the term “layer” in this work to denote one particular brain region in this hierarchy, rather than the laminar organization of cortex that is well-known to neuroscientists). The hierarchical structure is especially obvious in the organization of the visual system (Felleman and Essen 1991), starting from the retina through primary visual cortex (V1) and various extra-striate regions, and culminating in temporal lobe regions for object recognition and in parietal regions for motion and location processing.

In this hierarchy of brain regions, the flow of information is clearly bidirectional: there are comparable number of fibers sending neural signals down (from higher to lower levels of the hierachy) as there are going up (Bullier 2001). While the bottom-up or “feed-forward” propagation of information is easily understood as integration of sensory input (and matches the functional structure found in artificial deep learning networks), the opposite feedback direction of propagation is more mysterious, and its functional role remains unknown.

Predictive coding is one dominant theory to explain the function of cortical feedback (Rao and Ballard 1999). Briefly, the theory states that each layer in the cortical hierarchy generates predictions about what caused their own activity; these predictions are sent to the immediately preceding layer, where a prediction error can be computed, and carried forward to the original layer, which can then iteratively update its prediction. Over time (and as long as the sensory input does not change), the system settles into a state where top-down predictions agree with bottom-up inputs, and no prediction error is transmitted. Like any large-scale theory of brain function, the predictive coding theory is heavily debated (Millidge et al. 2021). But macroscopic (EEG) experiments have revealed characteristic propagation signatures that could be hallmarks of predictive coding. For instance, Alamia and VanRullen (2019) showed evidence for alpha-band (7–15 Hz) oscillatory travelling waves propagating in both directions (feed-forward and feedback); the oscillation frequency and dynamics were compatible with a simplistic hierarchical model that included a biologically plausible time delay for transmitting signals between layers, and was also confirmed by a rudimentary mathematical model. In another study, Bastos et al. (2012, 2015) found that beta (15–30 Hz) and gamma-frequency (30–100 Hz) oscillations could reflect, respectively, the predictions and prediction errors signals carried by backward and forward connections.

More recently, predictive coding has been explored in the context of deep neural networks (Wen et al. 2018; Choksi et al. 2021; Pang et al. 2021). For instance, Choksi et al. (2021) augmented existing deep convolutional networks with feedback connections and a mechanism for computing and minimizing prediction errors, and found that the augmented system displayed more robust perception, better aligned with human abilities. In another study, Pang et al. (2021) used a similar system and reported the emergence of illusory contour perception comparable to what humans (but not standard deep neural networks) would typically perceive.

While the concept of predictive coding is potentially fundamental for understanding brain function, and its large-scale implementation in deep artificial neural networks provides empirical support for its potential functional relevance, there is a gap of theoretical knowledge about the type of brain activity that predictive coding could engender, and the potential conditions for its stability. Here, we propose a mathematical framework where a potentially infinite number of neuronal layers exchange signals in both directions according to predictive coding principles. The stable propagation of information in such a system can be explored analytically as a function of its initial state, its internal parameters (controlling the strength of inputs, predictions, and error signals) and its connectivity (e.g., convolution kernels). Our approach considers both a discrete approximation of the system, as well as continuous abstractions. We demonstrate the practical relevance of our findings by applying them to a ring model of orientation processing. Finally, we extend our analytical framework to the case where communication delays between successive layers are included. This gives rise to oscillatory signals at frequencies consistent with those observed in the brain.

## Model Description

Our initial model is inspired by the generic formulation of predictive coding proposed in the context of deep learning models by Choksi et al. (2021). This formulation considers different update terms at each time step: feed-forward inputs, memory term, feedback- and feed-forward prediction error corrections. By modulating the hyper-parameters controlling each of these terms, the model can be reconciled with different formulations of predictive coding (for instance, the Rao and Ballard Rao and Ballard (1999) model by setting the feed-forward input term to zero) or other models of hierarchical brain function (e.g., similar to Heeger’s model (Heeger 2017) by setting the feed-forward error correction to zero). Indeed, our objective is precisely to characterize the propagation dynamics inside the network as a function of the relative value of these hyper-parameters, which in turn alters the model’s functionality.

We consider the following recurrence equation where ${\mathcal {E}}_j^n\in {\mathbb {R}}^d$ represents an encoder at step *n* and layer *j*

$$\begin{aligned} {\mathcal {E}}_j^{n+1}=\beta {\mathcal {W}}^f {\mathcal {E}}_{j-1}^{n+1} +(1-\beta ) {\mathcal {E}}_j^{n}-\alpha {\mathcal {F}}_{j-1}^n -\lambda {\mathcal {B}}_{j}^n, \quad j=1,\ldots ,J-1 , \end{aligned}$$

where ${\mathcal {W}}^f\in {\mathscr {M}}_d({\mathbb {R}})$ is a $d\times d$ square matrix representing the weights of feedforward connections which we assume to be the same for each layer such that ${\mathcal {W}}^f {\mathcal {E}}_{j-1}^{n+1}$ models an instantaneous feedforward drive from layer $j-1$ to layer *j*, controlled by hyper-parameter $\beta$. The term ${\mathcal {F}}_{j-1}^n$ encodes a feedforward error correction process, controlled by hyper-parameter $\alpha$, where the reconstruction error ${\mathcal {R}}_{j-1}^n$ at layer $j-1$, defined as the square error between the representation ${\mathcal {E}}_{j-1}^n$ and the predicted reconstruction ${\mathcal {W}}^b{\mathcal {E}}_j^n$, that is

$$\begin{aligned} {\mathcal {R}}_{j-1}^n:= \frac{1}{2} \Vert {\mathcal {E}}_{j-1}^n-{\mathcal {W}}^b{\mathcal {E}}_j^n\Vert ^2, \end{aligned}$$

propagates to the layer *j* to update its representation. Here, ${\mathcal {W}}^b\in {\mathscr {M}}_d({\mathbb {R}})$ is a $d\times d$ square matrix representing the weights of feedback connections which we assume to be the same for each layer. Following (Rao and Ballard 1999; Choksi et al. 2021; Wen et al. 2018; Alamia and VanRullen 2019), the contribution ${\mathcal {F}}_{j-1}^n$ is then taken to be the gradient of ${\mathcal {R}}_{j-1}^n$ with respect to ${\mathcal {E}}_j^n$, that is

$$\begin{aligned} {\mathcal {F}}_{j-1}^n = \nabla {\mathcal {R}}_{j-1}^n = - ({\mathcal {W}}^b) ^{\textbf{t}}{\mathcal {E}}_{j-1}^n + ({\mathcal {W}}^b )^{\textbf{t}} {\mathcal {W}}^b {\mathcal {E}}_{j}^n. \end{aligned}$$

On the other hand, ${\mathcal {B}}_{j}^n$ incorporates a top-down prediction to update the representation at layer *j*. This term thus reflects a feedback error correction process, controlled by hyper-parameter $\lambda$. Similar to the feedforward process, ${\mathcal {B}}_{j}^n$ is defined as the the gradient of ${\mathcal {R}}_{j}^n$ with respect to ${\mathcal {E}}_j^n$, that is

$$\begin{aligned} {\mathcal {B}}_{j}^n = \nabla {\mathcal {R}}_{j}^n = -{\mathcal {W}}^b {\mathcal {E}}_{j+1}^n+{\mathcal {E}}_j^n. \end{aligned}$$

As a consequence, our model reads

1 $$\begin{aligned} {\mathcal {E}}_j^{n+1}=\beta {\mathcal {W}}^f {\mathcal {E}}_{j-1}^{n+1} +\alpha ({\mathcal {W}}^b) ^{\textbf{t}}{\mathcal {E}}_{j-1}^n+\left[ (1-\beta -\lambda ){\textbf{I}}_d-\alpha ({\mathcal {W}}^b )^{\textbf{t}} {\mathcal {W}}^b \right] {\mathcal {E}}_j^{n} + \lambda {\mathcal {W}}^b {\mathcal {E}}_{j+1}^{n},\nonumber \\ \end{aligned}$$

for each $j=1,\ldots ,J-1$ and $n\ge 0$ where we denoted ${\textbf{I}}_d$ the identity matrix of ${\mathscr {M}}_d({\mathbb {R}})$. We supplement the recurrence Eq. (1) with the following boundary conditions at layer $j=0$ and layer $j=J$. First, at layer $j=0$, we impose

2 $$\begin{aligned} {\mathcal {E}}_0^n={\mathcal {S}}_0^n, \quad n\ge 0, \end{aligned}$$

where ${\mathcal {S}}_0^n \in {\mathbb {R}}^d$ is a given source term, which can be understood as the network’s constant visual input. At the final layer $j=J$, there is no possibility of incoming top-down signal, and thus one gets

3 $$\begin{aligned} {\mathcal {E}}_J^{n+1}=\beta {\mathcal {W}}^f {\mathcal {E}}_{J-1}^{n+1} +\alpha ({\mathcal {W}}^b) ^{\textbf{t}}{\mathcal {E}}_{J-1}^n+\left[ (1-\beta ){\textbf{I}}_d-\alpha ({\mathcal {W}}^b )^{\textbf{t}} {\mathcal {W}}^b \right] {\mathcal {E}}_J^{n}, \quad n\ge 0.\nonumber \\ \end{aligned}$$

Finally, at the initial step $n=0$, we set

4 $$\begin{aligned} {\mathcal {E}}^0_j={\mathcal {H}}_j, \quad j=0,\ldots ,J, \end{aligned}$$

for some given initial sequence $({\mathcal {H}}_j)_{0,\ldots ,J}$. For instance, in Choksi et al. (2021), ${\mathcal {H}}_j$ was initialized by a first feedforward pass through the system, i.e., $\beta >0$ and $\alpha =\lambda =0$. Throughout we assume the natural following compatibility condition between the source terms and the initial condition, namely

5 $$\begin{aligned} {\mathcal {S}}_0^0={\mathcal {H}}_0. \end{aligned}$$

Regarding the hyper-parameters of the problem we assume that

6 $$\begin{aligned} 0\le \beta <1, \quad \text { with } \quad 0 \le \alpha +\lambda \le 1. \end{aligned}$$

Our key objective is to characterize the behavior of the solutions of the above recurrence Eq. (1) as a function of the hyper-parameters and the feedforward and feedback connections matrices ${\mathcal {W}}^f$ and ${\mathcal {W}}^b$. We would like to stay as general as possible to encompass as many situations as possible, keeping in mind that we already made strong assumptions by imposing that the weight matrices of feedforward and feedback connections are identical from one layer to another and that we only consider a linear model although deep neural networks are intrinsically nonlinear. Motivated by concrete applications, we will mainly consider matrices ${\mathcal {W}}^f$ and ${\mathcal {W}}^b$ which act as convolutions on ${\mathbb {R}}^d$.

## The Identity Case

It turns out that we will gain much information by first treating the simplified case where ${\mathcal {W}}^f$ and ${\mathcal {W}}^b$ are both identity. That is, from now on, and throughout this section we assume that

$$\begin{aligned} {\mathcal {W}}^f={\mathcal {W}}^b={\textbf{I}}_d. \end{aligned}$$

**Fig. 1:**
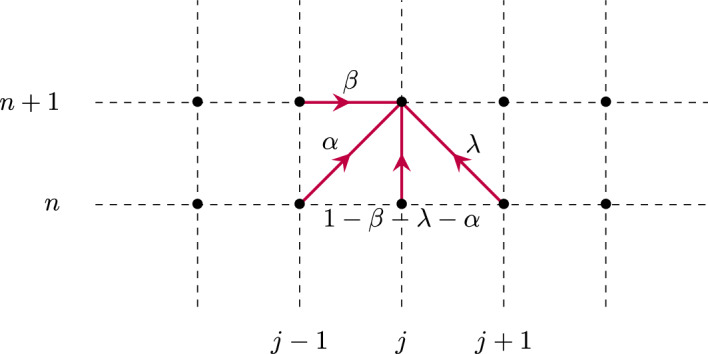
Schematic illustration of the network structure of model (7) where each point represents a given neuronal layer index *j* (x-axis) at a particular time step *n* (y-axis), and the red arrows indicate the contributions leading to the update of $e_j^{n+1}$ (Color figure online)

That is, each neuron in a layer is only connected to the corresponding neuron in the immediately preceding and following layer, with unit weight in each direction. Under such a setting, the recurrence Eq. (1) reduces to a scalar equation (Fig. 1), that is

7 $$\begin{aligned} e_j^{n+1}=\beta e_{j-1}^{n+1} +\alpha e_{j-1}^n+(1-\beta -\lambda - \alpha )e_j^{n} + \lambda e_{j+1}^{n}, \quad j=1,\ldots ,J-1, \end{aligned}$$

with this time the unknown $e_j^n\in {\mathbb {R}}$, together with

8 $$\begin{aligned} e_0^n=s_0^n, \quad n\ge 0, \end{aligned}$$

and

9 $$\begin{aligned} e_J^{n+1}=\beta e_{J-1}^{n+1} +\alpha e_{J-1}^n+(1-\beta -\alpha )e_J^{n}, \quad n\ge 0. \end{aligned}$$

with

10 $$\begin{aligned} e^0_j=h_j, \quad j=0,\ldots ,J. \end{aligned}$$

### Wave Propagation on an Infinite Depth Network

It will be first useful to consider the above problem set on an infinite domain and look at

11 $$\begin{aligned} e_j^{n+1}=\beta e_{j-1}^{n+1} +\alpha e_{j-1}^n+(1-\beta -\lambda - \alpha )e_j^{n} + \lambda e_{j+1}^{n}, \quad j\in {\mathbb {Z}}, \end{aligned}$$

given some initial sequence

$$\begin{aligned} e^0_j=h_j, \quad j\in {\mathbb {Z}}. \end{aligned}$$

This situation has no direct equivalent in practical deep neural networks (nor in the brain), where the number of hierarchically connected layers is necessarily finite; but it is a useful mathematical construct. Indeed, such recurrence equations set on the integers ${\mathbb {Z}}$ are relatively well understood from the mathematical numerical analysis community. The behavior of the solution sequence $(e_j^n)_{j\in {\mathbb {Z}}}$ can be read out from the so-called amplification factor function defined as

12 $$\begin{aligned} \rho (\theta ):=\frac{\alpha \left( e^{-{\textbf{i}}\theta }-1\right) +1-\beta +\lambda \left( e^{{\textbf{i}}\theta }-1\right) }{1-\beta e^{-{\textbf{i}}\theta }}, \quad \theta \in [-\pi ,\pi ], \end{aligned}$$

and which relates spatial and temporal modes. Indeed, formally, the sequence $(\rho (\theta )^n e^{{\textbf{i}}j\theta })_{j\in {\mathbb {Z}}}$ is an explicit solution to (11) for each $\theta \in [-\pi ,\pi ]$. Actually one can be much more precise and almost explicit in the sense that one can relate the expression of the solutions to (11) starting from some initial sequence $(h_j)_{j\in {\mathbb {Z}}}$ to the properties of $\rho$ in a systematic way that we now briefly explain.

Let us first denote by ${\mathcal {G}}^n=({\mathcal {G}}^n_j)_{j\in {\mathbb {Z}}}$ the sequence which is the fundamental solution of (11) in the special case where $({\mathcal {H}}_j)_{j\in {\mathbb {Z}}}$ is the Dirac delta sequence $\varvec{\delta }$. The Dirac delta sequence $\varvec{\delta }$ is defined as $\varvec{\delta }_0=1$ and $\varvec{\delta }_j=0$ for all $j\in {\mathbb {Z}}\backslash \{0\}$. As a consequence, we have ${\mathcal {G}}^0=\varvec{\delta }$ and for each $n\ge 0$

$$\begin{aligned} {\mathcal {G}}_j^{n+1}-\beta {\mathcal {G}}_{j-1}^{n+1}= \alpha {\mathcal {G}}_{j-1}^{n} +(1-\beta -\lambda -\alpha ){\mathcal {G}}_j^{n}+\lambda {\mathcal {G}}_{j+1}^{n}, \quad j\in {\mathbb {Z}}. \end{aligned}$$

The starting point of the analysis is the following representation formula, obtained via inverse Fourier transform, which reads

13 $$\begin{aligned} {\mathcal {G}}^n_j=\frac{1}{2\pi } \int _{-\pi }^\pi e^{{\textbf{i}}j \theta } \rho (\theta )^n \textrm{d}\theta , \quad n\ge 1, \quad j\in {\mathbb {Z}}. \end{aligned}$$

Then, given any initial sequence $(h_j)_{j\in {\mathbb {Z}}}$, the solution $(e_j^n)_{j\in {\mathbb {Z}}}$ to (11) can be represented as the convolution product between the initial sequence and the fundamental solution, namely

14 $$\begin{aligned} e_j^n=\sum _{\ell \in {\mathbb {Z}}} {\mathcal {G}}_{j-\ell }^nh_\ell , \quad j\in {\mathbb {Z}}, \quad n\ge 1. \end{aligned}$$

That is, having characterized the fundamental solution for a simple input pattern ($\varvec{\delta }$), with a unitary impulse provided to a single layer, we can now easily generalize to any arbitrary input pattern, by applying the (translated) fundamental solution to each layer.

Our aim is to understand under which conditions on the hyper-parameters we can ensure that the solutions of (11) given through (14) remain bounded for all $n\ge 1$ independently of the choice of the initial sequence $(h_j)_{j\in {\mathbb {Z}}}$. More precisely, we introduce the following terminology. We say that the recurrence equation is *stable* if for each bounded initial sequence $(h_j)_{j\in {\mathbb {Z}}}\in \ell ^{\infty }({\mathbb {Z}})$, the corresponding solution $(e_j^n)_{j\in {\mathbb {Z}}}$ given by (14) satisfies

$$\begin{aligned} \underset{j\in {\mathbb {Z}}}{\sup }|e_j^n|\underset{n\rightarrow \infty }{\longrightarrow }0. \end{aligned}$$

On the other hand, we say that the recurrence equation is *unstable* if one can find a bounded initial sequence $(h_j)_{j\in {\mathbb {Z}}}\in \ell ^{\infty }({\mathbb {Z}})$ such that the corresponding solution $(e_j^n)_{j\in {\mathbb {Z}}}$ given by (14) satisfies

$$\begin{aligned} \underset{j\in {\mathbb {Z}}}{\sup }|e_j^n|\underset{n\rightarrow \infty }{\longrightarrow }+\infty . \end{aligned}$$

Finally, we say that the recurrence equation is *marginally stable* if there exists a universal constant $C>0$ such that for each bounded initial sequence $(h_j)_{j\in {\mathbb {Z}}}\in \ell ^{\infty }({\mathbb {Z}})$, the corresponding solution $(e_j^n)_{j\in {\mathbb {Z}}}$ given by (14) satisfies

$$\begin{aligned} \underset{j\in {\mathbb {Z}}}{\sup }|e_j^n|\le C ~ \underset{j\in {\mathbb {Z}}}{\sup }|h_j|, \quad n\ge 1. \end{aligned}$$

It turns out that one can determine the stability properties of the recurrence equation by solely looking at the amplification factor function. Indeed, from Riesz and Nagy (1955), we know that

$$\begin{aligned} \underset{n\rightarrow \infty }{\lim }\ \Vert {\mathcal {G}}^n \Vert _{\ell ^1({\mathbb {Z}})}^{1/n} = \underset{\theta \in [-\pi ,\pi ]}{\max }|\rho (\theta )|, \end{aligned}$$

where we have set

$$\begin{aligned} \Vert {\mathcal {G}}^n \Vert _{\ell ^1({\mathbb {Z}})}:=\sum _{j\in {\mathbb {Z}}}|{\mathcal {G}}_j^n|. \end{aligned}$$

As a consequence, we directly deduce that the recurrence equation is stable when $|\rho (\theta )|<1$ for all $\theta \in [-\pi ,\pi ]$, whereas it is unstable if there exists $\theta _0\in [-\pi ,\pi ]$ such that $|\rho (\theta _0)|> 1$. The limiting case occurs precisely when $\underset{\theta \in [-\pi ,\pi ]}{\max }|\rho (\theta )|=1$ and there is actually a long history of works (Thomée 1965; Diaconis and Saloff-Coste 2014; Randles and Saloff-Coste 2015; Coulombel and Faye 2022; Coeuret 2022) that have studied the marginal stability of the recurrence equation in that case. All such results rely on a very precise understanding of the amplification factor function and lead to the following statement.

#### Theorem 1

(Thomée 1965; Diaconis and Saloff-Coste 2014; Randles and Saloff-Coste 2015; Coulombel and Faye 2022; Coeuret 2022) Suppose that there exist finitely many $\theta _1,\ldots ,\theta _K\in [-\pi ,\pi ]$ such that for all $\theta \in [-\pi ,\pi ]\backslash \left\{ \theta _1,\ldots ,\theta _k\right\}$ one has $|\rho (\theta )|<1$ and $|\rho (\theta _k)|=1$ for each $k=1,\ldots ,K$. Furthermore, assume that there exist $c_k\in {\mathbb {R}}$, $\sigma _k\in {\mathbb {C}}$ with $\textrm{Re}(\sigma _k)>0$ and an integer $\mu _k\ge 1$ such that

$$\begin{aligned} \frac{\rho (\theta _k+\theta )}{\rho (\theta _k)}=\exp \left( -{\textbf{i}}c_k\theta -\sigma _k \theta ^{2\mu _k}+{\mathcal {O}}(|\theta |^{2\mu _k+1})\right) , \text { as } \theta \rightarrow 0. \end{aligned}$$

Then the recurrence equation is marginally stable.

Based on the above notions of stability/instability, we see that the only interesting situation is when the recurrence equation is marginally stable, and thus when the amplification function is contained in the unit disk with finitely many tangent points to the unit circle with prescribed asymptotic expansions. This is also the only interesting situation from a biological standpoint, as it ensures that the network remains active, yet without runaway activations.

#### Study of the Amplification Factor Function

Since we assumed that $0\le \beta <1$, the denominator in (12) never vanishes and is well-defined. Next, we crucially remark that we always have

$$\begin{aligned} \rho (0)=1. \end{aligned}$$

We will now check under which conditions $|\rho (\theta )|\le 1$ for all $\theta \in [-\pi ,\pi ]$ to guarantee marginal stability of the recurrence equation.

To assess stability, we compute

$$\begin{aligned} \left| \rho (\theta )\right| ^2&=\frac{\left( (\lambda +\alpha )(\cos (\theta )-1)+1-\beta \right) ^2+(\lambda -\alpha )^2\sin (\theta )^2}{1-2\beta \cos (\theta )+\beta ^2}\\&=\frac{(\lambda +\alpha )^2(\cos (\theta )-1)^2+2(1-\beta )(\lambda +\alpha )(\cos (\theta )-1)+\left( 1-\beta \right) ^2+(\lambda -\alpha )^2(1-\cos (\theta )^2)}{(1-\beta )^2+2\beta (1-\cos (\theta ))}\\&=\frac{(1-\cos (\theta ))\left( (\lambda +\alpha )^2(1-\cos (\theta ))-2(1-\beta )(\lambda +\alpha )+(\lambda -\alpha )^2(1+\cos (\theta ))\right) +\left( 1-\beta \right) ^2}{(1-\beta )^2+2\beta (1-\cos (\theta ))}\\&=\frac{(1-\cos (\theta ))\left( -4\alpha \lambda \cos (\theta )-2(1-\beta )(\lambda +\alpha )+2(\lambda ^2+\alpha ^2)\right) +\left( 1-\beta \right) ^2}{(1-\beta )^2+2\beta (1-\cos (\theta ))} \end{aligned}$$

such that $\left| \rho (\theta )\right| ^2\le 1$ is equivalent to

$$\begin{aligned} (1-\cos (\theta ))\left( 2\beta +4\alpha \lambda \cos (\theta ) +2(1-\beta )(\lambda +\alpha )-2(\lambda ^2+\alpha ^2)\right) \ge 0, \quad \theta \in [-\pi ,\pi ], \end{aligned}$$

and since $1-\cos (\theta )\ge 0$ we need to ensure

$$\begin{aligned} \beta +2\alpha \lambda \cos (\theta ) +(1-\beta )(\lambda +\alpha )-\lambda ^2-\alpha ^2\ge 0, \quad \theta \in [-\pi ,\pi ], \end{aligned}$$

and evaluating at $\pm \pi$ the above inequality we get

$$\begin{aligned} \beta +(1-\beta )(\lambda +\alpha )-(\lambda +\alpha )^2\ge 0. \end{aligned}$$

But we remark that the above expression can be factored as

$$\begin{aligned} \left( \beta +\lambda +\alpha \right) \left( 1-\lambda -\alpha \right) \ge 0. \end{aligned}$$

As a consequence, $\left| \rho (\theta )\right| ^2\le 1$ if and only if $\lambda +\alpha \le 1$. This is precisely the condition that we made in (6). We can actually track cases of equality which are those values of $\theta \in [-\pi ,\pi ]$ for which we have

$$\begin{aligned} (1-\cos (\theta ))\left( 2\beta +4\alpha \lambda \cos (\theta ) +2(1-\beta )(\lambda +\alpha )-2(\lambda ^2+\alpha ^2)\right) = 0. \end{aligned}$$

We readily recover that at $\theta =0$ we have $|\rho (0)|=1$. So, now assuming that $\theta \ne 0$, we need to solve

$$\begin{aligned} \beta +2\alpha \lambda \cos (\theta ) +(1-\beta )(\lambda +\alpha )-\lambda ^2-\alpha ^2 = 0, \end{aligned}$$

which we write as

$$\begin{aligned} \beta -2\alpha \lambda +(1-\beta )(\lambda +\alpha )-\lambda ^2-\alpha ^2 +2\alpha \lambda \left( \cos (\theta )+1)\right) = 0, \end{aligned}$$

and using the previous factorization we get

$$\begin{aligned} \left( \beta +\lambda +\alpha \right) \left( 1-\lambda -\alpha \right) +2\alpha \lambda \left( \cos (\theta )+1)\right) = 0, \end{aligned}$$

and we necessarily get that both $1+\cos (\theta )=0$ and $1-\lambda -\alpha =0$ must be satisfied. As consequence, $|\rho (\pm \pi )|=1$ if and only if $1=\lambda +\alpha$.

As a summary we have obtained that:

- if $0\le \lambda +\alpha <1$ and $0\le \beta <1$, then $|\rho (\theta )|<1$ for all $\theta \in [-\pi ,\pi ]\backslash \{0\}$ with $\rho (0)=1$;
- if $\lambda +\alpha =1$ and $0\le \beta <1$, then $|\rho (\theta )|<1$ for all $\theta \in (-\pi ,\pi )\backslash \{0\}$ with $\rho (0)=1$ and $\rho (\pm \pi )=-1$.

**Fig. 2:**
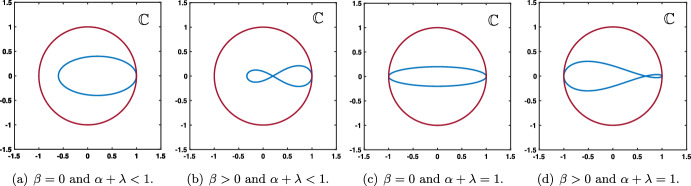
Several representative illustration of the curve $\theta \mapsto \rho (\theta )$ for $\theta \in [-\pi ,\pi ]$ in the case where $\beta =0$ and $\beta \ne 0$. **a** and **b** Amplification factor function $\rho (\theta )$ (blue curve) with a unique tangency point on the unit circle at $z=1$ corresponding $\theta =0$. **c** and **d** When $\alpha +\lambda =1$ the function $\rho (\theta )$ (blue curve) has two tangency points on the unit circle at $z=1$ corresponding $\theta =0$ and $z=-1$ corresponding to $\theta =\pm \pi$ (Color figure online)

We present in Fig. 2 several representative illustrations of the spectral curves $\rho (\theta )$ for various values of the hyper-parameters recovering the results explained above.

Furthermore, near $\theta \sim 0$, we get that $\rho$ admits the following asymptotic expansion

$$\begin{aligned}{} & {} \rho (\theta ) = \exp \left( - {\textbf{i}}\frac{\beta +\alpha -\lambda }{1-\beta } \theta -\frac{\beta (1-\alpha -\lambda )+\alpha +\lambda -(\lambda -\alpha )^2}{2(1-\beta )^2}\theta ^2+{\mathcal {O}}(|\theta |^3)\right) , \\{} & {} \quad \text { as } \theta \rightarrow 0, \end{aligned}$$

provided that

$$\begin{aligned} \beta (1-\alpha -\lambda )+\alpha +\lambda -(\lambda -\alpha )^2 \ne 0. \end{aligned}$$

In fact, since $-(\lambda -\alpha )^2\ge -(\lambda +\alpha )^2$ as both $\alpha$ and $\lambda$ are positive, we remark that

$$\begin{aligned}{} & {} \beta (1-\alpha -\lambda )+\alpha +\lambda -(\lambda -\alpha )^2 \ge \beta (1-\alpha -\lambda )+\alpha +\lambda -(\lambda +\alpha )^2\\{} & {} \quad =\left( \beta +\lambda +\alpha \right) \left( 1-\lambda -\alpha \right) \ge 0. \end{aligned}$$

Finally, we remark that when $\alpha +\lambda =1$ we have

$$\begin{aligned} \rho (\theta +\pi )=-\exp \left( -{\textbf{i}}\frac{-\beta +\alpha -\lambda }{1+\beta }\theta -\frac{1-(\alpha -\lambda )^2}{2(1+\beta )^2}\theta ^2+{\mathcal {O}}(|\theta |^3) \right) , \text { as } \theta \rightarrow 0. \end{aligned}$$

From now on, we denote

$$\begin{aligned} c_0:= \frac{\beta +\alpha -\lambda }{1-\beta },&\quad \sigma _0:=\frac{\beta (1-\alpha -\lambda )+\alpha +\lambda -(\lambda -\alpha )^2}{2(1-\beta )^2},\\ c_\pi :=\frac{-\beta +\alpha -\lambda }{1+\beta },&\quad \sigma _\pi :=\frac{1-(\alpha -\lambda )^2}{2(1+\beta )^2}, \end{aligned}$$

and we always assume that

$$\begin{aligned} \sigma _0>0, \text { and } \sigma _\pi >0, \end{aligned}$$

which is equivalent to assume that $0<\alpha <1$ and $0<\lambda <1$.

Here, $(c_0,\sigma _0)$ and $(c_\pi ,\sigma _\pi )$ are derived, respectively, from the asymptotic expansions of the amplification factor function $\rho (\theta )$ near $\theta =0$ and $\theta =\pi$, as defined above. On the one hand $c_0$ reflects the propagation speed of the solution associated with $\rho (0)$, while $\sigma _0$ can be understood as its spatio-temporal spread (and similarly for the solution potentially associated with $\rho (\pi )$). In the following, we explore the fundamental solutions of this system for various values of its hyper-parameters.

#### Turning Off the Instantaneous Feedforward Connections: Case $\beta =0$

We first investigate the case where there is no instantaneous feedforward connections in the network, that is we set $\beta =0$. This case, although less generic, is compatible with the prominent Rao-Ballard formulation of predictive coding (Rao and Ballard 1999), in which feedforward connections—after contributing to setting the initial network activity—only convey prediction errors, as captured by the hyper-parameter $\alpha$. In that case, the model is fully explicit: the update at time step $n+1$ only depends on the internal states at the previous step *n* since we simply have

$$\begin{aligned} e_j^{n+1}= \alpha e_{j-1}^{n} +(1-\lambda -\alpha )e_j^{n}+\lambda e_{j+1}^{n}, \quad j\in {\mathbb {Z}}. \end{aligned}$$

As we assumed that $\alpha +\lambda \le 1$, the right-hand side of the recurrence equation is a positive linear combination of elements of the sequence $(e_j^{n})$ such that we have positivity principle of the solution, namely

$$\begin{aligned} \forall j\in {\mathbb {Z}}, \quad 0 \le h_j \quad \Longrightarrow \quad \forall j\in {\mathbb {Z}}, \quad n\ge 1, \quad 0\le e_j^{n}. \end{aligned}$$

Furthermore, since the recurrence equation is explicit, we have finite speed propagation, in the following sense. Recall that when $\beta =0$, the fundamental solution ${\mathcal {G}}^n$ is solution to

$$\begin{aligned} {\mathcal {G}}_j^{n+1}= \alpha {\mathcal {G}}_{j-1}^{n} +(1-\lambda -\alpha ){\mathcal {G}}_j^{n}+\lambda {\mathcal {G}}_{j+1}^{n}, \quad n\ge 1, \quad \quad j\in {\mathbb {Z}}, \end{aligned}$$

starting from ${\mathcal {G}}^0=\varvec{\delta }$. Finite speed of propagation then refers to the property that

$$\begin{aligned} {\mathcal {G}}_j^{n}=0, \quad |j|>n. \end{aligned}$$

This in turn implies that necessarily $c_0\in (-1,1)$ which is readily seen from the explicit formula $c_0=\alpha -\lambda$ in that case. Actually, it is possible to be more precise and to give a general expression for the fundamental solution. Roughly speaking, each ${\mathcal {G}}_j^n$ ressembles a discrete Gaussian distribution centered at $j=c_0n$ and we refer to the recent theoretical results of Diaconis and Saloff-Coste (2014), Randles and Saloff-Coste (2015), Coeuret (2022) and Coulombel and Faye (2022) for a rigorous justification.

Essentially, the results can be divided into two cases depending on whether or not $\alpha +\lambda =1$. As can be seen above, the special case $\alpha +\lambda =1$ results in a cancellation of the “memory” term, such that a neuronal layer *j*’s activity does not depend on its own activity at the previous time step, but only on the activity of its immediate neighbors $j-1$ and $j+1$. More precisely, we have the following:

**Fig. 3:**
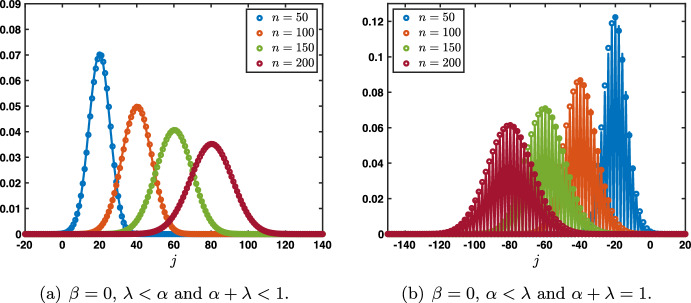
Illustration of the evolution of the fundamental solution ${\mathcal {G}}^n$ starting from the Dirac delta sequence at $j=0$ in the case $\beta =0$ at several time iterations. The circles represent the numerically computed solution while the plain lines represent the Gaussian approximation. **a** When $\lambda <\alpha$ there is a rightward propagation along a Gaussian profile whose leading profile is given by $\frac{1}{\sqrt{4\pi \sigma _0 n}}\exp \left( -\frac{|j-c_0n|^2}{4\sigma _0 n}\right)$. **b** When $\alpha <\lambda$ there is a leftward propagation along a Gaussian profile whose leading profile is given by $\frac{1+(-1)^{n+j}}{\sqrt{4\pi \sigma _0 n}}\exp \left( -\frac{|j-c_0n|^2}{4\sigma _0 n}\right)$ which vanishes whenever $n+j$ is odd (Color figure online)

**Fig. 4:**
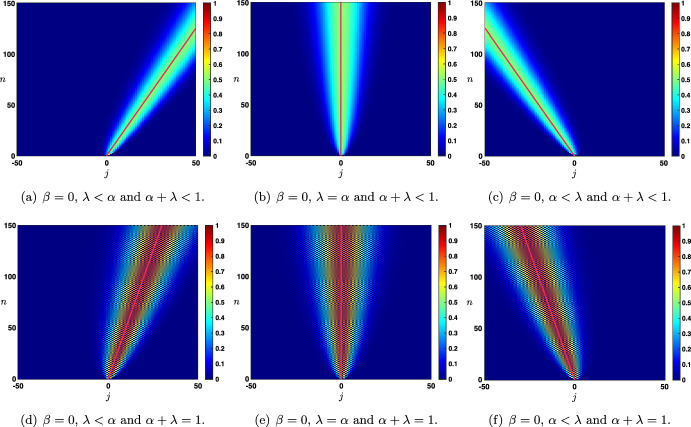
Illustration of the evolution of the rescaled solution sequence $(\sqrt{n}\,{\mathcal {G}}_j^n)_{j\in {\mathbb {Z}}}$ starting from the Dirac delta sequence at $j=0$ in the case $\beta =0$. First row: $\alpha +\lambda <1$ and second row: $\alpha +\lambda =1$. When $\lambda \lessgtr \alpha$, we observe a rightward/leftward propagation while when $\alpha =\lambda$ no propagation occurs. In all panels, the pink curve is given by $j=n c_0$, clearly illustrating the fact that $c_0$ measures the propagation speed of the solution. Note that in the case $\beta =0$ and $\alpha +\lambda =1$, we have $c_0=c_\pi$ which results in the tiled patterns observed in panels (**d**–**f**) (Color figure online)

- Case: $0\le \lambda +\alpha <1$. The fundamental solution can be decomposed as
  $$\begin{aligned} {\mathcal {G}}_j^{n}= \frac{1}{\sqrt{4\pi \sigma _0 n}}\exp \left( -\frac{|j-c_0n|^2}{4\sigma _0 n}\right) +{\mathcal {N}}_j^{n}, \quad |j|\le n, \end{aligned}$$
  where the remainder term satisfies an estimate
  $$\begin{aligned} \left| {\mathcal {N}}_j^{n}\right| \le \frac{C}{n}\exp \left( -\kappa \frac{|j-c_0n|^2}{n}\right) , \quad |j|\le n, \end{aligned}$$
  for some universal constants $C,\kappa >0$ which only depend on the hyper-parameters and not *n* and *j*. In Fig. 3a, we represented the fundamental solution ${\mathcal {G}}_j^n$ at different time iterations (circles) in the case $\lambda <\alpha$ where there is rightward propagation with $c_0>0$ and compared it with the leading order fixed Gaussian profile centered at $j=c_0n$ (plain line). On the other hand, in Fig. 4, panels (a–c), we illustrate the above results by presenting a space-time color plot of the fundamental solution rescaled by a factor $\sqrt{n}$. We observe rightward (respectively leftward) propagation with $c_0>0$ (respectively $c_0<0$) when $\lambda <\alpha$ (respectively $\alpha <\lambda$), while when $\alpha =\lambda$ we have $c_0=0$ and no propagation occurs.
- Case: $\lambda +\alpha =1$. In this case, we first note that we have $c_0=c_\pi$ together with $\sigma _0=\sigma _\pi$ and
  $$\begin{aligned} {\mathcal {G}}_j^{n}= \frac{1+(-1)^{n+j}}{\sqrt{4\pi \sigma _0 n}}\exp \left( -\frac{|j-c_0n|^2}{4\sigma _0 n}\right) +{\mathcal {N}}_j^{n}, \quad |j|\le n, \end{aligned}$$
  where the remainder term satisfies an estimate
  $$\begin{aligned} \left| {\mathcal {N}}_j^{n}\right| \le \frac{C}{n}\exp \left( -\kappa \frac{|j-c_0n|^2}{n}\right) , \quad |j|\le n, \end{aligned}$$
  for some universal constants $C,\kappa >0$. In Fig. 3b, we represented the fundamental solution ${\mathcal {G}}_j^n$ at different time iterations (circles) in the case $\alpha <\lambda$ where there is leftward propagation with $c_0<0$ and compared it with the leading order fixed Gaussian profile centered at $j=c_0n$ (plain line). Similarly as in the previous case, in Fig. 4, panels (d–f), we illustrate the above results by presenting a space-time color plot of the fundamental solution rescaled by a factor $\sqrt{n}$. The direction of propagation still depends on the sign of $c_0$ and whether or not $\lambda \lessgtr \alpha$. Unlike the case $\alpha +\lambda <1$, we observe a tiled pattern where ${\mathcal {G}}_j^{n}=0$ for even or odd integers alternatively for each time step.

As a partial intermediate summary, we note that the sign of $c_0$ (directly related to the sign of $\alpha -\lambda$) always indicates in which direction the associated Gaussian profile propagates. Namely if $\alpha >\lambda$ and $c_{0}>0$ (resp. $\alpha <\lambda$ and $c_{0}<0$) there is rightward (resp. leftward) propagation. Intuitively, this behavior reflects the functional role of each hyper-parameter, with $\alpha$ and $\lambda$ controlling feed-forward and feed-back prediction error correction, respectively. When $\alpha =\lambda$, the two terms are equally strong, and there is no dominant direction of propagation. In addition, when $\lambda +\alpha =1$, the Gaussian profile is *oscillating* because of the presence of $(-1)^{n+j}$. As will be seen later when considering continuous versions of our model, this oscillatory pattern arises here as a consequence of discrete updating.

Finally, we note that the fundamental solution sequence $({\mathcal {G}}^n_j)_{j\in {\mathbb {Z}}}$ is uniformly integrable for all values of the parameters, that is there exists some universal constant $C>0$, depending only on the hyper-parameters such that

$$\begin{aligned} \Vert {\mathcal {G}}^n\Vert _{\ell ^1({\mathbb {Z}})}:=\sum _{j\in {\mathbb {Z}}}|{\mathcal {G}}_j^n|\le C, \quad n\ge 1. \end{aligned}$$

As a consequence, since given any bounded initial sequence $(h_j)_{j\in {\mathbb {Z}}}\in \ell ^\infty ({\mathbb {Z}})$, the solution $(e_j^n)_{j\in {\mathbb {Z}}}$ to (11) can be represented as the convolution product between the initial sequence and the fundamental solution, namely

$$\begin{aligned} e_j^n=\sum _{\ell \in {\mathbb {Z}}} {\mathcal {G}}_{j-\ell }^nh_\ell , \quad j\in {\mathbb {Z}}, \quad n\ge 1, \end{aligned}$$

we readily deduce that the solution $(e_j^n)_{j\in {\mathbb {Z}}}$ is uniformly bounded with respect to *n*, that is there exists some universal constant denoted $C>0$, such that

$$\begin{aligned} \underset{j\in {\mathbb {Z}}}{\sup }\ \left| e_j^n\right| \le C~ \underset{j\in {\mathbb {Z}}}{\sup }\ \left| h_j\right| , \quad n\ge 1. \end{aligned}$$

This is exactly our definition of marginal stability.

#### Turning On the Instantaneous Feedforward Connections: Case $\beta >0$

We now turn to the general case where $\beta >0$. That is, the feed-forward connections continue to convey sensory inputs at each time step following the network initializing, and $\beta$ controls the strength of these signals. In that case, the recurrence equation is no longer explicit but implicit and the positivity property together with the finite speed propagation no longer hold true in general. Indeed, upon introducing the shift operator

$$\begin{aligned} {\textbf{S}}:(u_j)_{j\in {\mathbb {Z}}}\mapsto (u_{j+1})_{j\in {\mathbb {Z}}}, \end{aligned}$$

we remark that Eq. (11) can be written as

$$\begin{aligned} \left( \textrm{Id}-\beta {\textbf{S}}^{-1}\right) e^{n+1}=\alpha {\textbf{S}}^{-1}e^n+(1-\beta -\lambda -\alpha )e^n+\lambda {\textbf{S}}e^n, \quad n\ge 0, \end{aligned}$$

with $e^n=(e_j^n)_{j\in {\mathbb {Z}}}$. Since $0<\beta <1$ and $\Vert |{\textbf{S}}^{-1}\Vert |_{\ell ^q({\mathbb {Z}})\rightarrow \ell ^q({\mathbb {Z}})}=1$ for any $q\in [1,+\infty ]$, the operator $\textrm{Id}-\beta {\textbf{S}}^{-1}$ is invertible on $\ell ^q({\mathbb {Z}})$ for any $q\in [1,+\infty ]$ with inverse

$$\begin{aligned} \left( \textrm{Id}-\beta {\textbf{S}}^{-1}\right) ^{-1}=\sum _{\ell =0}^\infty \beta ^\ell {\textbf{S}}^{-\ell }. \end{aligned}$$

As a consequence, the recurrence equation can be recast as a convolution operator across the network layers with infinite support, namely

$$\begin{aligned}{} & {} e^{n+1}_j=\alpha \sum _{\ell =0}^\infty \beta ^\ell e^n_{j-\ell -1}+(1-\beta -\lambda -\alpha ) \sum _{\ell =0}^\infty \beta ^\ell e^n_{j-\ell }+\lambda \sum _{\ell =0}^\infty \beta ^\ell e^n_{j-\ell +1}, \\{} & {} \quad j\in {\mathbb {Z}}, \quad n\ge 0. \end{aligned}$$

From the above expression, we readily deduce that the positivity of the solution is preserved whenever $0<\beta <1-\lambda -\alpha$. Furthermore, for the fundamental solution starting from the Dirac delta solution which solves

$$\begin{aligned} {\mathcal {G}}_j^{n+1}-\beta {\mathcal {G}}_{j-1}^{n+1}= \alpha {\mathcal {G}}_{j-1}^{n} +(1-\beta -\lambda -\alpha ){\mathcal {G}}_j^{n}+\lambda {\mathcal {G}}_{j+1}^{n}, \quad j\in {\mathbb {Z}}, \quad n\ge 0, \end{aligned}$$

we only have that

$$\begin{aligned} {\mathcal {G}}_j^n=0, \quad j<-n, \end{aligned}$$

which implies that $-1<c_0,c_\pi <+\infty$. Indeed, from the formula of $c_0$ we get that

$$\begin{aligned} c_0\sim \frac{1-\alpha -\beta }{1-\beta }\longrightarrow +\infty , \quad \text { as } \beta \rightarrow 1^{-}. \end{aligned}$$

Once again, as in the case with $\beta =0$, we can characterise the behavior of the fundamental solution by using the combined results of Coeuret (2022) and Coulombel and Faye (2022).

**Fig. 5:**
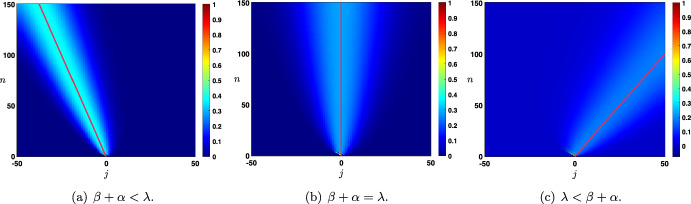
Effects of turning on $\beta >0$ when $\alpha +\lambda <1$. When $\beta +\alpha <\lambda$ we have $c_0<0$ and observe backward propagation in panel (**a**) while when $\lambda <\beta +\alpha$ we have $c_0>0$ and have forward propagation as seen in panel (**c**). At the transition $\beta +\alpha =\lambda$, the wave speed vanishes $c_0=0$ and there is no propagation as illustrated in panel (**b**). Intuitively, $\alpha$ and $\beta$, both propagating signals in the forward (rightward) direction, compete with $\lambda$ carrying the feedback (leftward) prediction signals; this competition determines the main direction of propagation of neural activity in the system (Color figure online)

**Fig. 6:**
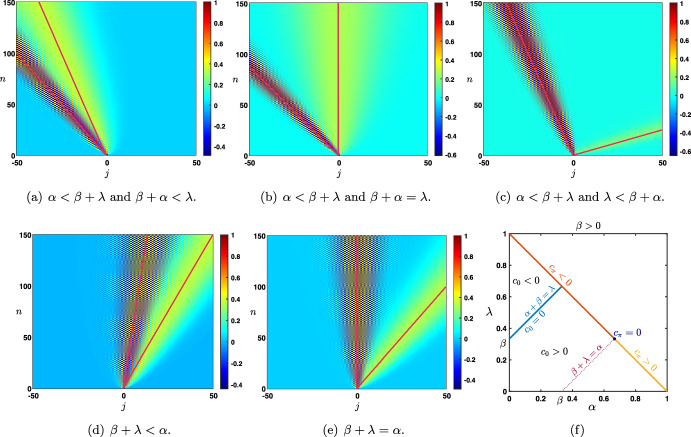
Effects of turning on $\beta >0$ when $\alpha +\lambda =1$. We now observe a secondary wave with associated wave speed $c_\pi$ whose sign depends on the competition between $\alpha$ and $\beta +\lambda$. **a**–**c** When $\alpha <\beta +\lambda$, the wave speed of the secondary wave always verifies $c_\pi <0$, and the competition between $\lambda$ and $\beta +\alpha$ gives the direction of the primary wave as previously reported in Fig. 5. **d** When $\beta +\lambda <\alpha$ which always implies that $\alpha +\beta >\lambda$, we have $0<c_\pi <c_0$ traducing forward propagation for both waves. We remark that the secondary wave is slower. **e** When $\beta +\lambda =\alpha$ which also implies that $\alpha +\beta >\lambda$, we get $0=c_\pi <c_0$ such that the secondary wave is blocked. **f** Summary of the sign of the wave speeds $c_0$ and $c_\pi$ for fixed $\beta >0$ as a function of $\alpha$ and $\beta$ with $\alpha +\lambda \le 1$ (Color figure online)

- Case: $0\le \lambda +\alpha <1$. There exist some universal constants $C,\kappa >0$ and $L>0$ such that
  $$\begin{aligned} {\mathcal {G}}_j^{n}= \frac{1}{\sqrt{4\pi \sigma _0 n}}\exp \left( -\frac{|j-c_0n|^2}{4\sigma _0 n}\right) +{\mathcal {N}}_j^{n}, \quad -n\le j \le Ln, \end{aligned}$$
  where the remainder term satisfies a Gaussian estimate
  $$\begin{aligned} \left| {\mathcal {N}}_j^{n}\right| \le \frac{C}{n}\exp \left( -\kappa \frac{|j-c_0n|^2}{n}\right) , \quad -n\le j \le Ln. \end{aligned}$$
  While for $j>nL$ we simply get a pure exponential bound
  $$\begin{aligned} \left| {\mathcal {G}}_j^{n}\right| \le C e^{-\kappa n-\kappa j }, \quad nL< j. \end{aligned}$$
  Inspecting the formula for $c_0$, we notice that when $\alpha +\beta \lessgtr \lambda$ we have $c_0\lessgtr 0$ and the wave speed vanishes precisely when $\alpha +\beta = \lambda$. This is illustrated in Fig. 5, where we see that $\alpha$ and $\beta$, both propagating signals in the forward (rightward) direction, compete with $\lambda$ carrying the feedback (leftward) prediction signals; this competition determines the main direction of propagation of neural activity in the system.
- Case: $\lambda +\alpha =1$. What changes in that case is the existence of a secondary wave with associated wave speed $c_\pi$ whose sign depends on the competition between $\alpha$ and $\beta +\lambda$. When $\alpha <\beta +\lambda$ then we have $c_\pi <0$, and the competition between $\lambda$ and $\beta +\alpha$ will determine the sign of $c_0$, as illustrated in panels (a–c) of Fig. 6. On the other hand, when $\beta +\lambda <\alpha$ implying that $c_\pi >0$, we note that $\alpha +\beta >\lambda$ and thus $c_0>0$. In that case, the explicit formula for $c_\pi$ and $c_0$ shows that $0<c_\pi <c_0$ and the secondary wave associated to $c_\pi$ is slower to propagate into the network, see Fig. 6d. Finally, when $\beta +\lambda =\alpha$ we have $0=c_\pi <c_0$ and the secondary wave is blocked, see Fig. 6e.

We have summarized in the diagram of Fig. 6f all possible configurations for the sign of the wave speeds $c_0$ and $c_\pi$ when $\beta \in (0,1)$ as a function of $\alpha$ and $\beta$ when $\alpha +\lambda \le 1$. As explained previously, $c_0$ changes sign precisely when $\lambda =\alpha +\beta$ (blue line), while the secondary wave speed $c_\pi$ only exists when $\alpha +\lambda =1$ and changes sign precisely when $\beta +\lambda =\alpha$. We notably observe that when $\beta$ is increased the region of parameter space where $c_0<0$ diminishes while the region of parameter space where $c_\pi <0$ increases, indicating that for high values of $\beta$ the primary wave is most likely to be forward while the secondary wave is most likely to be backward.

### Wave Propagation on a Semi-infinite Network with a Forcing Source Term

Now that we have understood the intrinsic underlying mechanisms of wave propagation for our model (7) set on an infinite domain, we turn to the case where the network is semi-infinite. That is, the network admits an *input* layer that is only connected to the layer above. The problem now reads

15 $$\begin{aligned} \left\{ \begin{aligned} e_j^{n+1}-\beta e_{j-1}^{n+1}&=\alpha e_{j-1}^n+(1-\beta -\lambda - \alpha )e_j^{n} + \lambda e_{j+1}^{n}, \quad j\ge 1, \quad n\ge 0,\\ e_0^n&=s_0^n, \quad n\ge 0,\\ e_j^0&=h_j, \quad j\ge 1. \end{aligned} \right. \end{aligned}$$

We see that the system depends on the source term $s_0^n$ applied to its input layer at each time step, also called a *boundary value*, and on the starting activation value $(h_j)$ applied to each layer at the initial time point, also called the *initial value*. In fact, the linearity principle tells us that the solutions of the above problem can be obtained as the linear superposition of the solutions to the following two problems, the boundary value problem, where all layers except the input layer are initialized at zero:

16 $$\begin{aligned} \left\{ \begin{aligned} g_j^{n+1}-\beta g_{j-1}^{n+1}&=\alpha g_{j-1}^n+(1-\beta -\lambda - \alpha )g_j^{n} + \lambda g_{j+1}^{n}, \quad j\ge 1, \quad n\ge 0,\\ g_0^n&=s_0^n, \quad n\ge 0,\\ g_j^0&=0, \quad j\ge 1, \end{aligned} \right. \end{aligned}$$

and the initial value problem, where the input layer source term is set to zero for all time steps:

17 $$\begin{aligned} \left\{ \begin{aligned} f_j^{n+1}-\beta f_{j-1}^{n+1}&=\alpha f_{j-1}^n+(1-\beta -\lambda - \alpha )f_j^{n} + \lambda f_{j+1}^{n}, \quad j\ge 1, \quad n\ge 0,\\ f_0^n&=0, \quad n\ge 0,\\ f_j^0&=h_j, \quad j\ge 1. \end{aligned} \right. \end{aligned}$$

Subsequently, the generic solution sequence $(e_j^n)_{j\ge 1}$ can be obtained as

$$\begin{aligned} e_j^n=f_j^n+g_j^n, \quad j\ge 1, \quad n\ge 1. \end{aligned}$$

#### The Initial Value Problem (17)

It is first natural to investigate the initial value problem (17) since it is really close to the infinite network case of the previous section. Here, we consider the effect of the initial value assigned to each layer $j>0$ at the first time step ($n=0$), except the input layer ($j=0$) which is set to zero. The dynamics of (17) is still read out from the amplification factor function $\rho$ defined in (12) and once again the solutions to (17) can be obtained as the convolution of the initial sequence with the fundamental solution associated to the problem. For $j_0\ge 1$, we denote by $\varvec{\delta }^{j_0}$ the Dirac delta sequence defined as $\varvec{\delta }_{j_0}^{j_0}=1$ and $\varvec{\delta }_j^{j_0}=0$ for all $j\ge 1$ and $j\ne j_0$. Correspondingly, we denote by ${\mathcal {G}}^n_{\textrm{ivp}}(\cdot ,j_0)=({\mathcal {G}}^n_{\textrm{ivp}}(j,j_0))_{j\ge 1}$ the solution to (17) starting from $\varvec{\delta }^{j_0}$, and let us remark that the solutions to (17) starting from any initial condition $(h_j)_{j\ge 1}$ can be represented as

$$\begin{aligned} f_j^n=\sum _{j_0=1}^{+\infty }{\mathcal {G}}^n_{\textrm{ivp}}(j,j_0)h_{j_0}, \quad j\ge 1, \quad n\ge 1. \end{aligned}$$

**Fig. 7:**
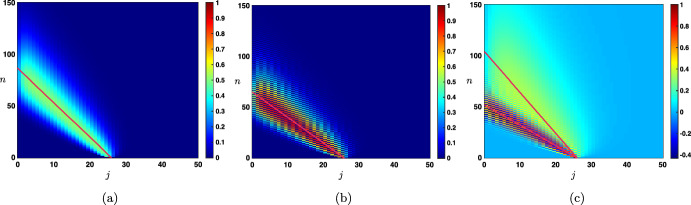
Space-time evolution of the rescaled solution sequence $(\sqrt{n} \, {\mathcal {G}}^n_{\textrm{ivp}}(j,j_0))_{j\ge 1}$ to (17) starting with a Dirac delta sequence at $j_0=25$ in different cases with leftward propagation. **a** $\beta =0$ & $\alpha +\lambda <1$ with $c_0<0$. **b** $\beta =0$ & $\alpha +\lambda =1$ with $c_0<0$. **c** $0<\beta <1$ & $\alpha +\lambda =1$ with $-1<c_\pi<c_0<0$ (Color figure online)

Combining the results of Coulombel and Faye (2022) and Coeuret (2022) together with those of Coulombel and Faye (2021), Coeuret (2023), Goldberg and Tadmor (1985, 1987) which precisely deal with recurrence equations with boundary conditions, one can obtain very similar results as in the previous case. The very first obvious remark that we can make is that for all $j,j_0\ge 1$ and $1\le n<j_0$ we have

$$\begin{aligned} {\mathcal {G}}^n_{\textrm{ivp}}(j,j_0)={\mathcal {G}}^n_{j-j_0}, \end{aligned}$$

meaning that it takes $n=j_0$ iterations before the solution arrives at the boundary $j=0$ and for $1\le n<j_0$ the problem is similar to the one set on the infinite network. This behavior is illustrated in Fig. 7 for several values of the hyper-parameters where we represent the spatio-temporal evolution of the rescaled solution sequence $(\sqrt{n} \, {\mathcal {G}}^n_{\textrm{ivp}}(j,j_0))_{j\ge 1}$. We clearly observe a Gaussian behavior before the solution reaches the boundary. And for all $n\ge j_0$, we can write

$$\begin{aligned} {\mathcal {G}}^n_{\textrm{ivp}}(j,j_0)={\mathcal {G}}^n_{j-j_0}+{\mathcal {G}}_{\textrm{bl}}^n(j,j_0), \end{aligned}$$

where ${\mathcal {G}}_{\textrm{bl}}^n(j,j_0)$ is a remainder term generated by the boundary condition at $j=0$. It is actually possible to bound ${\mathcal {G}}_{\textrm{bl}}^n(j,j_0)$ in each of the cases treated above.

**Fig. 8:**
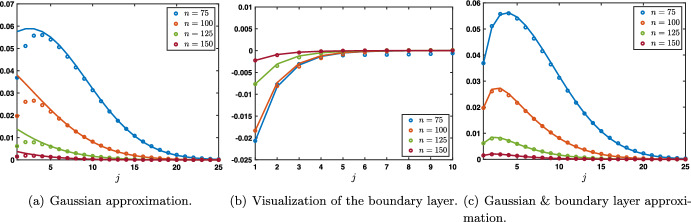
Illustration of the solution ${\mathcal {G}}^n_{\textrm{ivp}}(j,j_0)$ to (17) in the case where $\beta =0$ and $\alpha +\lambda <1$ with $\alpha <\beta$. **a** Visualizations of the solution ${\mathcal {G}}^n_{\textrm{ivp}}(j,j_0)$ (circles) at different time iterations. The plain lines correspond to the Gaussian approximation $\frac{1}{\sqrt{4\pi \sigma _0 n}}\exp \left( -\frac{|j-j_0-c_0n|^2}{4\sigma _0 n}\right)$ and remark the presence of a boundary layer (seen as a mismatch between the circles and the Gaussian lines approximation). **b** We represent the boundary layer by plotting (circles) ${\mathcal {G}}^n_{\textrm{ivp}}(j,j_0)-\frac{1}{\sqrt{4\pi \sigma _0 n}}\exp \left( -\frac{|j-j_0-c_0n|^2}{4\sigma _0 n}\right)$ and we compare it to our boundary layer approximation $-\frac{1}{\sqrt{4\pi \sigma _0 n}}\exp \left( -\frac{|-j_0-c_0n|^2}{4\sigma _0 n}\right) \left( \frac{\alpha }{\lambda }\right) ^j$ (plain lines). **c** Finally we compare the solution ${\mathcal {G}}^n_{\textrm{ivp}}(j,j_0)$ (circles) to its first order approximation $\frac{1}{\sqrt{4\pi \sigma _0 n}}\exp \left( -\frac{|j-j_0-c_0n|^2}{4\sigma _0 n}\right) -\frac{1}{\sqrt{4\pi \sigma _0 n}}\exp \left( -\frac{|-j_0-c_0n|^2}{4\sigma _0 n}\right) \left( \frac{\alpha }{\lambda }\right) ^j$ (plain lines) (Color figure online)

When $\beta =0$ and $\alpha +\lambda <1$ with $\alpha <\lambda$ such that $c_0<0$, then ${\mathcal {G}}_{\textrm{bl}}^n(j,j_0)$ is well approximated by

$$\begin{aligned} {\mathcal {G}}_{\textrm{bl}}^n(j,j_0)\approx \left\{ \begin{array}{lc} -\frac{1}{\sqrt{4\pi \sigma _0 n}}\exp \left( -\frac{|-j_0-c_0n|^2}{4\sigma _0 n}\right) \left( \frac{\alpha }{\lambda }\right) ^j,&{} \quad 1\le j \le j_0,\\ e^{-\kappa n -\kappa (j-j_0)}, &{} \quad j>j_0, \end{array} \right. \end{aligned}$$

while when $\lambda <\alpha$ with $c_0>0$, then ${\mathcal {G}}_{\textrm{bl}}^n(j,j_0)$ is well approximated by

$$\begin{aligned} {\mathcal {G}}_{\textrm{bl}}^n(j,j_0)\approx \left\{ \begin{array}{lc} e^{-\kappa n -\kappa (j_0-j)}, &{} \quad 1\le j \le j_0,\\ -\frac{1}{\sqrt{4\pi \sigma _0 n}}\exp \left( -\frac{|j-c_0n|^2}{4\sigma _0 n}\right) \left( \frac{\lambda }{\alpha }\right) ^{j_0},&{} \quad j_0< j, \end{array} \right. \end{aligned}$$

this is illustrated in Fig. 8 in the case $c_0<0$.

On the other hand for $\alpha +\lambda =1$ with $\alpha <\lambda$ such that $c_0<0$, then ${\mathcal {G}}_{\textrm{bl}}^n(j,j_0)$ is well approximated by

$$\begin{aligned} {\mathcal {G}}_{\textrm{bl}}^n(j,j,_0)\approx \left\{ \begin{array}{lc} -\frac{1+(-1)^n}{\sqrt{4\pi \sigma _0 n}}\exp \left( -\frac{|-j_0-c_0n|^2}{4\sigma _0 n}\right) \left( \frac{\alpha }{\lambda }\right) ^j,&{} \quad 1\le j \le j_0,\\ e^{-\kappa n -\kappa (j-j_0)}, &{} \quad j>j_0, \end{array} \right. \end{aligned}$$

while when $\lambda <\alpha$ with $c_0>0$, then ${\mathcal {G}}_{\textrm{bl}}^n(j,j_0)$ is well approximated by

$$\begin{aligned} {\mathcal {G}}_{\textrm{bl}}^n(j,j_0)\approx \left\{ \begin{array}{lc} e^{-\kappa n -\kappa (j_0-j)}, &{} \quad 1\le j \le j_0,\\ -\frac{1+(-1)^n}{\sqrt{4\pi \sigma _0 n}}\exp \left( -\frac{|j-c_0n|^2}{4\sigma _0 n}\right) \left( \frac{\lambda }{\alpha }\right) ^{j_0},&{} \quad j_0< j. \end{array} \right. \end{aligned}$$

When $0<\beta <1$ and $\alpha +\lambda <1$ the approximations are similar as for the case with $\beta =0$. We thus need to discuss three cases.

- Case $-1<c_\pi<c_0<0$. In that case, we have for $1\le j \le j_0$ that
  $$\begin{aligned} {\mathcal {G}}_{\textrm{bl}}^n(j,j_0){} & {} \approx -\frac{1}{\sqrt{4\pi \sigma _0 n}}\exp \left( -\frac{|-j_0-c_0n|^2}{4\sigma _0 n}\right) \left( \frac{\alpha +\beta }{\lambda }\right) ^j\\{} & {} \quad -\frac{(-1)^n}{\sqrt{4\pi \sigma _\pi n}}\exp \left( -\frac{|-j_0-c_\pi n|^2}{4\sigma _\pi n}\right) \left( \frac{\alpha -\beta }{\lambda }\right) ^j, \end{aligned}$$
  with an exponential bound for $j>j_0$. This situation is presented in Fig. 7c
- Case $-1<c_\pi<0<c_0$. In this case we have
  $$\begin{aligned} {\mathcal {G}}_{\textrm{bl}}^n(j,j_0)\approx \left\{ \begin{array}{lc} -\frac{(-1)^n}{\sqrt{4\pi \sigma _\pi n}}\exp \left( -\frac{|-j_0-c_\pi n|^2}{4\sigma _\pi n}\right) \left( \frac{\alpha -\beta }{\lambda }\right) ^j,&{} \quad 1\le j \le j_0,\\ -\frac{1}{\sqrt{4\pi \sigma _0 n}}\exp \left( -\frac{|j-c_0n|^2}{4\sigma _0 n}\right) \left( \frac{\lambda }{\alpha +\beta }\right) ^{j_0}, &{} \quad j_0<j<Ln. \end{array} \right. \end{aligned}$$
- Case $-1<0<c_\pi <c_0$. In this case we have
  $$\begin{aligned} {\mathcal {G}}_{\textrm{bl}}^n(j,j_0){} & {} \approx -\frac{1}{\sqrt{4\pi \sigma _0 n}}\exp \left( -\frac{|j-c_0n|^2}{4\sigma _0 n}\right) \left( \frac{\lambda }{\alpha +\beta }\right) ^{j_0}\\{} & {} \quad -\frac{(-1)^n}{\sqrt{4\pi \sigma _\pi n}}\exp \left( -\frac{|j-c_\pi n|^2}{4\sigma _\pi n}\right) \left( \frac{\lambda }{\alpha -\beta }\right) ^{j_0} \end{aligned}$$
  for $$j_0<j<Ln$$.

#### The Boundary Value Problem (16)

We now turn our attention to the boundary value problem (16) where the network is initialized with zero activity, for all layers except the input. Motivated by applications, we will only focus on the case where $s_0^n=s_0\in {\mathbb {R}}$ for all $n\ge 0$ (i.e., a constant sensory input) and thus study:

18 $$\begin{aligned} \left\{ \begin{aligned} g_j^{n+1}-\beta g_{j-1}^{n+1}&=\alpha g_{j-1}^n+(1-\beta -\lambda - \alpha )g_j^{n} + \lambda g_{j+1}^{n}, \quad j\ge 1, \quad n\ge 0,\\ g_0^n&=s_0, \quad n\ge 0,\\ g_j^0&=0, \quad j\ge 1. \end{aligned} \right. \end{aligned}$$

**Case** $\beta =0$. Here, the stimulus information $s_o$ does not directly propagate through the network via its feedforward connections (since $\beta =0$), but may still propagate towards higher layers $j>0$ via the feedforward prediction error correction mechanism, governed by parameter $\alpha$. When $\alpha +\lambda \le 1$, we distinguish between three cases. Here and throughout, we denote by $\textrm{erf}$ the error function defined by

$$\begin{aligned} \textrm{erf}(x):=\frac{2}{\sqrt{\pi }}\int _0^x e^{-z^2}\textrm{d}z, \quad x\in {\mathbb {R}}. \end{aligned}$$

**Fig. 9:**
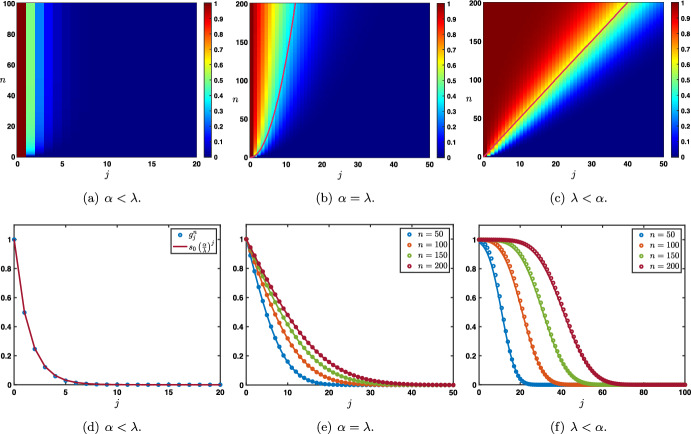
Case $\beta =0$ and $\alpha +\lambda \le 1$. Visualization of the solution $(g_j^n)_{j\ge 1}$ of (18) when $s_0^n=1$ for all $n\ge 0$. Top row: space-time plots of the solution depending on $\alpha$ and $\lambda$. Bottom row: solution profiles at different time iterations (circles) compared with the leading order approximations. In the case $\alpha <\lambda$, we observe uniform convergence (in space) at exponential rate (in time) toward the stationary solution $s_0 \left( \left( \frac{\alpha }{\lambda }\right) ^j\right) _{j\ge 1}$ while when $\lambda <\alpha$ we observe the propagation of a wavefront where the uniform steady state $s_0$ is propagating at speed $c_0>0$. In the intermediate case where $\alpha =\lambda$ we get a diffusive invasion as illustrated by the curve $j=\sqrt{4\sigma _0n}$ (Color figure online)

- Case $\alpha <\lambda$. In this case we have
  $$\begin{aligned} g_j^n =s_0 \left( \frac{\alpha }{\lambda }\right) ^j\left( 1+\omega _j^n \right) , \quad \text { with } \quad \left| \omega _j^n \right| \le C e^{-\kappa n -\kappa j}, \quad j\ge 1, \quad n \ge 1. \end{aligned}$$
  It is interesting to note that the sequence $\left( s_0 \left( \frac{\alpha }{\lambda }\right) ^j \right) _{j\le 1}$ is a stationary solution to (18) and we have uniform convergence at exponential rate toward this stationary solution, that is
  $$\begin{aligned} \underset{j\ge 1}{\sup }\left| g_j^n -s_0 \left( \frac{\alpha }{\lambda }\right) ^j\right| \le C e^{-\kappa n} \underset{n\rightarrow +\infty }{\longrightarrow }0. \end{aligned}$$
  We illustrate this uniform convergence in Fig. 9a and d.
- Case $\alpha =\lambda$. We have
  $$\begin{aligned} \left| g_j^n -s_0\left( 1-\textrm{erf}\left( \frac{j}{\sqrt{4\sigma _0 n}} \right) \right) \right| \le \frac{C}{n}\exp \left( -\kappa \frac{j^2}{n}\right) , \quad j\ge 1, \quad n \ge 1. \end{aligned}$$
  In this case, we observe a slow convergence to the steady state $s_0$. Indeed, for each $\delta \in (0,1/2)$ we have
  $$\begin{aligned} \underset{n\rightarrow +\infty }{\lim }\, \underset{1\le j \le n^{\delta }}{\sup }\,\left| g_j^n -s_0\right| =0, \end{aligned}$$
  while for any $\delta >1/2$ we get
  $$\begin{aligned} \underset{n\rightarrow +\infty }{\lim }\, \underset{j \ge n^{\delta }}{\sup }\,\left| g_j^n \right| =0. \end{aligned}$$
  The propagation is thus diffusive along $j \sim \sqrt{n}$. This can be seen in Fig. 9b and e.
- Case $\lambda <\alpha$. In this case we have
  $$\begin{aligned} \left| g_j^n -\frac{s_0}{2}\left( 1-\textrm{erf}\left( \frac{j-c_0n}{\sqrt{4\sigma _0 n}} \right) \right) \right| \le \frac{C}{\sqrt{n}}\exp \left( -\kappa \frac{(j-c_0n)^2}{n}\right) , \quad j\ge 1, \quad n \ge 1. \end{aligned}$$
  In this case, we deduce that we have local uniform convergence towards the steady state $s_0$, actually we have spreading at speed $c_0$. More precisely, for any $c\in (0,c_0)$ we have
  $$\begin{aligned} \underset{n\rightarrow +\infty }{\lim }\, \underset{1\le j \le cn}{\sup }\,\left| g_j^n -s_0\right| =0, \end{aligned}$$
  while for any $c>c_0$, we get
  $$\begin{aligned} \underset{n\rightarrow +\infty }{\lim }\, \underset{ j \ge cn }{\sup }\,\left| g_j^n\right| =0. \end{aligned}$$
  We refer to Fig. 9c and f for an illustration. The figure clearly shows the competition between hyperparameters $\alpha$ and $\lambda$, with forward propagation of the sensory input only when $\alpha \ge \lambda$.

**Case** $0<\beta <1$. Here, the stimulus information $s_o$ propagates through the network not only via its feedforward connections (governed by $\beta >0$) but also via the feedforward prediction error correction mechanism, governed by parameter $\alpha$. In the case where $\alpha +\lambda \le 1$, the results from the case $\beta =0$ remain valid, the only differences coming from the fact that the above approximations in the case $\lambda \le \alpha$ are only valid for $1\le j \le Ln$ for some large constant $L>0$ with exponential localized bounds for $j\ge Ln$ and that the steady state is now $\left( s_0 \left( \frac{\alpha +\beta }{\lambda }\right) ^j\right) _{j\ge 1}$ whenever $\alpha +\beta <\lambda$. This confirms that the feedforward propagation of the input $s_0$ is now dependent on both terms $\alpha$ and $\beta$, jointly competing against the feedback term $\lambda$.

Let us remark that when $0<\beta <\alpha -\lambda$ and in the special case $\alpha +\lambda =1$, where a second stable point exists for the amplification factor function at $\rho (\pi )$, we can get a slightly more accurate description of the solution in the form

19 $$\begin{aligned} g_j^n= & {} \frac{s_0}{2}\left( 1-\textrm{erf}\left( \frac{j-c_0n}{\sqrt{4\sigma _0 n}} \right) \right) \nonumber \\{} & {} -\frac{s_0}{2(1+\beta )}\frac{(-1)^j}{\sqrt{4\pi \sigma _\pi n}}\exp \left( - \frac{(j-c_\pi n)^2}{4\sigma _\pi n}\right) +r_j^n, \quad n\ge 1, \quad 1\le j \le Ln,\nonumber \\ \end{aligned}$$

where the remainder term satisfies an estimate of the form

$$\begin{aligned} \left| r_j^n\right| \le \frac{C}{\sqrt{n}}\exp \left( -\kappa \frac{(j-c_0n)^2}{n}\right) +\frac{C}{n}\exp \left( -\kappa \frac{(j-c_\pi n)^2}{n}\right) . \end{aligned}$$

**Fig. 10:**
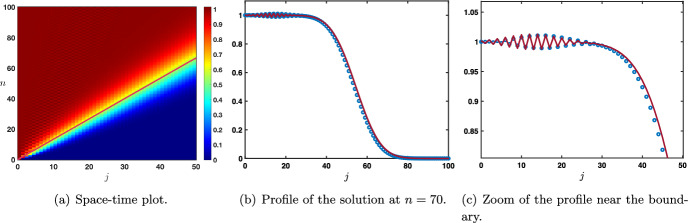
Case $0<\beta <1$ and $\alpha +\lambda =1$ with $0<\beta <\alpha -\lambda$. Visualization of the solution $(g_j^n)_{j\ge 1}$ of (18) when $s_0^n=1$ for all $n\ge 0$. **a** Space-time plot of the solution. **b** and **c** Solution profile at different time iterations (circles) compared with the leading order approximation (dark red line) from (19). The solution is the super-position of a leading rightward front spreading at speed $c_0>0$ and an oscillatory Gaussian profile propagating at speed $c_\pi >0$ with $c_\pi <c_0$ (Color figure online)

This is illustrated in Fig. 10. It should be noted here that, while the main wavefront reflecting solution $c_0$ is a generic property of our network in the entire range of validity of parameters $0\le \beta <1$ and $\alpha +\lambda \le 1$, the second oscillatory pattern reflecting $c_{\pi }$ only appears in the special case of $\beta \ne 0$ and $\alpha +\lambda =1$. This oscillation is, in fact, an artifact from the discrete formulation of our problem, as will become evident in the next section, where we investigate continuous formulations of the problem.

### Towards Continuous Predictive Models

Starting from a discrete approximation of our system made sense, not only for mathematical convenience but also because artificial neural networks and deep learning systems implementing similar predictive coding principles are intrinsically discrete. Nonetheless, it can be useful to discard this discrete approximation and investigate our system in the continuous limit. Note that in the following, we will explore continuous extensions of our model in both time *and* space. Biological neural networks, like any physical system, operate in continuous time and thus it is more biologically accurate to relax the temporal discretization assumption. This is what we do in the first part of this section. In the spatial domain, however, the discretization of our system into successive processing layers was not just an approximation, but also a reflection of the hierarchical anatomy of the brain. Nonetheless, we can still represent neuronal network depth continuously, even if only as a mathematical abstraction. This is what we will do in the subsequent part of this section. Understanding such continuous limits can allow us to test the robustness of our framework, and to relate it to canonical models whose dynamics have been more exhaustively characterized.

#### Continuous in Time Interpretation

As a first step, we present a continuous in time interpretation of the model (15). We let $\Delta t>0$ be some parameter which will represent a time step and reformulate the recurrence equation as

$$\begin{aligned} (1-\beta )\frac{e_j^{n+1}-e_j^n}{\Delta t}=\frac{\beta }{\Delta t} \left( e_{j-1}^{n+1} -e_j^{n+1}\right) + \frac{\lambda }{\Delta t} \left( e_{j+1}^{n}-e_j^{n}\right) -\frac{\alpha }{\Delta t} \left( e_{j}^{n}-e_{j-1}^{n}\right) . \end{aligned}$$

We now interpret $e_j^n$ as the approximation of some smooth function of time $\textbf{e}_j(t)$ evaluated at $t_n:=n \Delta t$, that is $e_j^n \sim \textbf{e}_j(t_n)$. As a consequence, we get that

$$\begin{aligned} e_j^{n+1} \sim \textbf{e}_j(t_{n+1})=\textbf{e}_j(t_{n}+\Delta t)=\textbf{e}_j(t_{n})+\Delta t \frac{\textrm{d}}{\textrm{d}t}\textbf{e}_j(t_{n})+{\mathcal {O}}(|\Delta t|^2), \text { as } \Delta t \rightarrow 0, \end{aligned}$$

such that

$$\begin{aligned} \frac{e_j^{n+1}-e_j^n}{\Delta t} \sim \frac{\textrm{d}}{\textrm{d}t}\textbf{e}_j(t_{n}), \text { as } \Delta t \rightarrow 0. \end{aligned}$$

Now, introducing the scaled parameters

$$\begin{aligned} {\widetilde{\beta }}:=\frac{\beta }{\Delta t}, \quad {\widetilde{\lambda }}:=\frac{\lambda }{\Delta t}, \text { and } {\widetilde{\alpha }}:=\frac{\alpha }{\Delta t}, \end{aligned}$$

we get at the limit $\Delta t\rightarrow 0$ the following lattice ordinary differential equation

20 $$\begin{aligned} \frac{\textrm{d}}{\textrm{d}t}\textbf{e}_j(t)=({\widetilde{\beta }}+{\widetilde{\alpha }})\textbf{e}_{j-1}(t)-({\widetilde{\beta }}+{\widetilde{\alpha }}+{\widetilde{\lambda }})\textbf{e}_j(t)+{\widetilde{\lambda }}{} \textbf{e}_{j+1}(t), \quad t>0. \end{aligned}$$

When defined on the infinite lattice ${\mathbb {Z}}$, one can represent the solutions as

$$\begin{aligned} \textbf{e}_j(t)=\sum _{k\in {\mathbb {Z}}}{\textbf{G}}_{j-k}(t)h_k, \quad j\in {\mathbb {Z}}, \quad t>0, \end{aligned}$$

starting from the initial sequence $\textbf{e}_j(t=0)=(h_j)_{j\in {\mathbb {Z}}}$ where $({\textbf{G}}_{j}(t))_{j\in {\mathbb {Z}}}$ is the fundamental solution to (20) starting from the Dirac delta sequence $\varvec{\delta }$. Once again, each ${\textbf{G}}_{j}(t)$ can be represented by the inverse Fourier transform and reads

$$\begin{aligned} {\textbf{G}}_{j}(t)=\frac{1}{2\pi }\int _{-\pi }^{\pi } e^{\nu (\theta )t}e^{{\textbf{i}}j \theta }\textrm{d}\theta , \quad t>0, \quad j\in {\mathbb {Z}}, \end{aligned}$$

where the function $\nu (\theta )$ is defined as

$$\begin{aligned} \nu (\theta ):=({\widetilde{\beta }}+{\widetilde{\alpha }})e^{-{\textbf{i}}\theta }-({\widetilde{\beta }}+{\widetilde{\alpha }}+{\widetilde{\lambda }})+{\widetilde{\lambda }}e^{{\textbf{i}}\theta }, \quad \theta \in [-\pi ,\pi ]. \end{aligned}$$

The function $\nu (\theta )$ serves as an amplification factor function for the time continuous Eq. (20). To ensure stability,^1^ one needs to impose that $\textrm{Re}(\nu (\theta ))\le 0$ for each $\theta \in [-\pi ,\pi ]$. From its formula, we obtain that

$$\begin{aligned} \textrm{Re}(\nu (\theta ))=({\widetilde{\beta }}+{\widetilde{\alpha }}+{\widetilde{\lambda }})(\cos (\theta )-1), \quad \theta \in [-\pi ,\pi ], \end{aligned}$$

such that we deduce that $\textrm{Re}(\nu (0))=0$ and $\textrm{Re}(\nu (\theta ))<0$ for all $\theta \in [-\pi ,\pi ]\backslash \{0\}$. In particular, it is now evident that, contrary to the discrete case, $\nu (\pi )$ cannot be a stable solution for the continuous system (except in the trivial case where all hyperparameters ${\widetilde{\alpha }},{\widetilde{\beta }},{\widetilde{\lambda }}$ are zero). This confirms that the previously observed oscillations associated with $\rho (\pi )$ in specific cases were merely an artifact of the temporal discretization.

We note that, near the tangency point at $\theta =0$, the function $\nu (\theta )$ has the following asymptotic expansion

$$\begin{aligned} \nu (\theta )=-({\widetilde{\beta }}+{\widetilde{\alpha }}+{\widetilde{\lambda }}){\textbf{i}}\theta -\frac{{\widetilde{\beta }}+{\widetilde{\alpha }}+{\widetilde{\lambda }}}{2}\theta ^2+{\mathcal {O}}(|\theta |^3), \text { as } \theta \rightarrow 0. \end{aligned}$$

It is also possible to prove a Gaussian approximation in that case, and following for example (Besse et al. 2022), we have

$$\begin{aligned} {\textbf{G}}_{j}(t)=\frac{1}{\sqrt{4\pi \widetilde{\sigma _0}t}}\exp \left( -\frac{|j-\widetilde{c_0}t|^2}{4\widetilde{\sigma _0}t}\right) +{\textbf{R}}_{j}(t), \quad j\in {\mathbb {Z}}, \quad t>0, \end{aligned}$$

with

$$\begin{aligned} \left| {\textbf{R}}_{j}(t) \right| \le \frac{C}{\sqrt{t}}\exp \left( -\kappa \frac{|j-\widetilde{c_0}t|^2}{t}\right) , \quad j\in {\mathbb {Z}}, \quad t>0, \end{aligned}$$

for some universal constants $C>0$ and $\kappa >0$. Here, $\widetilde{c_0}$ and $\widetilde{\sigma _0}$ are given by

$$\begin{aligned} \widetilde{c_0}={\widetilde{\beta }}+{\widetilde{\alpha }}-{\widetilde{\lambda }}, \quad \text { and } \quad \widetilde{\sigma _0}=\frac{{\widetilde{\beta }}+{\widetilde{\alpha }}+{\widetilde{\lambda }}}{2}>0. \end{aligned}$$

We remark that both $\widetilde{c_0}$ and $\widetilde{\sigma _0}$ are linked to $c_0$ and $\sigma _0$ (the propagation speed and spread of the solution in the case of the discrete model) in the following sense

$$\begin{aligned} \frac{c_0}{\Delta t}\rightarrow \widetilde{c_0}, \quad \text { and } \quad \frac{\sigma _0}{\Delta t}\rightarrow \widetilde{\sigma _0}, \quad \text { as } \Delta t\rightarrow 0. \end{aligned}$$

We also note that the spatially homogeneous solutions of (20) are trivial in the sense that if we assume that $\textbf{e}_j(t)=\textbf{e}(t)$ for all $j\in {\mathbb {Z}}$ then the equation satisfied by $\textbf{e}(t)$ is simply

$$\begin{aligned} \frac{\textrm{d}}{\textrm{d}t}{} \textbf{e}(t)=0. \end{aligned}$$

Finally, we conclude by noticing that in this continuous in time regime, there is no possible oscillations either in space or time, in the sense that the fundamental solution always resembles a fixed Gaussian profile advected at wave speed $\widetilde{c_0}$. The formula for $\widetilde{c_0}$ highlights the intuitive functional relation between the propagation (or advection) direction and the “competition” between the feedforward influences ${\widetilde{\alpha }}+{\widetilde{\beta }}$ and the feedback influence ${\widetilde{\lambda }}$.

#### Fully Continuous Interpretation: Both in Time and Depth

In this section, we give a possible physical interpretation of the discrete model (15) via continuous transport equations, in which both time and space (i.e., neuronal network depth) are made continuous. Let us introduce $\Delta t>0$, $\Delta x>0$ and set $\nu :=\frac{\Delta x}{\Delta t}$. As before, we can view ${\Delta t}$ as a time step for our system; additionally, ${\Delta x}$ can be viewed as a spatial step in the (continuous) neuronal depth dimension, and thus $\nu$ becomes akin to a neural propagation speed or a conduction velocity. We then rewrite the recurrence equation as

$$\begin{aligned} (1-\beta )\frac{e_j^{n+1}-e_j^n}{\Delta t}=\beta \nu \frac{e_{j-1}^{n+1} -e_j^{n+1}}{\Delta x}+ \lambda \nu \frac{ e_{j+1}^{n}-e_j^{n}}{\Delta x}-\alpha \nu \frac{ e_{j}^{n}-e_{j-1}^{n}}{\Delta x}. \end{aligned}$$

The key idea is to now assume that $e_j^n$ represents an approximation of some smooth function $\textbf{e}(t,x)$ evaluated at $t_n:=n \Delta t$ and $x_j:=j \Delta x$, that is $e_j^n \sim \textbf{e}(t_n,x_j)$. Then passing to the limit $\Delta t\rightarrow 0$, $\Delta x \rightarrow 0$ with $\frac{\Delta x}{\Delta t}=\nu >0$ fixed and assuming that $\beta +\alpha \ne \lambda$, one gets the partial differential equation

21 $$\begin{aligned} \partial _t \textbf{e}(t,x) + \frac{\nu (\beta +\alpha -\lambda )}{1-\beta } \partial _x \textbf{e}(t,x) =0, \quad t>0, \quad x>0, \end{aligned}$$

with boundary condition $\textbf{e}(t,x=0)=s_0(t)$ and initial condition $\textbf{e}(t=0,x)=h(x)$ satisfying the compatibility condition $s_0(0)=h(0)$ where $s_0(t)$ is a smooth function such that $s_0(t^n)=s_0^n$ and $h(x_j)=h_j$. The above partial differential equation is a transport equation with associated speed $\frac{\nu (\beta +\alpha -\lambda )}{1-\beta }=\nu c_0$. Depending on the sign of $c_0$, we have a different representation for the solutions of (21).

- Case $c_0<0$. Solution is given by
  $$\begin{aligned} \textbf{e} (t,x)=h\left( x- \nu c_0 t\right) , \quad t>0, \quad x>0. \end{aligned}$$
  Let us remark that when $c_0<0$ the trace of the solution at $x=0$ is entirely determined by the initial data *h*(*x*) since
  $$\begin{aligned} \textbf{e} (t,x=0)=h\left( - \nu c_0 t\right) , \quad t>0. \end{aligned}$$
  Intuitively, this reflects the dominance of backward (leftward) propagation in this network, with solutions determined entirely by the initial value *h*(*x*), even for $x=0$ (the source term, $s_0(t)$, having no influence in this case).
- Case $c_0>0$. Solution is given by
  $$\begin{aligned} \textbf{e} (t,x)=\left\{ \begin{array}{lc} s_0\left( t-\frac{x}{\nu c_0}\right) , &{} x \le \nu c_0 t, \\ h\left( x- \nu c_0 t\right) , &{} x> \nu c_0 t, \end{array} \right. \quad t>0, \quad x>0. \end{aligned}$$
  Intuitively, this reflects the dominance of forward (rightward) propagation in this network, with both the source term $s_0(t)$ and the initial values *h*(*x*) transported at constant velocity $\nu c_0$.

Thanks to the explicit form of the solutions, we readily obtain many qualitative properties of the solution $\textbf{e} (t,x)$. Boundedness and positivity of the solutions are inherited from the functions $s_0(t)$ and *h*(*x*). In the case where $\beta +\alpha = \lambda$ (i.e., with balanced feed-forward and feedback influences), the limiting equation is slightly different. Indeed, in this case, introducing $\delta :=\frac{\Delta x^2}{\Delta t}$, which can be interpreted as an effective diffusivity, and letting $\Delta t\rightarrow 0$, $\Delta x \rightarrow 0$ with $\delta >0$ fixed, one gets the partial differential equation

22 $$\begin{aligned} \partial _t \textbf{e}(t,x) = \frac{\delta (\beta +\alpha +\lambda )}{2(1-\beta ) } \partial _x^2 \textbf{e}(t,x), \quad t>0, \quad x>0, \end{aligned}$$

and we readily observe that when $\beta +\alpha = \lambda$, we have that

$$\begin{aligned} \frac{ \beta +\alpha +\lambda }{2(1-\beta ) } = \frac{\beta (1-\alpha -\lambda )+\alpha +\lambda -(\lambda -\alpha )^2}{2(1-\beta )^2} =\sigma _0>0. \end{aligned}$$

We obtain a heat equation with a boundary condition $\textbf{e}(t,x=0)=s_0(t)$ and initial condition $\textbf{e}(t=0,x)=h(x)$. Upon denoting

$$\begin{aligned} {\mathcal {S}}(t,x,y):=\frac{1}{\sqrt{4\pi \delta \sigma _0 t}}\left( e^{-\frac{(x-y)^2}{4\delta \sigma _0 t}}-e^{-\frac{(x+y)^2}{4\delta \sigma _0 t}}\right) , \end{aligned}$$

the solution of the equation is given by

$$\begin{aligned} \textbf{e}(t,x){} & {} =s_0(t)+\int _0^{+\infty }{\mathcal {S}}(t,x,y)\left( h(y)-s_0(0)\right) \textrm{d}y\\{} & {} \quad -\int _0^t \int _0^{+\infty }{\mathcal {S}}(t-s,x,y)s_0'(s)\textrm{d}y\textrm{d}s, \quad t>0, \quad x>0, \end{aligned}$$

Let us remark that when $s_0(t)=s_0\in {\mathbb {R}}$ is constant for all $t\ge 0$, the above expression simplifies to

$$\begin{aligned} \textbf{e}(t,x)=s_0\left( 1-\textrm{erf}\left( \frac{x}{\sqrt{4\delta \sigma _0 t}} \right) \right) +\int _0^{+\infty }{\mathcal {S}}(t,x,y)h(y)\textrm{d}y, \quad t>0, \quad x>0. \end{aligned}$$

In conclusion, this section extended our discrete model towards a continuous limit in both space and time. In the temporal domain, it allowed us to understand our stable solution as an advection behavior, and alerted us that the other apparently oscillatory solutions previously observed in specific cases were mainly due to our discretization approximation. In the spatio-temporal domain, the continuous limit (21) allowed us to realize that our main Eq. (7) was merely a discrete version of a transport equation.

In the following sections, we will systematically return to discrete implementations (with gradually increasing functionality), before considering, again, their continuous formulations.

## Beyond the Identity Case

In the previous section we have studied in depth the case where ${\mathcal {W}}^f$ and ${\mathcal {W}}^b$ are both the identity matrix: each neuron in any given layer directly conveys its activation value to a single corresponding neuron in the next layer, and to a single neuron in the previous layer. Motivated by concrete implementations of the model in deep neural networks (Wen et al. 2018; Choksi et al. 2021), we aim to investigate more realistic situations with more complex connectivity matrices. While the generic unconstrained case (i.e., two unrelated and dense connection matrices ${\mathcal {W}}^f$ and ${\mathcal {W}}^b$) does not easily lend itself to analytical study, we will consider here two situations of practical interest: in the first one, the forward and backward connection matrices are symmetric and identical; in the second case, each matrix is symmetric, but the two are not necessarily identical.

### The Symmetric Rao and Ballard Case

Following the pioneering work of Rao and Ballard (1999), we will assume in this section that ${\mathcal {W}}^f=({\mathcal {W}}^b)^\textbf{t}$ and ${\mathcal {W}}^f$ is symmetric, which implies that

$$\begin{aligned} {\mathcal {W}}^f={\mathcal {W}}^b\in {\mathscr {S}}_d({\mathbb {R}}), \end{aligned}$$

where we denoted ${\mathscr {S}}_d({\mathbb {R}})$ the set of symmetric matrices on ${\mathbb {R}}^d$.

The underlying interpretation is that, if a strong synaptic connection exists from neuron *a* to neuron *b*, then there is also a strong connection from *b* to *a*. This assumption, which can find a possible justification from Hebbian plasticity rules (“neurons that fire together wire together”), does not capture all of the diversity of possible connectivity patterns, but it can be considered a good first approximation and has already been used in many computational studies, notably in the context of predictive coding models (Rao and Ballard 1999). Although this symmetry hypothesis is not biologically plausible, this setting is still very instructive from the mathematical point of view, since one can still manage to get a complete and exhaustive study as in the identity case of the previous section.

#### Neural Basis Change and Neural Assemblies

The spectral theorem ensures that ${\mathcal {W}}^f$ (and thus ${\mathcal {W}}^b$) is diagonalizable in an orthonormal basis. Namely, there exists an orthogonal invertible matrix $P\in {\mathscr {M}}_d({\mathbb {R}})$ such that $PP^\textbf{t}=P^\textbf{t}P={\textbf{I}}_d$ and there exists a diagonal matrix denoted ${\mathcal {D}}\in {\mathscr {M}}_d({\mathbb {R}})$ such that

$$\begin{aligned} {\mathcal {W}}^f={\mathcal {W}}^b=P {\mathcal {D}}P^\textbf{t}. \end{aligned}$$

We denote by $\gamma _p\in {\mathbb {R}}^d$, $p=1,\ldots ,d$ the diagonal elements of ${\mathcal {D}}$ and without loss of generality we may assume that

$$\begin{aligned} \gamma _1\le \cdots \le \gamma _d. \end{aligned}$$

Thanks to this diagonalization, we can now perform a change of basis for our neuronal space. We set ${\mathcal {U}}_j^n:= P^t{\mathcal {E}}_j^n$ as the new basis, with $P{\mathcal {U}}_j^n:= {\mathcal {E}}_j^n$. Each ${\mathcal {U}}_j^n$ can now be understood as a neural *assembly*, reflecting one of the principal components of the weight matrix ${\mathcal {W}}^f={\mathcal {W}}^b$. Importantly, although assemblies may overlap, activity updates induced by feedforward or feedback connections to one given assembly do not affect the other assemblies, since the matrix *P* is orthogonal. Therefore, our problem is much simplified when considering activity update equations at the level of these neural assemblies ${\mathcal {U}}_j^n$ rather than across individual neurons ${\mathcal {E}}_j^n$. Our model (1) becomes

$$\begin{aligned} {\mathcal {U}}_j^{n+1}=\beta {\mathcal {D}}{\mathcal {U}}_{j-1}^{n+1} +\alpha {\mathcal {D}}{\mathcal {U}}_{j-1}^n+\left[ (1-\beta -\lambda ){\textbf{I}}_d-\alpha {\mathcal {D}}^2 \right] {\mathcal {U}}_j^{n} + \lambda {\mathcal {D}}{\mathcal {U}}_{j+1}^{n}. \end{aligned}$$

Note that, because all matrices in the above equation are diagonal, we have totally decoupled the *d* components of the vector ${\mathcal {U}}_j^n$. More precisely, by denoting $u_{j,p}^{n}$ the *p*th component of ${\mathcal {U}}_j^n$, that is ${\mathcal {U}}_j^n=(u_{j,1}^{n},\ldots ,u_{j,d}^{n})^{\textbf{t}}$, we obtain

$$\begin{aligned}{} & {} u_{j,p}^{n+1}-\beta \gamma _p u_{j-1,p}^{n+1} =\alpha \gamma _p u_{j-1,p}^n +(1-\beta -\lambda -\alpha \gamma _p^2) u_{j,p}^{n} +\lambda \gamma _p u_{j+1,p}^{n}, \\{} & {} \quad \quad p=1,\ldots ,d. \end{aligned}$$

This indicates that one needs to study

23 $$\begin{aligned} u_{j}^{n+1}-\beta \gamma u_{j-1}^{n+1} =\alpha \gamma u_{j-1}^n +(1-\beta -\lambda -\alpha \gamma ^2) u_{j}^{n} +\lambda \gamma u_{j+1}^{n}, \end{aligned}$$

where $\gamma \in {\mathbb {R}}$ is a given parameter. Here, $\gamma$ can be thought of as the connection strength across layers (both feedforward and feedback, since we assumed here symmetric connectivity) of the neural assembly under consideration. By construction, each assembly in a given layer is only connected to the corresponding assembly in the layer above, and similarly in the layer below. Note that when $\gamma =1$, we encounter again the exact situation that we studied in the previous section (15), but now with neural assemblies in lieu of individual neurons.

#### Study of the Amplification Factor Function

Based on our previous analysis, the behavior of the solutions to (23) are intrinsically linked to the properties of the amplification factor function:

$$\begin{aligned} \rho _\gamma (\theta ) = \frac{\alpha \gamma \left( e^{-{\textbf{i}}\theta }-\gamma \right) +1-\beta +\lambda \left( \gamma e^{{\textbf{i}}\theta }-1\right) }{1-\beta \gamma e^{-{\textbf{i}}\theta }}, \quad \theta \in [-\pi ,\pi ], \end{aligned}$$

where one needs to ensure that $|\rho _\gamma (\theta )|\le 1$ for all $\theta \in [-\pi ,\pi ]$. The very first condition is to ensure that $1\ne \beta |\gamma |$ (to avoid division by zero). Next, we investigate the behavior of $\rho _\gamma (\theta )$ at $\theta =0$ and check under which condition on $\gamma$ we can ensure that $-1\le \rho _\gamma (0)\le 1$. We have

$$\begin{aligned} \rho _\gamma (0)=1+\frac{(1-\gamma )(\alpha \gamma -\lambda -\beta )}{1-\beta \gamma }, \end{aligned}$$

which readily tells us that $\rho _\gamma (0)=1$ if and only if $\gamma =1$ or $\gamma =\frac{\lambda +\beta }{\alpha }$. And on the other hand $\rho _\gamma (0)=-1$ if and only if $\gamma =\gamma _\pm ^0$ with

$$\begin{aligned} \gamma _\pm ^0=\frac{\lambda +\alpha -\beta \pm \sqrt{(\lambda +\alpha -\beta )^2+4\alpha (2-\lambda -\beta )}}{2\alpha }, \end{aligned}$$

with $\gamma _-^0<0<\gamma _+^0$ since $\lambda +\beta <2$ by assumption. One also notices that

$$\begin{aligned} (\lambda +\alpha -\beta )^2+4\alpha (2-\lambda -\beta )=(\lambda +3\alpha -\beta )^2+8\alpha (1-\alpha -\lambda ), \end{aligned}$$

such that either $\alpha +\lambda =1$ and in that case $\gamma _-^0=-1$ and $\gamma _+^0=1+\frac{1-\beta }{\alpha }>1$, or $\alpha +\lambda <1$ and in that case $\gamma _-^0<-1$ and $\gamma _+^0>1+\frac{1-\beta }{\alpha }>1$. Next, we remark that

$$\begin{aligned} \rho _\gamma (\pi )=1-\frac{(1+\gamma )(\alpha \gamma +\lambda +\beta )}{1+\beta \gamma }=\rho _{-\gamma }(0), \end{aligned}$$

which then implies that $\rho _\gamma (\pi )=1$ if and only if $\gamma =-1$ or $\gamma =-\frac{\lambda +\beta }{\alpha }$ and $\rho _\gamma (\pi )=-1$ if and only if $\gamma =-\gamma _\pm ^0$.

As explained in the beginning, our aim is to completely characterize under which conditions on $\gamma \in {\mathbb {R}}$, $0\le \beta <1$ and $0<\alpha ,\lambda <1$ with $\alpha +\lambda \le 1$, one can ensure that $|\rho _\gamma (\theta )|\le 1$ for all $\theta \in [-\pi ,\pi ]$.

Potential regions of marginal stability are thus given by those values of the parameters satisfying $\gamma =\pm 1$, $\gamma =\pm \frac{\lambda +\beta }{\alpha }$, $\gamma =\pm \gamma _+^0$ and $\gamma =\pm \gamma _-^0$, and it is important to determine the intersections among the above regions. We have already proved that $\gamma _-^0=-1$ whenever $\alpha =1-\lambda$. Next, we compute that $\gamma _-^0=-\frac{\lambda +\beta }{\alpha }$ whenever $\alpha =\frac{\lambda (\lambda +\beta )}{1-\lambda -\beta }=:\Lambda$, while $\gamma _-^0=-\gamma _+^0$ whenever $\alpha =\beta -\lambda$ and $\gamma _+^0=\frac{\lambda +\beta }{\alpha }$ when $\alpha =\beta (\lambda +\beta )$. Let us already point out that $\Lambda$ is only defined if $\lambda +\beta <1$ and in that case $\Lambda >0$.

**Fig. 11:**
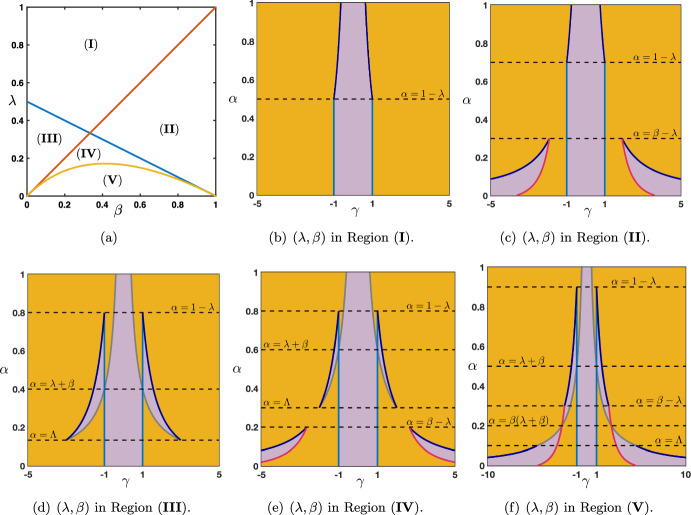
**a** Geometry of the five stability regions in the $(\beta ,\lambda )$ plane. The blue curve is given by $2\lambda +\beta =1$, the orange curve by $\lambda =\beta$ while the yellow parabola is parametrized by $\beta ^2-\beta (1-\lambda )+\lambda =0$. **b**–**f** Stability/instability regions and their boundaries as a function of $(\alpha ,\gamma )$ for (23) while $(\lambda ,\beta )$ being fixed in one the five regions given in panel (**a**). Shaded orange regions correspond to an instability for (23) while purple regions correspond to a stability for (23). The boundaries of the stability/instability regions are given by the intersections of the parametrized curves $\gamma =\pm 1$ (blue curves), $\gamma =\pm \gamma _-^0$ (dark blue curves), $\gamma =\pm \gamma _+^0$ (pink curves) and $\gamma =\pm \frac{\lambda +\beta }{\alpha }$ (gray curves). Note that the region of interest is $0<\alpha \le 1-\lambda$. Along each such parametrized curves Eq.  (23) is marginally stable (Color figure online)

We now introduce five regions in the quadrant $(\beta ,\lambda )\in [0,1)\times (0,1)$ which are depicted in Fig. 11a. First, since $1-\lambda =\Lambda =\lambda +\beta$ if and only if $2\lambda +\beta =1$ (which corresponds to the blue line in Fig. 11a), we deduce that when $2\lambda +\beta \ge 1$ we have $1-\lambda \le \min (\Lambda ,\lambda +\beta )$ which leads us to define the following two regions:

$$\begin{aligned} \mathbf {(I)}&:=\left\{ (\beta ,\lambda )\in [0,1)\times (0,1) ~|~ 2\lambda +\beta \ge 1 \text { and } \beta \le \lambda \right\} , \\ \mathbf {(II)}&:=\left\{ (\beta ,\lambda )\in [0,1)\times (0,1) ~|~ 2\lambda +\beta \ge 1 \text { and } \beta >\lambda \right\} . \end{aligned}$$

Now, when $2\lambda +\beta <1$, we have the strict ordering $0<\Lambda<\lambda +\beta <1-\lambda$ and when $\beta >\lambda$ it is thus necessary to compare $\Lambda$ to $\beta -\lambda$. We remark that $\Lambda =\beta -\lambda =\beta (\lambda +\beta )$ if and only if $\beta ^2-\beta (1-\lambda )+\lambda =0$, which corresponds to the yellow parabola in Fig. 11a. We thus define the following three regions:

$$\begin{aligned} \mathbf {(III)}&:=\left\{ (\beta ,\lambda )\in [0,1)\times (0,1) ~|~ 2\lambda +\beta<1 \text { and } \beta \le \lambda \right\} , \\ \mathbf {(IV)}&:=\left\{ (\beta ,\lambda )\in [0,1)\times (0,1) ~|~ 2\lambda +\beta<1, \beta>\lambda \text { and } \beta ^2-\beta (1-\lambda )+\lambda \ge 0 \right\} ,\\ \mathbf {(V)}&:=\left\{ (\beta ,\lambda )\in [0,1)\times (0,1) ~|~ 2\lambda +\beta<1, \beta >\lambda \text { and } \beta ^2-\beta (1-\lambda )+\lambda <0\right\} . \end{aligned}$$

Note that when $(\beta ,\lambda )$ is in region $\mathbf {(IV)}$, we have the ordering

$$\begin{aligned} 0<\beta -\alpha \le \Lambda<\lambda +\beta <1-\lambda , \end{aligned}$$

while for $(\beta ,\lambda )$ in region $\mathbf {(V)}$, we have

$$\begin{aligned} 0<\Lambda<\beta (\lambda +\beta )<\beta -\alpha<\lambda +\beta <1-\lambda . \end{aligned}$$

We can characterize the stability of our equation separately for each of the five regions defined in Fig. 11a. Since the region already determines the value of the parameters $\beta$ and $\lambda$, the stability will be expressed as a function of the two remaining parameters $\alpha$ and $\gamma$ (Fig. 11b–f). We refer to Fig. 11b–f for a comprehensive representation of the stability regions. Note that the boundaries of the stability/instability regions are precisely given by the intersections of the parametrized curves $\gamma =\pm 1$ (blue curves), $\gamma =\pm \gamma _-^0$ (dark blue curves), $\gamma =\pm \gamma _+^0$ (pink curves) and $\gamma =\pm \frac{\lambda +\beta }{\alpha }$ (gray curves). Along each such parametrized curves Eq. (23) is marginally stable. We comment below the case $(\lambda ,\beta )$ in Region $\mathbf {(III)}$. The other cases can be described in the same way, but we leave this out for conciseness.

**Table 1:** Expressions of the wave speed $c_0^\gamma$ and $c_\pi ^\gamma$ for values of $\gamma$ corresponding the boundaries of the stability regions from Fig. 11

	$\gamma =1$	$\gamma =-1$	$\gamma =\frac{\lambda +\beta }{\alpha }$	$\gamma =-\frac{\lambda +\beta }{\alpha }$	$\gamma = \gamma _\pm ^0$	$\gamma =- \gamma _\pm ^0$
$c_0^\gamma$	$\frac{\beta +\alpha -\lambda }{1-\beta }$	$\frac{\alpha -\lambda -\beta }{1+\beta }$	$\frac{(\beta +\lambda )(\beta +\alpha -\lambda )}{\alpha -\beta (\lambda +\beta )}$		$\frac{\gamma _\pm ^0(\beta +\lambda -\alpha )}{1-\beta \gamma _\pm ^0}$	
$c_\pi ^\gamma$	$\frac{\alpha -\lambda -\beta }{1+\beta }$	$\frac{\beta +\alpha -\lambda }{1-\beta }$		$\frac{(\beta +\lambda )(\beta +\alpha -\lambda )}{\alpha -\beta (\lambda +\beta )}$		$\frac{\gamma _\pm ^0(\beta +\lambda -\alpha )}{1-\beta \gamma _\pm ^0}$

Let us note that $c_\pi ^1$ and $c_0^{-1}$ only exist when $\alpha +\lambda =1$

Suppose that $(\lambda ,\beta )$ belongs to Region $\mathbf {(III)}$. We present the results of Fig. 11d by letting $\alpha$ vary between 0 and $1-\lambda$ and $\gamma \in {\mathbb {R}}$. More precisely, for each fixed $\alpha \in (0,1-\lambda )$ we investigate the stability properties as a function of $\gamma$. We have to distinguish between several subcases.

(i) If $0<\alpha <\Lambda$. Then, Eq. (23) is stable for each $\gamma \in (-1,1)$, unstable for $|\gamma |>1$ and marginally stable at $\gamma =\pm 1$ with $\rho _1(0)=1$ and $\rho _{-1}(\pm \pi )=1$.
(ii) If $\alpha =\Lambda$. Then $\gamma _-^0=-\frac{\lambda +\beta }{\alpha }$ and Eq. (23) is stable for each $\gamma \in (-1,1)$, unstable for $|\gamma |>|\gamma _-^0|$ and $|\gamma _-^0|>|\gamma |>1$, whereas it is marginally stable at $\gamma =\pm 1$ and at $\gamma =\pm \gamma _-^0$ with $\rho _1(0)=1$, $\rho _{-1}(\pm \pi )=1$, $\rho _{\gamma _-^0}(0)=-1$ and $\rho _{-\gamma _-^0}(\pm \pi )=-1$ together with $\rho _{-\gamma _-^0}(0)=1$, $\rho _{\gamma _-^0}(\pm \pi )=1$.
(iii) If $\Lambda<\alpha <\lambda +\beta$. Then, Eq. (23) is stable for each $\gamma \in (-1,1)$ and $\frac{\lambda +\beta }{\alpha }<|\gamma |<|\gamma _-^0|$, unstable for $|\gamma |>|\gamma _-^0|$ and $\frac{\lambda +\beta }{\alpha }>|\gamma |>1$ and marginally stable at $\gamma \in \left\{ \pm 1,\pm \frac{\lambda +\beta }{\alpha },\pm \gamma _-^0\right\}$ with $\rho _1(0)=1$, $\rho _{-1}(\pm \pi )=1$, $\rho _{\frac{\lambda +\beta }{\alpha }}(0)=1$, $\rho _{-\frac{\lambda +\beta }{\alpha }}(\pm \pi )=1$, $\rho _{\gamma _-^0}(0)=-1$ and $\rho _{-\gamma _-^0}(\pm \pi )=-1$.
(iv) If $\alpha =\lambda +\beta$. Then, Eq. (23) is stable for each $\gamma \in (\gamma _-^0,-\gamma _-^0)\backslash \{\pm 1\}$, unstable for $|\gamma |>|\gamma _-^0|$ and marginally stable at $\gamma \in \left\{ \pm 1,\pm \gamma _-^0\right\}$ with $\rho _1(0)=1$, $\rho _{-1}(\pm \pi )=1$, $\rho _{\gamma _-^0}(0)=-1$ and $\rho _{-\gamma _-^0}(\pm \pi )=-1$. Remark that in this case we have $\frac{\lambda +\beta }{\alpha }=1$.
(v) If $\lambda +\beta<\alpha <1-\lambda$. Then, Eq. (23) is stable for each $\gamma \in \left( -\frac{\lambda +\beta }{\alpha },\frac{\lambda +\beta }{\alpha }\right)$ and $1<|\gamma |<|\gamma _-^0|$, unstable for $|\gamma |>|\gamma _-^0|$ and $1>|\gamma |>\frac{\lambda +\beta }{\alpha }$ and marginally stable at $\gamma \in \left\{ \pm 1,\pm \frac{\lambda +\beta }{\alpha },\pm \gamma _-^0\right\}$ with $\rho _1(0)=1$, $\rho _{-1}(\pm \pi )=1$, $\rho _{\frac{\lambda +\beta }{\alpha }}(0)=1$, $\rho _{-\frac{\lambda +\beta }{\alpha }}(\pm \pi )=1$, $\rho _{\gamma _-^0}(0)=-1$ and $\rho _{-\gamma _-^0}(\pm \pi )=-1$.
(vi) If $\alpha =1-\lambda$. Then, Eq. (23) is stable for each $\gamma \in \left( -\frac{\lambda +\beta }{\alpha },\frac{\lambda +\beta }{\alpha }\right)$, unstable for $|\gamma |>|\gamma _-^0|=1$ and $1>|\gamma |>\frac{\lambda +\beta }{\alpha }$ and marginally stable at $\gamma \in \left\{ \pm 1,\pm \frac{\lambda +\beta }{\alpha }\right\}$ with $\rho _1(0)=1$ and $\rho _{1}(\pm \pi )=-1$, $\rho _{-1}(\pm \pi )=1$ and $\rho _{-1}(0)=-1$, with $\rho _{\frac{\lambda +\beta }{\alpha }}(0)=1$, $\rho _{-\frac{\lambda +\beta }{\alpha }}(\pm \pi )=1$. Remark that in this case we have $\gamma _-^0=-1$.

**Summary** In summary, we see that stability is nearly guaranteed whenever $-1<\gamma <1$, regardless of the values of other parameters (as long as $0<\alpha <1-\lambda$). This makes intuitive sense, as $\gamma$ represents the connection strength across layers of a particular neural assembly, a connection weight $|\gamma |<1$ implies that activity of this assembly will remain bounded across layers. Additionally, and perhaps more interestingly, in some but not all regions (e.g., Regions II and V) stability can be obtained for much larger values of $|\gamma |$; this, however, appears to coincide with low values of the $\alpha$ parameter. In other words, for high connection strengths $|\gamma |$, the feedforward error correction term $\alpha$ makes the system unstable.

#### Wave Speed Characterization

**Fig. 12:**
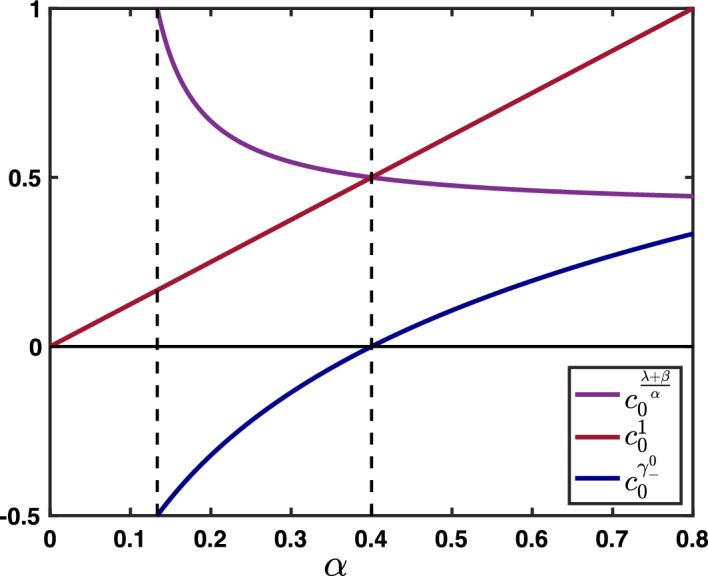
Plot of the wave speeds $c_0^1$, $c_0^{\frac{\lambda +\beta }{\alpha }}$ and $c_0^{\gamma _-^0}$ as a function of $\alpha$ in the case $(\lambda ,\beta )$ in Region $\textbf{III}$ associated to Fig. 11d (Color figure online)

In the previous section (The Identity Case), we have proved that the direction of propagation was given by the sign of $c_0$ and $c_\pi$ whenever they exist which could be read off from the behavior of $\rho _\gamma (\theta )$ near $\theta =0$ or $\theta =\pm \pi$. We have reported the values of $c_0^\gamma$ and $c_{\pi }^\gamma$ for different values of $\gamma$ in Table 1. For example, in Fig. 12 we illustrate the changes in propagation speed and direction for $c_0^\gamma$ for the case $(\lambda ,\beta )$ in Region $\mathbf {(III)}$ (as defined in Fig. 11a), but the calculations remain valid for the other regions.

It is worth emphasizing that for fixed values of the hyper-parameters $\alpha$, $\beta$ and $\lambda$, we see here that varying $\gamma$ can give rise to different propagation speeds or even different directions. As each neuronal assembly $u_{j,p}$ in a given layer *j* is associated with its own connection strength $\gamma _p$, it follows that different speeds and even different directions of propagation can concurrently be obtained in a single network, one for each assembly. For instance, in a given network with hyperparameters $\alpha =0.2$, $\beta =0.2$ and $\lambda =0.3$ (region $\textbf{III}$), a neural assembly with a connection strength of $\gamma =1$ would propagate forward at a relatively slow speed, while another with $\gamma =2.5$ would propagate in the same direction at a much faster speed, and yet another assembly with $\gamma =\gamma ^0_-\approx -2.09$ would simultaneously propagate in the opposite backward direction.

#### Continuous in Time Interpretation

We can repeat the “continuous system” analysis conducted in the previous section (The Identity Case), which has lead to (20), but this time with Rao-Ballard connection matrices between layers. With the same scaling on the hyperparameters

$$\begin{aligned} {\widetilde{\beta }}:=\frac{\beta }{\Delta t}, \quad {\widetilde{\lambda }}:=\frac{\lambda }{\Delta t}, \text { and } {\widetilde{\alpha }}:=\frac{\alpha }{\Delta t}, \end{aligned}$$

we get that, at the limit $\Delta t\rightarrow 0$, the Eq. (23) becomes the following lattice ordinary differential equation

24 $$\begin{aligned} \frac{\textrm{d}}{\textrm{d}t}\textbf{u}_j(t)=({\widetilde{\beta }}+{\widetilde{\alpha }})\gamma \textbf{u}_{j-1}(t)-({\widetilde{\beta }}+{\widetilde{\lambda }}+{\widetilde{\alpha }}\gamma ^2)\textbf{u}_j(t)+{\widetilde{\lambda }}\gamma \textbf{u}_{j+1}(t), \quad t>0.\nonumber \\ \end{aligned}$$

Note that the neuronal layer activity is now expressed in terms of neural assemblies $\textbf{u}_j$ rather than individual neurons $\textbf{e}_j$.

The amplification factor function in this case is given by

$$\begin{aligned} \nu _\gamma (\theta )=({\widetilde{\beta }}+{\widetilde{\alpha }})\gamma e^{-{\textbf{i}}\theta }-({\widetilde{\beta }}+{\widetilde{\lambda }}+{\widetilde{\alpha }}\gamma ^2)+{\widetilde{\lambda }}\gamma e^{{\textbf{i}}\theta }, \quad \theta \in [-\pi ,\pi ], \end{aligned}$$

whose real part is given by

$$\begin{aligned} \textrm{Re}(\nu _\gamma (\theta ))=({\widetilde{\beta }}+{\widetilde{\alpha }}+{\widetilde{\lambda }})\gamma \cos (\theta )-({\widetilde{\beta }}+{\widetilde{\lambda }}+{\widetilde{\alpha }}\gamma ^2), \quad \theta \in [-\pi ,\pi ]. \end{aligned}$$

When $\gamma >0$, we observe that

$$\begin{aligned} \underset{\theta \in [-\pi ,\pi ]}{\max }\textrm{Re}(\nu _\gamma (\theta ))=\textrm{Re}(\nu _\gamma (0))=({\widetilde{\lambda }}+{\widetilde{\beta }}-{\widetilde{\alpha }}\gamma )(\gamma -1), \end{aligned}$$

whereas when $\gamma <0$, we have

$$\begin{aligned} \underset{\theta \in [-\pi ,\pi ]}{\max }\textrm{Re}(\nu _\gamma (\theta ))=\textrm{Re}(\nu _\gamma (\pm \pi ))=-({\widetilde{\lambda }}+{\widetilde{\beta }}+{\widetilde{\alpha }}\gamma )(\gamma +1). \end{aligned}$$

**Fig. 13:**
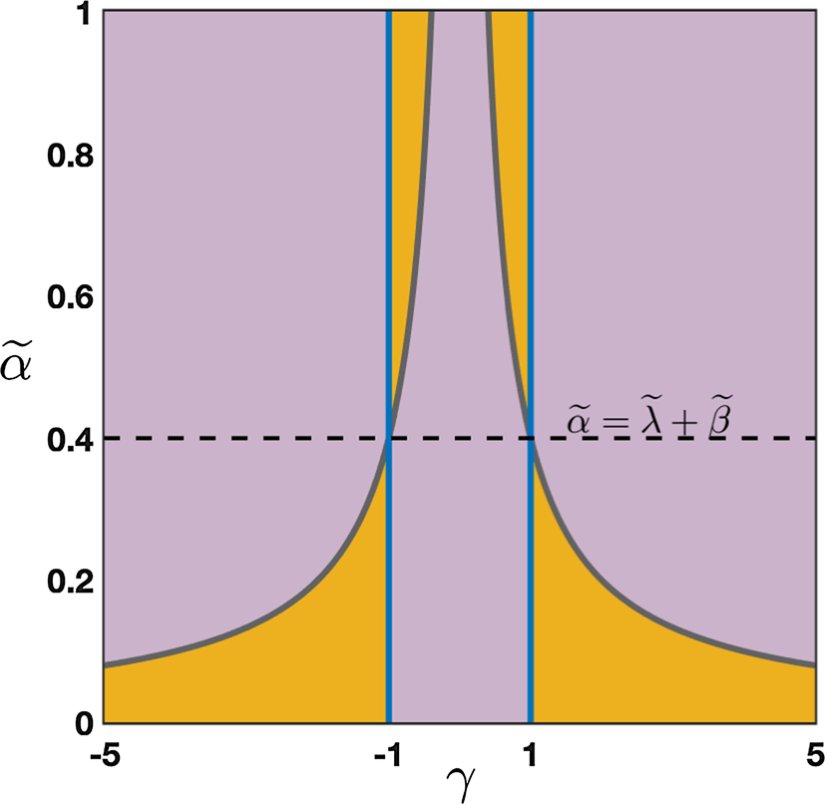
Stability/instability regions and their boundaries as a function of $({\widetilde{\alpha }},\gamma )$ for (24) for any $({\widetilde{\lambda }},{\widetilde{\beta }})$ fixed. The shaded orange region corresponds to an instability for (24) while the purple region corresponds to a stability for (24). The boundaries of the stability/instability regions are given by the intersections of the parametrized curves $\gamma =\pm 1$ (blue curves) and $\gamma =\pm \frac{\lambda +\beta }{\alpha }$ (gray curves) where Eq. (24) is marginally stable (color figure online)

As a consequence, the stability analysis in this case is very simple and depends only on the relative position of $\gamma$ with respect to $\pm 1$ and $\pm \frac{{\widetilde{\lambda }}+{\widetilde{\beta }}}{{\widetilde{\alpha }}}$. It is summarized in Fig. 13.

The simple behavior illustrated in Fig. 13 for our continuous system contrasts with the number and diversity of behaviors obtained for the discrete version of the same system (Fig. 11). A number of points are worth highlighting. For instance, although the values of $\beta$ and $\lambda$ were critical for the discrete system (to define the region (I)–(V)), they do not affect the qualitative behavior of the continuous system. Furthermore, some observations in the continuous system appear to contradict the conclusions made previously in the discrete case. We see that stability can still be obtained with high values of the connection weight $\gamma>>1$, but this time the stable regions coincide with high $\alpha$ values, whereas it was the opposite in Fig. 11 panels (b, f). This qualitative difference in behavior can be taken as a point of caution, to remind us that a discrete approximation of the system can be associated with important errors in interpretation.

Finally we note that, while stability regions are qualitatively different in the continuous case compared to the discrete approximation, the speed and direction of propagation of neural signals (reflected in the variables $c_0$ and $c_{\pi }$ when they exist) remains comparable.

#### A Class of Examples

In this section, we provide a class of examples of ${\mathcal {W}}^f$ amenable to a complete analysis. Namely we consider ${\mathcal {W}}^f$ as the following linear combination

25 $$\begin{aligned} {\mathcal {W}}^f=\zeta {\textbf{I}}_d+\xi {\textbf{A}}, \end{aligned}$$

for some $\zeta ,\xi \in {\mathbb {R}}$ where ${\textbf{A}}\in {\mathscr {M}}_d({\mathbb {R}})$ is given by

$$\begin{aligned} {\textbf{A}}=\left( \begin{matrix} -2 &{} 1 &{} 0 &{} \cdots &{} \cdots &{} 0 \\ 1 &{} -2 &{} 1 &{} \ddots &{} \ddots &{} \vdots \\ 0 &{} \ddots &{} \ddots &{} \ddots &{} \ddots &{} \vdots \\ \vdots &{} \ddots &{} \ddots &{} \ddots &{} \ddots &{} \vdots \\ \vdots &{} \ddots &{} \ddots &{} 1 &{} -2 &{} 1 \\ 0 &{} \cdots &{} \cdots &{} 0 &{} 1 &{} -2 \end{matrix} \right) . \end{aligned}$$

The matrix ${\textbf{A}}$ is nothing but the discrete laplacian and ${\mathcal {W}}^f$ acts as a convolution operator on ${\mathbb {R}}^d$. More precisely, ${\mathcal {W}}^f$ combines a convolution term with a residual connection term, as in the well-known ResNet architecture (He et al. 2016). Let us also note that the spectrum of ${\textbf{A}}$ is well known and given by

$$\begin{aligned} \textrm{Spec}({\textbf{A}})=\left\{ -4\sin ^2\left( \frac{p\pi }{2(d+1)}\right) , \quad p=1,\ldots ,d\right\} . \end{aligned}$$

As a consequence, the spectrum of ${\mathcal {W}}^f$ is simply given by

$$\begin{aligned} \textrm{Spec}(\mathbf {{\mathcal {W}}^f})=\left\{ \zeta -4\xi \sin ^2\left( \frac{p\pi }{2(d+1)}\right) , \quad p=1,\ldots ,d\right\} . \end{aligned}$$

One can for example set

$$\begin{aligned} \zeta =\frac{\sin ^2\left( \frac{d\pi }{2(d+1)}\right) +\sin ^2\left( \frac{\pi }{2(d+1)}\right) }{\sin ^2\left( \frac{d\pi }{2(d+1)}\right) -\sin ^2\left( \frac{\pi }{2(d+1)}\right) }\quad \text { and } \quad \xi =\frac{1}{2\left( \sin ^2\left( \frac{d\pi }{2(d+1)}\right) -\sin ^2\left( \frac{\pi }{2(d+1)}\right) \right) }, \end{aligned}$$

such that

$$\begin{aligned} \zeta -4\xi \sin ^2\left( \frac{d\pi }{2(d+1)}\right) =-1, \quad \zeta -4\xi \sin ^2\left( \frac{\pi }{2(d+1)}\right) =1, \end{aligned}$$

and for all $p=2,\ldots ,d-1$

$$\begin{aligned} \zeta -4\xi \sin ^2\left( \frac{p\pi }{2(d+1)}\right) \in (-1,1). \end{aligned}$$

Next, for any $p=1,\ldots ,d$ the eigenvector corresponding to the eigenvalue $-4\sin ^2\left( \frac{p\pi }{2(d+1)}\right)$ is

$$\begin{aligned} U_p=\left( \cos \left( \frac{p\pi }{d+1}\right) ,\ldots ,\cos \left( \frac{pk\pi }{d+1}\right) ,\ldots ,\cos \left( \frac{pd\pi }{d+1}\right) \right) ^\textbf{t}\in {\mathbb {R}}^d. \end{aligned}$$

$U_p$ is the projection vector that corresponds to the $p^{th}$ neural assembly $u_{j,p}$ as defined above.

Along $U_1$, the recurrence equation reduces to (23) with $\gamma =1$, while along $U_d$, the recurrence equation reduces to (23) with $\gamma =-1$, and we can apply the results of the previous section (the Identity case). In between (for all $1\le p\le d$) we see that the eigenvalues of our connection matrix $\mathbf {{\mathcal {W}}^f}$ span the entire range between $-1$ and 1, that they can be explicitly computed, and thus that the stability, propagation speed and direction of activity in the corresponding neural assembly can be determined.

#### Fully Continuous Interpretation in Time, Depth and Width

For the same class of example (connection matrix composed of a convolution and residual terms), we now wish to provide a fully continuous interpretation for model (1) in the special case $\zeta =1$ and $\xi$ adjusted as follows. By fully continuous, we mean that we explore the limit of our model when not only time *t*, but also network depth *x* *and* neuronal layer width *y* are considered as continuous variables. Although we already presented a model that was continuous in both *time* and *depth* in Sect. 3.3.2, the layers in that model only comprised a single neuron, and had no intrinsic spatial dimension. We now introduce this third continuous dimension. The starting point is to see ${\mathcal {E}}_{j,k}^n$, the *k*th element of ${\mathcal {E}}_{j}^n$, as an approximations of some continuous function ${\mathcal {E}}(t,x,y)$ evaluated at $t_n=n\Delta t$, $x_j=j\Delta x$ and $y_k=k\Delta y$ for some $\Delta t>0$, $\Delta x>0$ and $\Delta y>0$. Let us first remark that the action of ${\textbf{A}}$ on ${\mathcal {E}}_j^n$ is given by

$$\begin{aligned} ({\textbf{A}}{\mathcal {E}}_j^n)_k={\mathcal {E}}_{j,k-1}^n-2{\mathcal {E}}_{j,k}^n+{\mathcal {E}}_{j,k+1}^n, \end{aligned}$$

which can be seen at a discrete approximation of $\partial _y^2 {\mathcal {E}}(t_n,x_j,y_k)$ up to a scaling factor of order $\Delta y^2$. Once again, setting $\nu =\frac{\Delta x}{\Delta t}$ and introducing $\kappa =\frac{\Delta y^2}{\Delta t}$, we may rewrite (1) with ${\mathcal {W}}^f={\mathcal {W}}^b={\textbf{I}}_d+\xi {\textbf{A}}$ as

$$\begin{aligned} (1-\beta )\frac{{\mathcal {E}}_j^{n+1}-{\mathcal {E}}_j^n}{\Delta t}&=\beta \nu \frac{{\mathcal {E}}_{j-1}^{n+1} -{\mathcal {E}}_j^{n+1}}{\Delta x}+ \lambda \nu \frac{ {\mathcal {E}}_{j+1}^{n}-{\mathcal {E}}_j^{n}}{\Delta x}-\alpha \nu \frac{ {\mathcal {E}}_{j}^{n}-{\mathcal {E}}_{j-1}^{n}}{\Delta x}\\&~~~+\beta \xi \kappa \frac{{\textbf{A}}}{\Delta y^2}{\mathcal {E}}_{j-1}^{n+1}+\alpha \xi \kappa \frac{{\textbf{A}}}{\Delta y^2}{\mathcal {E}}_{j-1}^{n}-2\alpha \xi \kappa \frac{{\textbf{A}}}{\Delta y^2}{\mathcal {E}}_{j}^{n}\\&\quad -\alpha \xi ^2 \kappa \Delta y^2 \frac{{\textbf{A}}}{\Delta y^2} \frac{{\textbf{A}}}{\Delta y^2}{\mathcal {E}}_{j}^{n} +\lambda \xi \kappa \frac{{\textbf{A}}}{\Delta y^2}{\mathcal {E}}_{j+1}^{n}. \end{aligned}$$

Now letting $\Delta t\rightarrow 0$, $\Delta x\rightarrow 0$ and $\Delta y\rightarrow 0$ with $\nu$ and $\kappa$ fixed, we obtain the following partial differential equation

$$\begin{aligned} \partial _t {\mathcal {E}}(t,x,y) + \frac{\nu (\beta +\alpha -\lambda )}{1-\beta } \partial _x {\mathcal {E}}(t,x,y) =\frac{\xi \kappa (\beta +\lambda -\alpha )}{1-\beta }\partial _y^2{\mathcal {E}}(t,x,y). \end{aligned}$$

This is a diffusion equation along the *y* dimension while being a transport equation in the *x* direction. As such, it is only well defined (or stable) when the sign of the diffusion coefficient in front of $\partial _y^2{\mathcal {E}}(t,x,y)$ is positive. This depends on the sign of $\xi$ and $\beta +\lambda -\alpha$, which need to verify $\xi (\beta +\lambda -\alpha )>0$. In that case, the system diffuses neural activity along the dimension *y* such that the entire neuronal layer converges to a single, uniform activation value when $t\rightarrow \infty$.

### The General Symmetric Case

Finally, we now wish to relax some of the assumptions made in the previous Rao-Ballard case. Thus, the last case that we present is one where we assume that

(i) ${\mathcal {W}}^f$ and ${\mathcal {W}}^b$ are symmetric matrices, that is ${\mathcal {W}}^f,{\mathcal {W}}^b\in {\mathscr {S}}_d({\mathbb {R}})$,
(ii) ${\mathcal {W}}^f$ and ${\mathcal {W}}^b$ commute, that is ${\mathcal {W}}^f{\mathcal {W}}^b={\mathcal {W}}^b{\mathcal {W}}^f$.

But we do not necessarily impose that ${\mathcal {W}}^f=({\mathcal {W}}^b)^\textbf{t}$ as in the Rao and Ballard’s previous case. Let us already note that examples of matrices verifying the above conditions are residual convolution matrices introduced in (25), that is ${\mathcal {W}}^f=\zeta _f {\textbf{I}}_d+\xi _f {\textbf{A}}$ and ${\mathcal {W}}^b=\zeta _b {\textbf{I}}_d+\xi _b {\textbf{A}}$ for some $\zeta _{b,f},\xi _{b,f}\in {\mathbb {R}}$. Under assumptions (i) and (ii), ${\mathcal {W}}^f$ and ${\mathcal {W}}^b$ can be diagonalized in the same orthonormal basis, meaning that there exist an invertible orthogonal matrix $P\in {\mathscr {M}}_d({\mathbb {R}})$ such that $PP^{\textbf{t}}=P^{\textbf{t}}P=I_d$, and two diagonal matrices ${\mathcal {D}}^f\in {\mathscr {M}}_d({\mathbb {R}})$ and ${\mathcal {D}}^b\in {\mathscr {M}}_d({\mathbb {R}})$ with the properties that

$$\begin{aligned} P^{\textbf{t}}{\mathcal {W}}^fP= {\mathcal {D}}^f, \quad \text { and } \quad P^{\textbf{t}}{\mathcal {W}}^bP= {\mathcal {D}}^b. \end{aligned}$$

For future reference, we denote by $\gamma _p^{f,b}$ for each $1\le p \le d$ the diagonal elements of ${\mathcal {D}}^{f,b}$. Once again, we can use the matrix *P* to apply an orthonormal basis change and create neural *asssemblies* ${\mathcal {U}}_j^n:=P^t{\mathcal {E}}_j^n$. With $P{\mathcal {U}}_j^n:={\mathcal {E}}_j^n$, the recurrence equation becomes

$$\begin{aligned} {\mathcal {U}}_j^{n+1}-\beta {\mathcal {D}}^f {\mathcal {U}}_{j-1}^{n+1} =\alpha {\mathcal {D}}^b {\mathcal {U}}_{j-1}^n+\left[ (1-\beta -\lambda ){\textbf{I}}_d-\alpha {\mathcal {D}}^b {\mathcal {D}}^b\right] {\mathcal {U}}_j^{n} + \lambda {\mathcal {D}}^b {\mathcal {U}}_{j+1}^{n}. \end{aligned}$$

Note that, because all matrices in the above equation are diagonal, we have also totally decoupled the *d* components of the vector ${\mathcal {U}}_j^n$. More precisely, by denoting $u_{j,p}^{n}$ the *p*th component of ${\mathcal {U}}_j^n$, that is ${\mathcal {U}}_j^n=(u_{j,1}^{n},\ldots ,u_{j,d}^{n})^{\textbf{t}}$, we obtain

$$\begin{aligned}{} & {} u_{j,p}^{n+1}-\beta \gamma _p^f u_{j-1,p}^{n+1} =\alpha \gamma _p^b u_{j-1,p}^n +(1-\beta -\lambda -\alpha \left( \gamma _p^b\right) ^2) u_{j,p}^{n} +\lambda \gamma _p^b u_{j+1,p}^{n}, \\{} & {} \quad p=1,\ldots ,d. \end{aligned}$$

This indicates that one needs to study

26 $$\begin{aligned} u_{j}^{n+1}-\beta \gamma _1 u_{j-1}^{n+1} =\alpha \gamma _2 u_{j-1}^n +(1-\beta -\lambda -\alpha \gamma _2^2) u_{j}^{n} +\lambda \gamma _2 u_{j+1}^{n}, \end{aligned}$$

where $\gamma _{1,2}\in {\mathbb {R}}$ are now two given parameters. As before, $\gamma _{1,2}$ can be thought of as the connection strength across layers of the neural assembly under consideration. By construction, each assembly in a given layer is only connected to the corresponding assembly in the layer above, and similarly in the layer below, with $\gamma _1$ for the feedforward direction and $\gamma _2$ for the feedback direction. Note that $\gamma _1=\gamma _2$ would then correspond to the Rao-Ballard situation studied previously.

#### Study of the Amplification Factor Function

Repeating the previous analysis, one needs to understand the amplification factor

$$\begin{aligned} \rho _{\gamma _1,\gamma _2}(\theta ) = \frac{\alpha \gamma _2 \left( e^{-{\textbf{i}}\theta }-\gamma _2\right) +1-\beta +\lambda \left( \gamma _2 e^{{\textbf{i}}\theta }-1\right) }{1-\beta \gamma _1 e^{-{\textbf{i}}\theta }}, \quad \theta \in [-\pi ,\pi ]. \end{aligned}$$

We already note a symmetry property of the amplification factor function which reads

$$\begin{aligned} \rho _{\gamma _1,\gamma _2}(\theta )=\rho _{-\gamma _1,-\gamma _2}(\theta \pm \pi ), \quad \theta \in [-\pi ,\pi ]. \end{aligned}$$

As a consequence, whenever $\rho _{\gamma _1,\gamma _2}(0)=\pm 1$ one has $\rho _{-\gamma _1,-\gamma _2}(\pm \pi )=\pm 1$ for the same values of the parameters. Then, we note that

$$\begin{aligned} \rho _{\gamma _1,\gamma _2}(0)=1 \Longleftrightarrow \gamma _1=\chi (\gamma _2), \end{aligned}$$

where the function $\chi (x)$, depending only on the hyper-parameters, is given by

27 $$\begin{aligned} \chi (x):=\frac{\alpha x^2-(\alpha +\lambda )x+\lambda +\beta }{\beta }, x\in {\mathbb {R}}. \end{aligned}$$

Thus, using the above symmetry, we readily deduce that

$$\begin{aligned} \rho _{\gamma _1,\gamma _2}(\pm \pi )=1 \Longleftrightarrow \gamma _1=-\chi (-\gamma _2). \end{aligned}$$

Finally, we compute that

$$\begin{aligned} \rho _{\gamma _1,\gamma _2}(0)=-1 \Longleftrightarrow \gamma _1=\zeta (\gamma _2), \end{aligned}$$

where the function $\zeta (x)$, depending only on the hyper-parameters, is given by

28 $$\begin{aligned} \zeta (x):=\frac{-\alpha x^2+(\alpha +\lambda )x+2-\lambda -\beta }{\beta }, x\in {\mathbb {R}}. \end{aligned}$$

Using the above symmetry, we readily deduce that

$$\begin{aligned} \rho _{\gamma _1,\gamma _2}(\pm \pi )=-1 \Longleftrightarrow \gamma _1=-\zeta (-\gamma _2). \end{aligned}$$

**Fig. 14:**
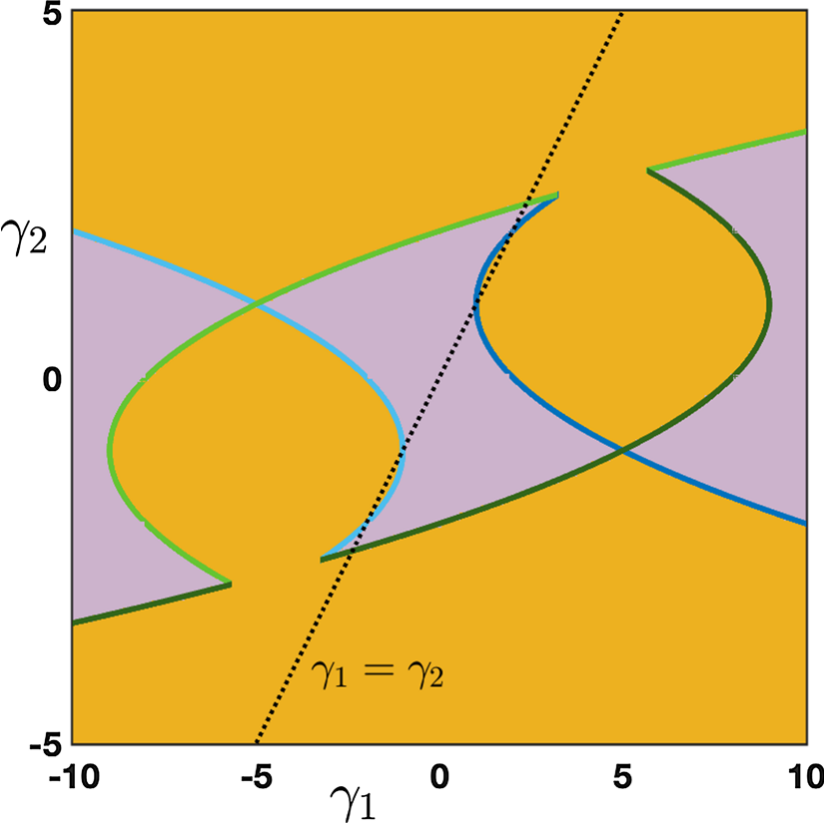
Stability/instability regions and their boundaries as a function of $(\gamma _1,\gamma _2)$ for (26) for fixed values of the hyperparameters $(\alpha ,\beta ,\lambda )$. The shaded orange region corresponds to an instability for (26) while the purple region corresponds to a stability for (26). The boundaries of the stability/instability regions are given by the intersections of the parametrized curves $\gamma _1=\chi (\gamma _2)$ (blue curve), $\gamma _1=-\chi (-\gamma _2)$ (light blue curve), $\gamma _1=\zeta (\gamma _2)$ (dark green curves) and $\gamma _1=-\zeta (-\gamma _2)$ (light green curves) where Eq. (26) is marginally stable. We represented the line $\gamma _1=\gamma _2$ (dotted black curve) which corresponds to the case studied in Fig. 11d with $(\beta ,\lambda )$ in Region $\mathbf {(III)}$ and $\alpha$ fixed in $(\Lambda ,\lambda +\beta )$ (Color figure online)

A complete and exhaustive characterization of all possible cases as a function of $\gamma _{1,2}$ and the hyper-parameters is beyond the scope of this paper. Nevertheless, we can make some few further remarks. The four above curves $\gamma _1=\chi (\gamma _2)$, $\gamma _1=-\chi (-\gamma _2)$, $\gamma _1=\zeta (\gamma _2)$ and $\gamma _1=-\zeta (-\gamma _2)$ form parabolas in the plane $(\gamma _1,\gamma _2)$ that can intersect and provide the boundaries of the stability regions. For example, we can notice that $\gamma _1=\zeta (\gamma _2)$ and $\gamma _1=-\zeta (-\gamma _2)$ intersect if and only if $\gamma _2=\pm \sqrt{\frac{2-\lambda -\beta }{\alpha }}$ whereas $\gamma _1=\chi (\gamma _2)$ and $\gamma _1=-\chi (-\gamma _2)$ can never intersect. We refer to Fig. 14 for an illustration of the stability regions and their boundaries in the case $(\alpha ,\beta ,\lambda )=(0.4,0.2,0.3)$, where the curves $\gamma _1=\chi (\gamma _2)$, $\gamma _1=-\chi (-\gamma _2)$, $\gamma _1=\zeta (\gamma _2)$ and $\gamma _1=-\zeta (-\gamma _2)$ are respectively represented by the blue, light blue, dark green and light green solid lines. Here, we see that stability can be obtained with large values of the feedforward connection strength $\gamma _1$, but this requires the feedback connections strength $\gamma _2$ to remain low. Of course, different qualitative behaviors and stability regions may be obtained for different choices of the hyperparameters $(\alpha ,\beta ,\lambda )$; while it is beyond the scope of the present study to characterize them all, it is important to point out that such a characterization is feasible using the present method, for any choice of the hyperparameters.

More interestingly, we can investigate the dependence of the wave speed as a function of the parameters $\gamma _1$ and $\gamma _2$. For example, when $\gamma _1=\chi (\gamma _2)$, we have that

$$\begin{aligned} \rho _{\chi (\gamma _2),\gamma _2}(\theta )=\exp \left( -{\textbf{i}}\frac{(\alpha -\lambda )\gamma _2+\beta \chi (\gamma _2)}{1-\beta \chi (\gamma _2)}\theta +{\mathcal {O}}(|\theta |^2)\right) , \text { as } \theta \rightarrow 0, \end{aligned}$$

such that the associated wave speed is given by

$$\begin{aligned} c_0^\chi =\frac{(\alpha -\lambda )\gamma _2+\beta \chi (\gamma _2)}{1-\beta \chi (\gamma _2)}, \end{aligned}$$

whose sign may vary as $\gamma _2$ is varied. We refer to the forthcoming Sect. 4.2.4 below for a practical example (see Fig. 18).

#### Continuous in Time Interpretation

As done in previous sections, we now perform a continuous in time limit of the model (26). With the same scaling on the hyperparameters

$$\begin{aligned} {\widetilde{\beta }}:=\frac{\beta }{\Delta t}, \quad {\widetilde{\lambda }}:=\frac{\lambda }{\Delta t}, \text { and } {\widetilde{\alpha }}:=\frac{\alpha }{\Delta t},\nonumber \\ \end{aligned}$$

we get that, at the limit $\Delta t\rightarrow 0$, the Eq. (26) becomes the following lattice ordinary differential equation

29 $$\begin{aligned} \frac{\textrm{d}}{\textrm{d}t}\textbf{u}_j(t)=({\widetilde{\beta }}\gamma _1+{\widetilde{\alpha }}\gamma _2)\textbf{u}_{j-1}(t)-({\widetilde{\beta }}+{\widetilde{\lambda }}+{\widetilde{\alpha }}\gamma _2^2)\textbf{u}_j(t)+{\widetilde{\lambda }}\gamma _2\textbf{u}_{j+1}(t), \quad t>0.\nonumber \\ \end{aligned}$$

The amplification factor function in this case is given by

$$\begin{aligned} \nu _{\gamma _1,\gamma _2}(\theta )=({\widetilde{\beta }}\gamma _1+{\widetilde{\alpha }}\gamma _2) e^{-{\textbf{i}}\theta }-({\widetilde{\beta }}+{\widetilde{\lambda }}+{\widetilde{\alpha }}\gamma _2^2)+{\widetilde{\lambda }}\gamma _2 e^{{\textbf{i}}\theta }, \quad \theta \in [-\pi ,\pi ], \end{aligned}$$

whose real part is given by

$$\begin{aligned} \textrm{Re}(\nu _{\gamma _1,\gamma _2}(\theta ))=({\widetilde{\beta }}\gamma _1+({\widetilde{\alpha }}+{\widetilde{\lambda }})\gamma _2)\cos (\theta )-({\widetilde{\beta }}+{\widetilde{\lambda }}+{\widetilde{\alpha }}\gamma _2^2), \quad \theta \in [-\pi ,\pi ]. \end{aligned}$$

When ${\widetilde{\beta }}\gamma _1+({\widetilde{\alpha }}+{\widetilde{\lambda }})\gamma _2>0$, we observe that

$$\begin{aligned} \underset{\theta \in [-\pi ,\pi ]}{\max }\textrm{Re}(\nu _{\gamma _1,\gamma _2}(\theta ))=\textrm{Re}(\nu _{\gamma _1,\gamma _2}(0))={\widetilde{\beta }}\gamma _1+({\widetilde{\alpha }}+{\widetilde{\lambda }})\gamma _2-({\widetilde{\beta }}+{\widetilde{\lambda }}+{\widetilde{\alpha }}\gamma _2^2), \end{aligned}$$

such that

$$\begin{aligned} \underset{\theta \in [-\pi ,\pi ]}{\max }\textrm{Re}(\nu _{\gamma _1,\gamma _2}(\theta ))=0 \Longleftrightarrow \gamma _1 = \frac{{\widetilde{\alpha }}\gamma _2^2-({\widetilde{\alpha }}+{\widetilde{\lambda }})\gamma _2+{\widetilde{\beta }}+{\widetilde{\lambda }}}{{\widetilde{\beta }}}. \end{aligned}$$

Whereas, when ${\widetilde{\beta }}\gamma _1+({\widetilde{\alpha }}+{\widetilde{\lambda }})\gamma _2<0$, we observe that

$$\begin{aligned} \underset{\theta \in [-\pi ,\pi ]}{\max }\textrm{Re}(\nu _{\gamma _1,\gamma _2}(\theta ))=\textrm{Re}(\nu _{\gamma _1,\gamma _2}(\pm \pi ))=-{\widetilde{\beta }}\gamma _1-({\widetilde{\alpha }}+{\widetilde{\lambda }})\gamma _2-({\widetilde{\beta }}+{\widetilde{\lambda }}+{\widetilde{\alpha }}\gamma _2^2), \end{aligned}$$

such that

$$\begin{aligned} \underset{\theta \in [-\pi ,\pi ]}{\max }\textrm{Re}(\nu _{\gamma _1,\gamma _2}(\theta ))=0 \Longleftrightarrow \gamma _1 = \frac{-{\widetilde{\alpha }}\gamma _2^2-({\widetilde{\alpha }}+{\widetilde{\lambda }})\gamma _2-{\widetilde{\beta }}-{\widetilde{\lambda }}}{{\widetilde{\beta }}}. \end{aligned}$$

**Fig. 15:**
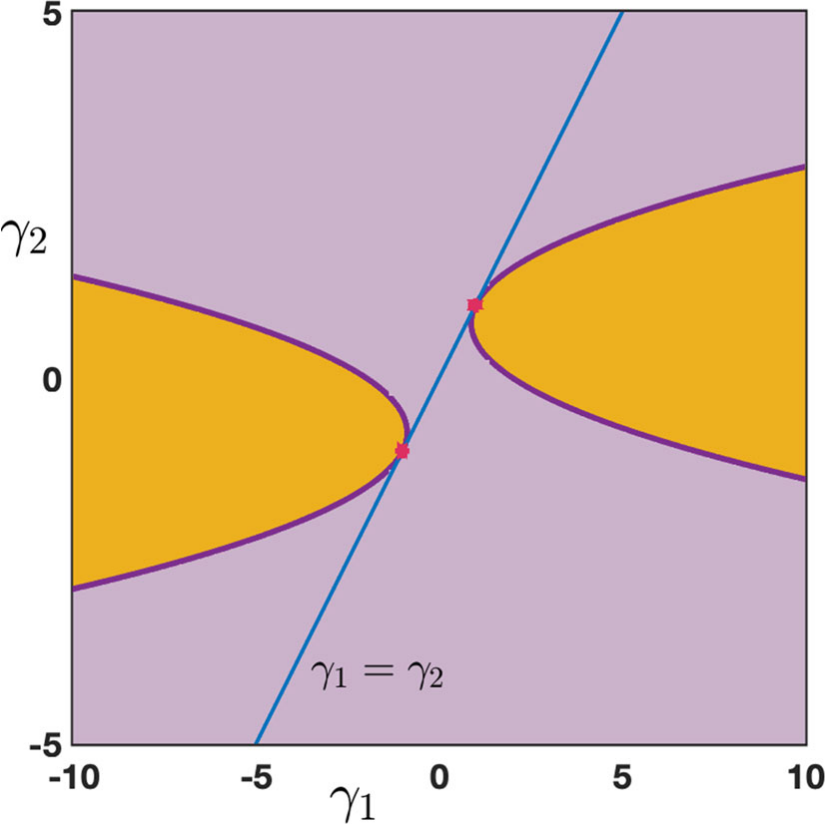
Stability/instability regions and their boundaries as a function of $(\gamma _1,\gamma _2)$ for (29) for any $({\widetilde{\alpha }},{\widetilde{\lambda }},{\widetilde{\beta }})$ fixed with ${\widetilde{\alpha }}={\widetilde{\lambda }}+{\widetilde{\beta }}$. The shaded orange region corresponds to an instability for (29) while the purple region corresponds to a stability for (29). The boundaries of the stability/instability regions are given by the intersections of the parabolas $\gamma _1= \frac{{\widetilde{\alpha }}\gamma _2^2-({\widetilde{\alpha }}+{\widetilde{\lambda }})\gamma _2+{\widetilde{\beta }}+{\widetilde{\lambda }}}{{\widetilde{\beta }}}$ and $\gamma _1= \frac{-{\widetilde{\alpha }}\gamma _2^2-({\widetilde{\alpha }}+{\widetilde{\lambda }})\gamma _2-{\widetilde{\beta }}-{\widetilde{\lambda }}}{{\widetilde{\beta }}}$ (magenta curves) where Eq. (29) is marginally stable. We represented the line $\gamma _1=\gamma _2$ (blue curve) which corresponds to the case studied in Fig. 13 (Color figure online)

As a consequence, the stability regions are determined by the locations of the parabolas $\gamma _2\mapsto \frac{{\widetilde{\alpha }}\gamma _2^2-({\widetilde{\alpha }}+{\widetilde{\lambda }})\gamma _2+{\widetilde{\beta }}+{\widetilde{\lambda }}}{{\widetilde{\beta }}}$ and $\gamma _2\mapsto \frac{-{\widetilde{\alpha }}\gamma _2^2-({\widetilde{\alpha }}+{\widetilde{\lambda }})\gamma _2-{\widetilde{\beta }}-{\widetilde{\lambda }}}{{\widetilde{\beta }}}$ in the plane $(\gamma _1,\gamma _2)$. We observe that they never intersect and are oriented in the opposite directions and refer to Fig. 15 for a typical configuration. Here, we see that the system is stable for a very large range of values of both $\gamma _1$ and $\gamma _2$. In particular, for large enough values of the feedback connection weight (e.g., $|\gamma _2|>3$), stability is guaranteed regardless of the value of the feedforward connection weight $\gamma _1$ (within a reasonable range, e.g., $\gamma _1\in (-10,10)$). This is the opposite behavior as that obtained for the discrete system in Fig. 14, where stability was impossible under the same conditions for $\gamma _{1,2}$. This highlights again the errors of interpretation that can potentially be caused by discrete approximation of a continuous system.

#### Fully Continuous Interpretation When ${\mathcal {W}}^f={\textbf{I}}_d+\xi _f {\textbf{A}}$ and ${\mathcal {W}}^b={\textbf{I}}_d+\xi _b {\textbf{A}}$

When ${\mathcal {W}}^f={\textbf{I}}_d+\xi _f {\textbf{A}}$ and ${\mathcal {W}}^b={\textbf{I}}_d+\xi _b {\textbf{A}}$, one can once again identify ${\mathcal {E}}_{j,k}^t$ as the approximation of some smooth function ${\mathcal {E}}(t,x,y)$ at $t_n=n\Delta t$, $x_j=j\Delta x$ and $y_k = k \Delta y$, along the three dimensions of time, network depth and neuronal layer width. We may rewrite (1) in this case as

$$\begin{aligned} (1-\beta )\frac{{\mathcal {E}}_j^{n+1}-{\mathcal {E}}_j^n}{\Delta t}&=\beta \nu \frac{{\mathcal {E}}_{j-1}^{n+1} -{\mathcal {E}}_j^{n+1}}{\Delta x}+ \lambda \nu \frac{ {\mathcal {E}}_{j+1}^{n}-{\mathcal {E}}_j^{n}}{\Delta x}-\alpha \nu \frac{ {\mathcal {E}}_{j}^{n}-{\mathcal {E}}_{j-1}^{n}}{\Delta x}\\&~~~+\beta \xi _f \kappa \frac{{\textbf{A}}}{\Delta y^2}{\mathcal {E}}_{j-1}^{n+1}+\alpha \xi _b \kappa \frac{{\textbf{A}}}{\Delta y^2}{\mathcal {E}}_{j-1}^{n}-2\alpha \xi _b \kappa \frac{{\textbf{A}}}{\Delta y^2}{\mathcal {E}}_{j}^{n}\\&\quad -\alpha \xi _b^2 \kappa \Delta y^2 \frac{{\textbf{A}}}{\Delta y^2} \frac{{\textbf{A}}}{\Delta y^2}{\mathcal {E}}_{j}^{n}+\lambda \xi _b \kappa \frac{{\textbf{A}}}{\Delta y^2}{\mathcal {E}}_{j+1}^{n}, \end{aligned}$$

such that in the limit $\Delta t\rightarrow 0$, $\Delta x\rightarrow 0$ and $\Delta y\rightarrow 0$ with $\nu$ and $\kappa$ fixed, we obtain the following partial differential equation

$$\begin{aligned} \partial _t {\mathcal {E}}(t,x,y) + \frac{\nu (\beta +\alpha -\lambda )}{1-\beta } \partial _x {\mathcal {E}}(t,x,y) =\kappa \frac{\beta \xi _f+(\lambda -\alpha )\xi _b}{1-\beta }\partial _y^2{\mathcal {E}}(t,x,y). \end{aligned}$$

As before, this is a diffusion equation along the *y* dimension, whose stability depends on the positivity of the diffusion coefficient, i.e., $\beta \xi _f+(\lambda -\alpha )\xi _b \ge 0$.

#### Application to a Ring Model of Orientations

Going back to our discrete system, in this section we consider the case where neurons within each layer encode for a given orientation in $[0,\pi ]$. Here, we have in mind visual stimuli which are made of a fixed elongated black bar on a white background with a prescribed orientation. We introduce the following matrix ${\textbf{A}}_{\textrm{per}}\in {\mathscr {M}}_d({\mathbb {R}})$ given by

$$\begin{aligned} {\textbf{A}}_{\textrm{per}}=\left( \begin{matrix} -2 &{} 1 &{} 0 &{} \cdots &{} 0 &{} 1 \\ 1 &{} -2 &{} 1 &{} \ddots &{} \ddots &{} 0 \\ 0 &{} \ddots &{} \ddots &{} \ddots &{} \ddots &{} \vdots \\ \vdots &{} \ddots &{} \ddots &{} \ddots &{} \ddots &{} \vdots \\ 0 &{} \ddots &{} \ddots &{} 1 &{} -2 &{} 1 \\ 1 &{} 0 &{} \cdots &{} 0 &{} 1 &{} -2 \end{matrix} \right) , \end{aligned}$$

which is nothing but the discretizing of the Laplacian with periodic boundary conditions. Indeed, for each ${\mathcal {E}}_j^n\in {\mathbb {R}}^d$, we assume that neuron ${\mathcal {E}}_{j,k}^n$ encodes for orientation $\frac{k}{d}\pi$ for $k=1,\ldots ,d$. We readily remark that $0\in \textrm{Spec}({\textbf{A}}_{\textrm{per}})$ with corresponding eigenvector $U_1=(1,\ldots ,1)^\textbf{t}\in {\mathbb {R}}^d$. Furthermore, we have:

- if $d=2m+1$ is odd, then $\lambda _p=-4\sin \left( \frac{p\pi }{d}\right) ^2$ with $p=1,\ldots , m$ is an eigenvalue of ${\textbf{A}}_{\textrm{per}}$ of multiplicity 2 with associated eigenvectors
  $$\begin{aligned} U_{2p}&=\left( \cos \left( \frac{2p\pi }{d}\right) ,\ldots ,\cos \left( \frac{2kp\pi }{d}\right) ,\ldots ,1\right) ^\textbf{t}\in {\mathbb {R}}^d,\\ U_{2p+1}&= \left( \sin \left( \frac{2p\pi }{d}\right) ,\ldots ,\sin \left( \frac{2kp\pi }{d}\right) ,\ldots ,0\right) ^\textbf{t}\in {\mathbb {R}}^d; \end{aligned}$$
- if $d=2m$ is even, then $\lambda _p=-4\sin \left( \frac{p\pi }{d}\right) ^2$ with $p=1,\ldots , m-1$ is an eigenvalue of ${\textbf{A}}_{\textrm{per}}$ of multiplicity 2 with associated eigenvectors $U_{2p}$ and $U_{2p+1}$ as above. And $\lambda =-4$ is a simple eigenvalue of ${\textbf{A}}_{\textrm{per}}$ with associated eigenvector $U_d=(-1,1,-1,1,\ldots ,-1,1)\in {\mathbb {R}}^d$.

**Fig. 16:**
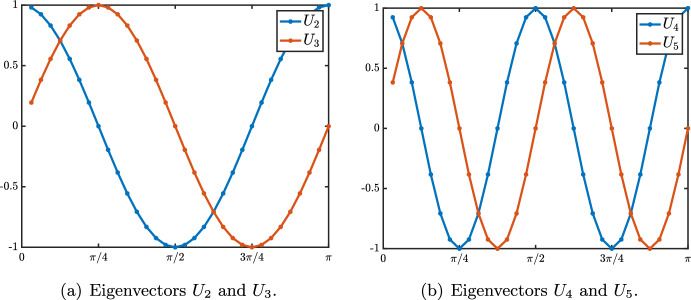
We plot the eigenvectors $U_{2p}$ and $U_{2p+1}$ for $p=1$ and $p=2$ as a function of $\frac{k}{d}\pi$ for $k=1,\ldots ,d$. We note that $U_{2p}$ and $U_{2p+1}$ encode the first Fourier modes. Here we have set $d=2^5$ (Color figure online)

It may be interesting to note that any linear combinations of $U_{2p}$ and $U_{2p+1}$ can always be written in the form

$$\begin{aligned} a U_{2p}+bU_{2p+1} = A \left( \cos \left( \frac{2p\pi }{d}+\varphi \right) ,\ldots ,\cos \left( \frac{2kp\pi }{d}+\varphi \right) ,\ldots ,\cos (\varphi )\right) ^\textbf{t}\in {\mathbb {R}}^d, \end{aligned}$$

where $A=\sqrt{a^2+b^2}>0$ and $\varphi =-\textrm{arctan}\left( \frac{b}{a}\right) \in (-\pi /2,\pi /2)$ whenever $a\ne 0$ and $b\ne 0$. This means that $U_{2p}$ and $U_{2p+1}$ span all possible translations modulo $[0,\pi ]$ of a fixed profile. We refer to Fig. 16 for a visualization of the first eigenvectors. In short, these eigenvectors $U_i$ implement a Fourier transform of the matrix ${\textbf{A}}_{\textrm{per}}$.

We now set ${\mathcal {W}}^b$ to be

$$\begin{aligned} {\mathcal {W}}^b=\frac{1}{2}{\textbf{I}}_d-\frac{1}{4}{\textbf{A}}_{\textrm{per}}=\left( \begin{matrix} 1 &{} -\frac{1}{4} &{} 0 &{} \cdots &{} 0 &{} -\frac{1}{4} \\ -\frac{1}{4} &{} 1 &{} -\frac{1}{4} &{} \ddots &{} \ddots &{} 0 \\ 0 &{} \ddots &{} \ddots &{} \ddots &{} \ddots &{} \vdots \\ \vdots &{} \ddots &{} \ddots &{} \ddots &{} \ddots &{} \vdots \\ 0 &{} \ddots &{} \ddots &{} -\frac{1}{4} &{} 1 &{} -\frac{1}{4} \\ -\frac{1}{4} &{} 0 &{} \cdots &{} 0 &{} -\frac{1}{4} &{} 1 \end{matrix} \right) , \end{aligned}$$

which means that ${\mathcal {W}}^b$ acts as a convolution with local excitation and lateral inhibition. From now on, to fix ideas, we will assume that $d=2m$ is even. We define the following matrix

$$\begin{aligned} P=\left( U_1,U_2,\ldots ,U_d\right) \in {\mathscr {M}}_d({\mathbb {R}}). \end{aligned}$$

As a consequence, we have the decomposition

$$\begin{aligned} P^{\textbf{t}}{\mathcal {W}}^bP= {\mathcal {D}}^b, \end{aligned}$$

with ${\mathcal {D}}^b=\textrm{diag}\left( \frac{1}{2},\frac{1}{2}-\frac{1}{4}\lambda _1,\frac{1}{2}-\frac{1}{4}\lambda _1,\ldots ,\frac{1}{2}-\frac{1}{4}\lambda _{m-1},\frac{1}{2}-\frac{1}{4}\lambda _{m-1},\frac{3}{2}\right) \in {\mathscr {M}}_d({\mathbb {R}})$. Now, for given values of the hyper-parameters $(\alpha ,\beta ,\lambda )$ with $\beta >0$, we set ${\mathcal {D}}^f:=\chi ({\mathcal {D}}^b)$ where the map $\chi$, defined in (27), is applied to the diagonal elements of ${\mathcal {D}}^b$, that is

$$\begin{aligned} {\mathcal {D}}^f=\textrm{diag}\left( \chi \left( \frac{1}{2}\right) ,\chi \left( \frac{1}{2}-\frac{1}{4}\lambda _1\right) ,\ldots ,\chi \left( \frac{1}{2}-\frac{1}{4}\lambda _{m-1}\right) ,\chi \left( \frac{3}{2}\right) \right) \in {\mathscr {M}}_d({\mathbb {R}}). \end{aligned}$$

**Fig. 17:**
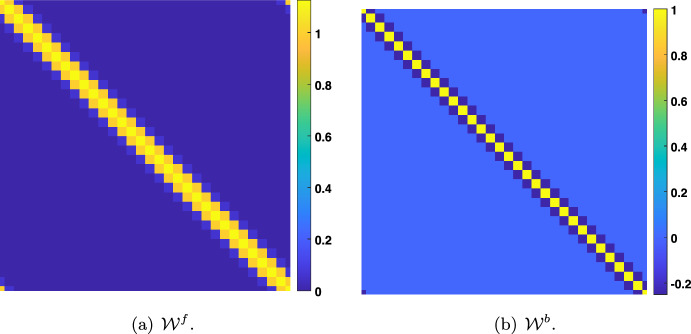
Illustration of the matrices ${\mathcal {W}}^f$ and ${\mathcal {W}}^b$ for $d=2^5$ neurons and values of the hyper-parameters fixed to $(\alpha ,\beta ,\lambda )=(0.1,0.1,0.5)$. Note the band structure of ${\mathcal {W}}^f$ with local excitation (Color figure online)

And then we set ${\mathcal {W}}^f:=P{\mathcal {D}}^fP^\textbf{t}$. We refer to Fig. 17 for an illustration of the structures of matrices ${\mathcal {W}}^f$ and ${\mathcal {W}}^b$. For large set of values of the hyper-parameters, ${\mathcal {W}}^f$ still present a band structure with positive elements on the diagonals indicating that ${\mathcal {W}}^f$ can also be interpreted as a convolution with local excitation. For the values of the hyper-parameters fixed in Fig. 17, the feedforward matrix ${\mathcal {W}}^f$ is purely excitatory.

Reproducing the analysis developed in the previous Sect. 4.2, we perform a change of orthonormal basis to express neural activities in terms of the relevant *assemblies* ${\mathcal {U}}_j^n:=P^t{\mathcal {E}}_j^n$. With $P{\mathcal {U}}_j^n:={\mathcal {E}}_j^n$, the recurrence equation becomes

$$\begin{aligned} {\mathcal {U}}_j^{n+1}-\beta {\mathcal {D}}^f {\mathcal {U}}_{j-1}^{n+1} =\alpha {\mathcal {D}}^b {\mathcal {U}}_{j-1}^n+\left[ (1-\beta -\lambda ){\textbf{I}}_d-\alpha {\mathcal {D}}^b {\mathcal {D}}^b\right] {\mathcal {U}}_j^{n} + \lambda {\mathcal {D}}^b {\mathcal {U}}_{j+1}^{n}. \end{aligned}$$

Then, if we denote by $\gamma _p$ the *p*th diagonal element of ${\mathcal {D}}^b$, then for each $p=1,\ldots , d$ the above recurrence writes

$$\begin{aligned} u_{j,p}^{n+1}-\beta \chi (\gamma _p) u_{j-1,p}^{n+1} =\alpha \gamma _p u_{j-1,p}^n +(1-\beta -\lambda -\alpha \gamma _p^2) u_{j,p}^{n} +\lambda \gamma _p u_{j+1,p}^{n}, \end{aligned}$$

where $u_{j,p}^n$ is the *p*th component (or neural assembly) of ${\mathcal {U}}_j^n$. For each $p=1,\ldots , d$, the associated amplification factor function reads

$$\begin{aligned} \rho _p(\theta ) = \frac{\alpha \gamma _p \left( e^{-{\textbf{i}}\theta }-\gamma _p\right) +1-\beta +\lambda \left( \gamma _p e^{{\textbf{i}}\theta }-1\right) }{1-\beta \chi (\gamma _p) e^{-{\textbf{i}}\theta }}, \quad \theta \in [-\pi ,\pi ], \end{aligned}$$

and with our specific choice of function $\chi$, we have that $\rho _p(0)=1$ with

$$\begin{aligned} \rho _p(\theta )=\exp \left( -{\textbf{i}}\frac{(\alpha -\lambda )\gamma _p+\beta \chi (\gamma _p)}{1-\beta \chi (\gamma _p)}\theta -\sigma _0^p\theta ^2+{\mathcal {O}}(|\theta |^3)\right) , \text { as } \theta \rightarrow 0, \end{aligned}$$

such that the associated wave speed is given by

$$\begin{aligned} c_0^p=\frac{(\alpha -\lambda )\gamma _p+\beta \chi (\gamma _p)}{1-\beta \chi (\gamma _p)}, \end{aligned}$$

and where we have set

$$\begin{aligned} \sigma _0^p=\frac{\alpha (1+4\lambda )\gamma _p^2+\beta +\lambda - (\alpha + \lambda )\gamma _p(\alpha \gamma _p^2 + \beta + \lambda )}{2(1-\beta \chi (\gamma _p))^2}. \end{aligned}$$

**Fig. 18:**
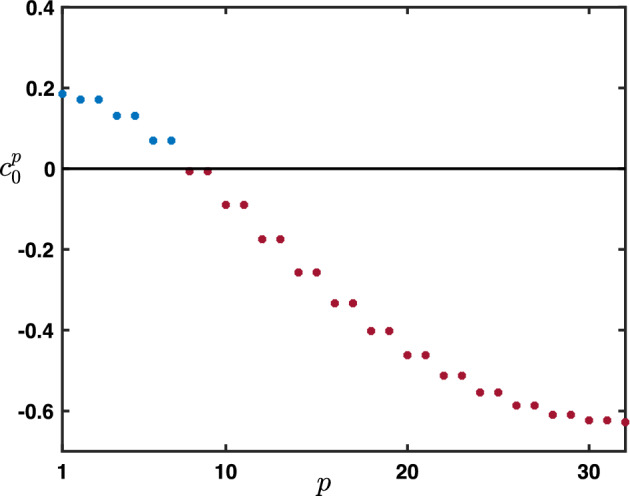
Plot of the wave speed $c_0^p$ for $p=1,\ldots ,d$ (colored dots). The color code (blue/red) refers to the sign of $c_0^p$: blue when positive and dark red when negative. Note that only the elements associated to the eigenvalues $\frac{1}{2}$, $\frac{1}{2}-\frac{1}{4}\lambda _1$, $\frac{1}{2}-\frac{1}{4}\lambda _2$ and $\frac{1}{2}-\frac{1}{4}\lambda _3$ are positive. We also remark that $c_0^p$ is a monotone decreasing function. Here $d=2^5$ and values of the hyper-parameters are fixed to $(\alpha ,\beta ,\lambda )=(0.1,0.1,0.5)$ (Color figure online)

From now on, we assume that we have tuned the hyper-parameters such that $|\rho _p(\theta )|<1$ for all $\theta \in [-\pi ,\pi ]\backslash \left\{ 0\right\}$ and each $p=1,\ldots ,d$. This can in fact be systematically checked numerically for a given set of hyper-parameters. We report in Fig. 18 the shape of $p\mapsto c_0^p$ for the same values of the hyper-parameters as the ones in Fig. 17 and $d=2^5$. We first remark that $p\mapsto c_0^p$ is a monotone decreasing map, and in our specific case we have

$$\begin{aligned} c^d_0<c^{d-1}_0=c^{d-2}_0<\cdots<c^9_0=c^8_0<0<c^7_0=c^6_0<c^5_0=c^4_0<c^3_0=c^2_0<c^1_0. \end{aligned}$$

The fact that wave speeds come in pair for $p=2,\ldots d-1$ is reminiscent of the spectral properties of ${\textbf{A}}_{\textrm{per}}$ which has $m-1$ eigenvalues with multiplicity 2 when $d=2m$ is even.

Given a fixed input entry ${\mathcal {E}}_0\in {\mathbb {R}}^d$ presented at $j=0$ to the network continually at each time step, we can deduce which components of ${\mathcal {E}}_0\in {\mathbb {R}}^d$ will be able to propagate forward through the network. More precisely, we can decompose ${\mathcal {E}}_0$ along the basis $\left( U_1,\ldots U_d\right)$ of eigenvectors, that is

$$\begin{aligned} {\mathcal {E}}_0= \sum _{p=1}^{d}a_p U_{p}, \end{aligned}$$

for some real coefficients $a_p$ for $p=1,\ldots ,d$. Assuming that the network was at rest initially, we get that the dynamics along each eigenvector (or neural assembly) is given by

30 $$\begin{aligned} \left\{ \begin{aligned} u_{j,p}^{n+1}-\beta \chi (\gamma _p) u_{j-1,p}^{n+1}&=\alpha \gamma _p u_{j-1,p}^n +(1-\beta -\lambda -\alpha \gamma _p^2) u_{j,p}^{n} +\lambda \gamma _p u_{j+1,p}^{n}, \quad j\ge 1, \quad n\ge 0,\\ u_{0,p}^n&=a_p, \quad n\ge 0,\\ u_{j,p}^0&=0, \quad j\ge 1. \end{aligned} \right. \nonumber \\ \end{aligned}$$

Thus, we readily obtain that

$$\begin{aligned} {\mathcal {E}}_j^n= \sum _{p=1}^{d}u_{j,p}^{n} U_{p}, \quad j\ge 1, \quad n\ge 1, \end{aligned}$$

where $u_{j,p}^{n}$ is a solution to (30).

**Fig. 19:**
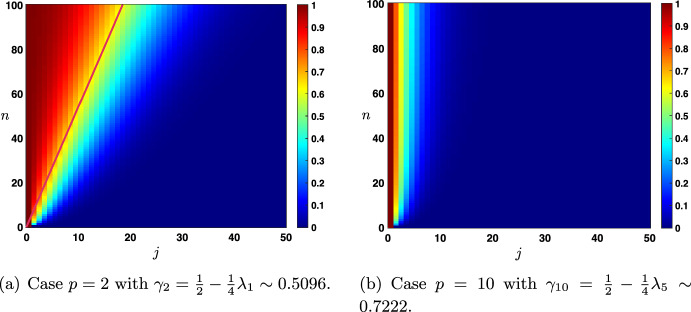
Space-time plot of the solution of the recurrence equation for (30) for $p=2$ and $p=10$ associated to respectively positive wave speed $c_0^2>0$ and negative wave speed $c_0^{10}<0$. The neural assembly associated with the 2nd eigenvector of the connectivity matrix propagates its input signal into the network at constant speed; but the neural assembly associated with the 10th eigenvector does not propagate the signals it receives on the input layer (Color figure online)

As a consequence, the monotonicity property of the map $p\mapsto c_0^p$ indicates that the homogeneous constant mode $U_1$ is the fastest to propagate forward into the network with associated spreading speed $c^1_0$, it is then followed by the modes $(U_{2},U_{3})$ propagating at speed $c^2_0=c^3_0$. In our numerics, we have set the parameters such that $c^1_0 \approx c^2_0=c^3_0$ with a significant gap with the other wave speeds. Lets us remark, that all modes $U_p$ with $p\ge 8$ are not able to propagate into the network (see Fig. 19). Thus our architecture acts as a mode filter.

Even more precisely, let us remark that the sequence $\left( a_p\left( \frac{\alpha \gamma _p+\beta \chi (\gamma _p)}{\lambda \gamma _p}\right) ^j\right) _{j\ge 0}$ is a stationary solution of (30) which remains bounded whenever *p* is such that the associated wave speed is negative, that is $c_0^p<0$, since in that case, one has $\alpha \gamma _p+\beta \chi (\gamma _p)<\lambda \gamma _p$. The solution ${\mathcal {E}}_j^n$ can then be approximated as

$$\begin{aligned} {\mathcal {E}}_j^n{} & {} \simeq \sum _{p~:~ c_0^p>0} \frac{a_p}{2}\left( 1-\textrm{erf}\left( \frac{j-c_0^pn}{\sqrt{4\sigma _0^pn}}\right) \right) U_{p}\\{} & {} \quad +\sum _{p~:~ c_0^p<0} a_p\left( \frac{\alpha \gamma _p+\beta \chi (\gamma _p)}{\lambda \gamma _p}\right) ^jU_{p}, \quad j\ge 1, \quad n\ge 1, \end{aligned}$$

**Fig. 20:**
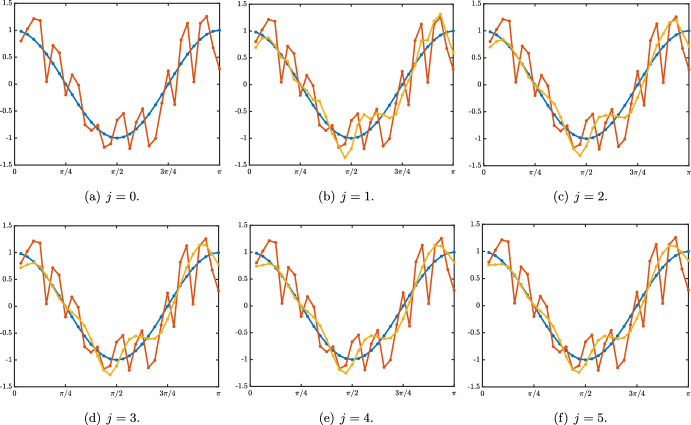
A fixed input ${\mathcal {E}}_0$ (red) which is the superposition of a tuned curve at $\vartheta =0$ (blue) with some fixed random noise is presented at layer $j=0$. Profile (yellow) of ${\mathcal {E}}_j^n$ at time iteration $n=200$ along the first layers of the network $j\in \left\{ 1,2,3,4,5\right\}$. All profiles are plotted as a function of $\frac{k}{d}\pi$ for $k=1,\ldots ,d$ (Color figure online)

**Fig. 21:**
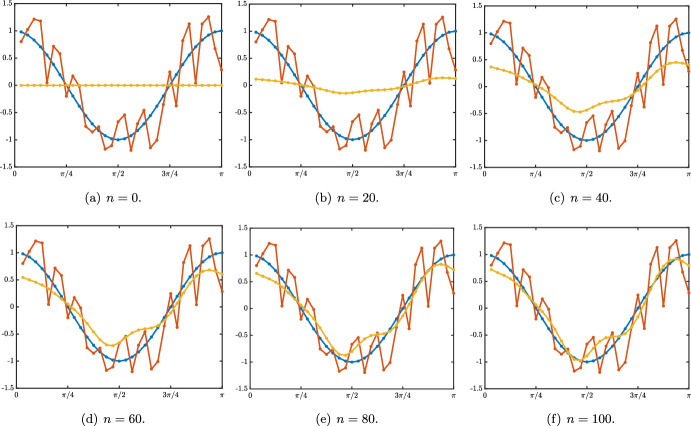
Time evolution of ${\mathcal {E}}_j^n$ at layer $j=10$ (yellow) for $n\in \left\{ 0,20,40,60,80,100\right\}$ with fixed input ${\mathcal {E}}_0$ at layer $j=0$ (red). The input ${\mathcal {E}}_0$ is the superposition of a tuned curve at $\vartheta =0$ (blue) with some fixed random noise. All profiles are plotted as a function of $\frac{k}{d}\pi$ for $k=1,\ldots ,d$ (Color figure online)

This is illustrated by a first example simulation in Figs. 20 and 21. We present at $j=0$ a fixed input ${\mathcal {E}}_0$ which is generated as the superposition of a tuned curve at $\vartheta =0$ (blue) with some fixed random noise: namely we select $a_1=0$, $a_2=1$, $a_3=0$ and all other coefficients $a_p$ for $p=4,\ldots ,d$ are drawn from a normal law with an amplitude pre-factor of magnitude $\varepsilon$ set to $\varepsilon =0.1$. The shape of the input ${\mathcal {E}}_0$ is shown in Fig. 20a. The profile of ${\mathcal {E}}_j^n$ at time iteration $n=200$ along the first layers of the network $j\in \left\{ 1,2,3,4,5\right\}$ is given in Fig. 20b–f respectively. We first observe that the network indeed acts as a filter since across the layers of the network the solution profile ${\mathcal {E}}_j^n$ presents less noise and gets closer to the tuned curve at $\vartheta =0$. Let us also remark that the filtering is more efficient for layers away from the boundary and is less efficient for those layers near the boundary. This is rather natural since the impact of the input ${\mathcal {E}}_0$ is stronger on the first layers. We see that already at layer $j=5$, we have almost fully recovered the tuned curve at $\vartheta =0$ (see Fig. 20f). On the other hand, in Fig. 21, we show the time evolution of ${\mathcal {E}}_j^n$ at a fixed layer far away from the boundary, here $j=10$. Initially, at $n=0$, the layer is inactivated (see Fig. 21a), and we see that after several time iterations that the solution profile ${\mathcal {E}}_j^n$ start to be activated. It is first weakly tuned (see Fig. 21b–d) and then it becomes progressively fully tuned and converges to the tuned curve at $\vartheta =0$ (see Fig. 21e, f).

**Fig. 22:**
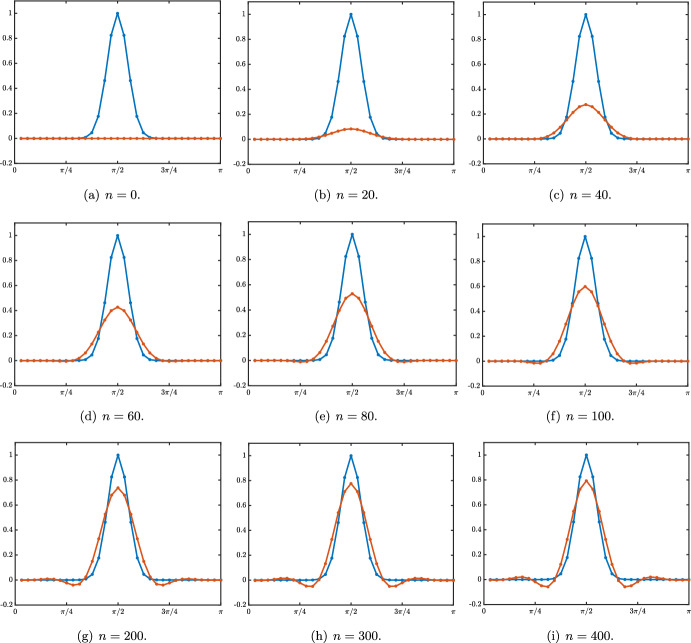
Time evolution of ${\mathcal {E}}_j^n$ at layer $j=10$ (orange) for $n\in \left\{ 0,20,40,60,80,100,200,300,400\right\}$ with fixed input ${\mathcal {E}}_0$ at layer $j=0$ (blue). The input ${\mathcal {E}}_0$ is a Gaussian centered at $\vartheta =\pi /2$. All profiles are plotted as a function of $\frac{k}{d}\pi$ for $k=1,\ldots ,d$ (Color figure online)

In a second example simulation (Fig. 22), we highlight the dynamics of the different modes in a situation where the input is a narrow Gaussian profile (close to a Dirac function), with a superposition of various Fourier modes. As expected from the different values of the propagation speed $c_0$ (Fig. 18), we see that the mode associated with the first Fourier component is the first to reach layer $j=10$, later followed by successive modes associated with later Fourier components. In other words, this hierarchically higher layer $j=10$ first receives information about the coarse spatial structure of the input signal, and then gradually about finer and finer spatial details.

### Summary

In this section, we saw that the results obtained initially (The Identity Case) with the amplification function can be extended to more realistic situations with forward and backward connection matrices, for instance implementing (residual) convolutions or orientation processing. When we consider neural *assemblies* capturing the principal components of the connection matrices, we see that each assembly can be treated independently in terms of stability and signal propagation speed and direction. The exact behavior of the system will depend on the actual connection matrices (and thus on the function that they implement in the neural network), but the important point is that our generic framework can always be applied in practice. In some example cases (ring model of orientations), we saw that only a few assemblies support signal propagation (implying that the system acts as a filter on its inputs), and these assemblies propagate information at different speeds (implementing a coarse-to-fine analysis). In other cases (e.g., Fig. 12), we have even seen that distinct assemblies can simultaneously propagate information in opposite directions, with one assembly supporting feedforward propagation while another entails feedback propagation.

We have extended our equations to the continuous limit in time, and found that the amplification factor function can give rise to qualitatively different stability regions compared to the discrete model. This served as a cautionary note for situations where the discrete implementation must be chosen; in that case, using smaller time steps will be preferable, because it makes such discrepancies less likely.

Finally, we also showed that it is possible to consider fully continuous versions of our dynamic system, where not only time but also network depth and neural layer width are treated as continuous variables. This gives rise to diffusion equations, whose stability can also be characterized as a function of hyperparameter values.

In the following, we address possible extensions of the model to more sophisticated and more biologically inspired neural architectures, taking into account the significant communication delays between layers.

## Extension of the Model: Taking into Account Transmission Delays

**Fig. 23:**
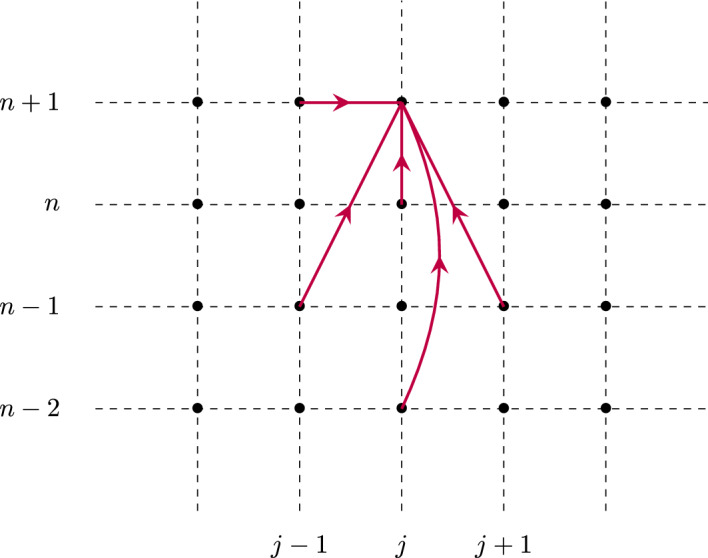
Illustration of the network structure of model (31) for $k=1$ where the red arrows indicate the contributions leading to the update of ${\mathcal {E}}_j^{n+1}$ (Color figure online)

Deep feedforward neural networks typically implement *instantaneous* updates, as we did in Eq. (1) with our feedforward term ${\mathcal {E}}_j^{n+1}=\beta {\mathcal {W}}^f {\mathcal {E}}_{j-1}^{n+1}+...$. Similarly, artificial recurrent neural networks sequentially update their activity from *one time step to the next*, as we did with the other terms in our Eq. (1) (memory term, feedforward and feedback prediction error correction terms): ${\mathcal {E}}_j^{n+1}=...+ \alpha ({\mathcal {W}}^b) ^{\textbf{t}}{\mathcal {E}}_{j-1}^{n}+(1-\beta -\lambda ){\mathcal {E}}_j^{n}-\alpha ({\mathcal {W}}^b )^{\textbf{t}} {\mathcal {W}}^b {\mathcal {E}}_j^{n} + \lambda {\mathcal {W}}^b {\mathcal {E}}_{j+1}^{n}$. However, in the brain there are significant transmission delays whenever neural signals travel from one area to another. These delays could modify the system’s dynamics and its stability properties. Therefore, in this section we modify model (1) by assuming that it takes *k* time steps to receive information from a neighboring site in the feedback/feedforward dynamics, namely we consider the following recurrence equation

31 $$\begin{aligned} {\mathcal {E}}_j^{n+1}-\beta {\mathcal {W}}^f {\mathcal {E}}_{j-1}^{n+1}{} & {} =\alpha ({\mathcal {W}}^b) ^{\textbf{t}}{\mathcal {E}}_{j-1}^{n-k}+(1-\beta -\lambda ){\mathcal {E}}_j^{n}-\alpha ({\mathcal {W}}^b )^{\textbf{t}} {\mathcal {W}}^b {\mathcal {E}}_j^{n-2k}\nonumber \\{} & {} \quad + \lambda {\mathcal {W}}^b {\mathcal {E}}_{j+1}^{n-k}, \end{aligned}$$

where $k\ge 1$ is some given fixed integer (see Fig. 23 for an illustration with $k=1$), and we refer to Pang (2022) for the justification of the derivation of the model. (Note in particular that we did not modify the *instantaneous* nature of our feedforward updating term ${\mathcal {E}}_j^{n+1}=\beta {\mathcal {W}}^f {\mathcal {E}}_{j-1}^{n+1}+\cdots$. This is because, as motivated in Choksi et al. (2021) and Pang (2022), we aim for the feedforward part of the system to be compatible with state-of-the-art deep convolutional neural networks, and merely wish to investigate how adding recurrent dynamics can modify its properties.) We may already notice that when $k=0$, we recover our initial model (1). In what follows, for the mathematical analysis, we restrict ourselves to the identity case ${\mathcal {W}}^f={\mathcal {W}}^n={\textbf{I}}_d$ and when the model is set on ${\mathbb {Z}}$. Indeed, our intention is to briefly explain what could be the main new propagation properties that would emerge by including transmission delays. Thus, we consider

32 $$\begin{aligned} e_j^{n+1}-\beta e_{j-1}^{n+1}=\alpha e_{j-1}^{n-k}+(1-\beta -\lambda )e_j^{n} -\alpha e_j^{n-2k} + \lambda e_{j+1}^{n-k}, \quad j\in {\mathbb {Z}}. \end{aligned}$$

Let us also note that the system (32) depends on a “history” of $2k+1$ time steps; thus one needs to impose $2k+1$ initial conditions:

$$\begin{aligned} e_j^m=h_j^m,\quad m=0,\ldots ,2k, \quad j\in {\mathbb {Z}}, \end{aligned}$$

for $2k+1$ given sequences $(h_j^m)_{j\in {\mathbb {Z}}}$ with $m=0,\ldots ,2k$.

To proceed in the analysis, we first introduce a new vector unknown capturing each layer’s recent history:

$$\begin{aligned} {\textbf{E}}_j^n:=\left( \begin{array}{c}e_j^{n-2k}\\ \vdots \\ e_{j}^{n-2} \\ e_{j}^{n-1} \\ e_{j}^{n}\end{array}\right) \in {\mathbb {R}}^{2k+1}, \quad n\ge 1, \quad j\in {\mathbb {Z}}, \end{aligned}$$

such that the above recurrence (32) can then be rewritten as

33 $$\begin{aligned} {\textbf{E}}_j^{n+1}-\beta Q_{-1}{\textbf{E}}_{j-1}^{n+1}=\alpha Q_1 {\textbf{E}}_{j-1}^{n}+Q_0{\textbf{E}}_j^{n}+\lambda Q_1 {\textbf{E}}_{j+1}^{n},\quad n\ge 1, \quad j\in {\mathbb {Z}}, \end{aligned}$$

where the matrices $Q_1,Q_0,Q_{-1}\in {\mathscr {M}}_{2k+1}({\mathbb {R}})$ are defined as follows

$$\begin{aligned} Q_{0}=\left( \begin{matrix} 0 &{} 1 &{} 0 &{} \cdots &{} \cdots &{} 0 \\ \vdots &{} \ddots &{} \ddots &{} \ddots &{} \ddots &{} \vdots \\ \vdots &{} \ddots &{} \ddots &{} \ddots &{} \ddots &{} \vdots \\ \vdots &{} \ddots &{} \ddots &{} \ddots &{} \ddots &{} 0 \\ 0 &{} \ddots &{} \ddots &{} \ddots &{} 0 &{} 1 \\ -\alpha &{} 0 &{} \cdots &{} \cdots &{} 0 &{} 1-\beta -\lambda \end{matrix} \right) , \end{aligned}$$

and $Q_{\pm 1}$ have a single nonzero element on their last row:

$$\begin{aligned} \left( Q_{-1}\right) _{2k+1,2k+1}=1, \quad \left( Q_{1}\right) _{2k+1,k+1}=1. \end{aligned}$$

### Mathematical Study of the Recurrence Eq. (33)

We now postulate an ansatz of the form $\rho ^ne^{{\textbf{i}}\theta j}{\textbf{E}}$ for some non zero vector ${\textbf{E}}\in {\mathbb {C}}^{2k+1}$, and obtain

$$\begin{aligned} \underbrace{\left( \rho \left[ {\textbf{I}}_{2k+1}-\beta e^{-{\textbf{i}}\theta } Q_{-1}\right] -Q_0 -(\alpha e^{-{\textbf{i}}\theta }+\lambda e^{{\textbf{i}}\theta })Q_1 \right) }_{:={\mathcal {A}}_k(\rho ,\theta )}{\textbf{E}}=\left( \begin{array}{c} 0\\ \vdots \\ 0\end{array}\right) \end{aligned}$$

which is equivalent to

$$\begin{aligned} \det \left( \rho \left[ {\textbf{I}}_{2k+1}-\beta e^{-{\textbf{i}}\theta } Q_{-1}\right] -Q_0 -(\alpha e^{-{\textbf{i}}\theta }+\lambda e^{{\textbf{i}}\theta })Q_1 \right) =0, \end{aligned}$$

that is

34 $$\begin{aligned} (1-\beta e^{-{\textbf{i}}\theta })\rho ^{2k+1}-\rho ^{2k}\left( 1-\beta -\lambda \right) -\rho ^k\left( \alpha e^{-{\textbf{i}}\theta }+\lambda e^{{\textbf{i}}\theta }\right) +\alpha =0. \end{aligned}$$

The above system has $2k+1$ roots in the complex plane that we denote $\rho _m(\theta )$ for $m=1,\ldots 2k+1$. We remark at $\theta =0$, $\rho =1$ is always a root of the equation since in this case (34) reduces to

35 $$\begin{aligned} (1-\beta )\rho ^{2k+1}-\rho ^{2k}\left( 1-\beta -\lambda \right) -\rho ^k\left( \alpha +\lambda \right) +\alpha =0. \end{aligned}$$

By convention, we assume that $\rho _1(0)=1$. We further note that ${\textbf{E}}_1=(1,\ldots ,1)^\textbf{t}$ is the associated eigenvector. As usual, we can perform a Taylor expansion of $\rho _1$ near $\theta =0$ and we obtain that

$$\begin{aligned} \rho _1(\theta )=\exp \left( -{\textbf{i}}\frac{\alpha +\beta -\lambda }{1-\beta +k(\lambda -\alpha )}\theta +{\mathcal {O}}(|\theta |^2)\right) , \text { as } \theta \rightarrow 0, \end{aligned}$$

so that the associated wave speed is this time given by

$$\begin{aligned} c_0^k=\frac{\alpha +\beta -\lambda }{1-\beta +k(\lambda -\alpha )}, \end{aligned}$$

and depends explicitly on the delay *k*. We readily conclude that:

- When $\alpha <\lambda$, then $c_0^k$ is well defined for all values of *k*. Furthermore, the amplitude of the wave speed $k\mapsto |c_0^k|$ decreases as *k* increases with $|c_0^k|\rightarrow 0$ as $k\rightarrow +\infty$. That is, the activity waves may go forward or backward (depending on the hyperparameter values), but the transmission delay always slows down their propagation.
- When $\alpha =\lambda$, then $c_0^k=\frac{\beta }{1-\beta }>0$ is independent of the delay *k*. This is compatible with our implementation choice, where the initial feedforward propagation term (controlled by $\beta$) is not affected by transmission delays.
- When $\lambda <\alpha$, then $c_0^k$ is well defined whenever $k\ne \frac{1-\beta }{\alpha -\lambda }>0$. Furthermore, the wave speed $c_0^k>0$ for $1\le k < \frac{1-\beta }{\alpha -\lambda }$ and increases with the delay *k* on that interval. That is, in this parameter range neural activity waves propagate forward and, perhaps counterintuively, accelerate when the transmission delay increases. On the other hand $c_0^k<0$ for $k>\frac{1-\beta }{\alpha -\lambda }$ and $k\mapsto |c_0^k|$ decreases as *k* increases on that domain with $|c_0^k|\rightarrow 0$ as $k\rightarrow +\infty$. In this parameter range, waves propagate backward, and decelerate when the transmission delay increases.

Coming back to (35), we can look for other potential roots lying on the unit disk, i.e., marginally stable solutions. That is we look for $\omega \in (0,2\pi )$ such that $\rho =e^{{\textbf{i}}\omega }$. We obtain a system of two equations

36 $$\begin{aligned} \left\{ \begin{aligned} (1-\beta )\cos ((2k+1)\omega )-(1-\beta -\lambda )\cos (2k\omega )-(\alpha +\lambda )\cos (k\omega )+\alpha&=0,\\ (1-\beta )\sin ((2k+1)\omega )-(1-\beta -\lambda )\sin (2k\omega )-(\alpha +\lambda )\sin (k\omega )&=0. \end{aligned} \right. \nonumber \\ \end{aligned}$$

**Fig. 24:**
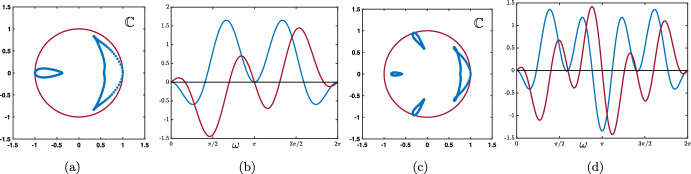
Spectral configurations in the case $k=1$ (**a**) and $k=2$ (**c**) with tangency points associated to $\theta =0$ in (35). In (**b**) and (**d**), we plot the left-hand side of the equations defining system (36) in the case $k=1$ and $k=2$ respectively where the first component is in blue and the second component in dark red. For $k=1$, we have a solution at $\omega =0$ and $\omega =\pi$ which can be seen in panel (**a**) with the tangency points at $\pm 1$. For $k=2$, we have three solutions $\omega =0$ and $\omega \sim 1.885$ and $\omega \sim 2\pi -1.885$ which can be seen in panel (**c**) with the tangency points at 1 and $e^{\pm {\textbf{i}}1.885}$. Parameters are set to $(\alpha ,\beta ,\lambda )=(0.4,0.3,0.3)$ in (**a**)–(**b**) and $(\alpha ,\beta ,\lambda )=(0.3,0.3292,0.3)$ (Color figure online)

**Case** $k=1$. When $k=1$, coming back to (35), we see that the two other roots are real and given by $-\frac{\lambda }{2(1-\beta )}\pm \frac{\sqrt{\lambda ^2+4\alpha (1-\beta )}}{2(1-\beta )}$, such that when $\alpha +\beta +\lambda =1$ the negative root is precisely $-1$ such that $\omega =\pi$ is a solution which we assume, without loss of generality, to be the second root, that is $\rho _2(0)=-1$ whenever $\alpha +\beta +\lambda =1$. In this specific case, the associated eigenvector is ${\textbf{E}}_{-1}=(1,-1,1)^\textbf{t}$. Recall that ${\textbf{E}}$ reflects the *history* of activity across the $2k+1=3$ preceding time steps. In this case, the eigenvector ${\textbf{E}}_{-1}$ is a rapid alternation of activity, i.e., an oscillation. We refer to Fig. 24a for an illustration of the spectral configuration in that case. We can perform a Taylor expansion of $\rho _2$ near $\theta =0$ and we obtain that

$$\begin{aligned} \rho _2(\theta )=-\exp \left( -{\textbf{i}}\frac{\alpha +\beta -\lambda }{5-5\beta -\alpha -3\lambda }\theta +{\mathcal {O}}(|\theta |^2)\right) , \text { as } \theta \rightarrow 0, \end{aligned}$$

which provides an associated wave speed ${\widetilde{c}}_0$ given by

$$\begin{aligned} {\widetilde{c}}_0=\frac{\alpha +\beta -\lambda }{5-5\beta -\alpha -3\lambda }. \end{aligned}$$

As a consequence of the above analysis, if ${\textbf{G}}_j^n$ denotes the fundamental solution of (33) starting from a Dirac delta mass centered at $j=0$ along the direction ${\textbf{E}}\in {\mathbb {R}}^3$, then we have the following representation for ${\textbf{G}}_j^n$:

- If $$\alpha +\beta +\lambda \ne 1$$, then
  $$\begin{aligned} {\textbf{G}}_j^n\approx \frac{1}{\sqrt{4\pi \sigma _0^k n}}\exp \left( -\frac{|j-c_0^kn|^2}{4\sigma _0^k n}\right) \left\langle (0,0,1)^\textbf{t}, \pi _1({\textbf{E}})\right\rangle _{{\mathbb {R}}^3}, \end{aligned}$$
  where $\pi _1$ is the spectral projection of ${\mathbb {R}}^3$ along the direction ${\textbf{E}}_1$ and $\left\langle \cdot , \cdot \right\rangle _{{\mathbb {R}}^3}$ is the usual scalar product. Here $\sigma _0^k$ is some positive constant that can be computed explicitly by getting the higher order expansion of $\rho _1(\theta )$.
- If $$\alpha +\beta +\lambda =1$$, then
  $$\begin{aligned} {\textbf{G}}_j^n&\approx \frac{1}{\sqrt{4\pi \sigma _0^k n}}\exp \left( -\frac{|j-c_0^kn|^2}{4\sigma _0^k n}\right) \left\langle (0,0,1)^\textbf{t}, \pi _1({\textbf{E}})\right\rangle _{{\mathbb {R}}^3}\\&\quad +\frac{(-1)^n}{\sqrt{4\pi \widetilde{\sigma _0} n}}\exp \left( -\frac{|j-{\widetilde{c}}_0n|^2}{4\widetilde{\sigma _0} n}\right) \left\langle (0,0,1)^\textbf{t}, \pi _{-1}({\textbf{E}})\right\rangle _{{\mathbb {R}}^3}, \end{aligned}$$
  where $\pi _{-1}$ is the spectral projection of ${\mathbb {R}}^3$ along the direction ${\textbf{E}}_{-1}$. Here ${\widetilde{\sigma }}_0$ is some positive constant that can be computed explicitly by getting the higher order expansion of $\rho _2(\theta )$.

**Fig. 25:**
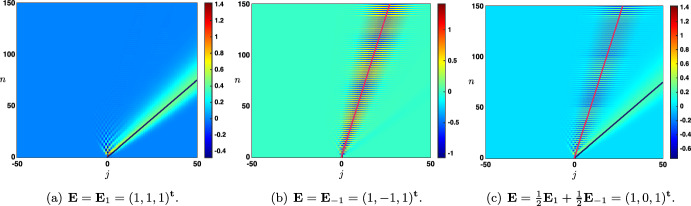
Space-time plots of the last component of the rescaled fundamental solution ${\textbf{E}}_j^n$ of (33) starting from a Dirac delta mass centered at $j=0$ along different directions ${\textbf{E}}$ when $k=1$ and $\alpha +\beta +\lambda =1$. **a** When ${\textbf{E}}={\textbf{E}}_1$ (constant history of activity) is the eigenvector associated to ${\mathcal {A}}_1(1,0)$ we observe propagation at wave speed $c_0^1$. **b** When ${\textbf{E}}={\textbf{E}}_{-1}$ (oscillating history of activity) is the eigenvector associated to ${\mathcal {A}}_1(-1,0)$ we observe propagation of an oscillatory wave at wave speed ${\widetilde{c}}_0$. **c** When ${\textbf{E}}$ is a linear combination of ${\textbf{E}}_{1}$ and ${\textbf{E}}_{-1}$, we observe two propagating waves (one of them oscillating) at wave speed $c_0^1$ and ${\widetilde{c}}_0$ respectively. Parameter values are set to $(\alpha ,\beta ,\lambda )=(0.4,0.3,0.3)$ (Color figure online)

In Fig. 25, we illustrate the previous results in the case where $\alpha +\beta +\lambda =1$. In panel (a), we have set ${\textbf{E}}={\textbf{E}}_1$ (a constant history of activity over the previous 3 time steps), such that $\pi _1({\textbf{E}}_1)={\textbf{E}}_1$ and $\pi _{-1}({\textbf{E}}_{1})=0_{{\mathbb {R}}^3}$ so that we only observe a Gaussian profile propagating at speed $c_0^k$. On the other hand in panel (b), we have set ${\textbf{E}}={\textbf{E}}_{-1}$ (an oscillating history of activity over the previous 3 time steps), such that $\pi _1({\textbf{E}}_{-1})=0_{{\mathbb {R}}^3}$ and $\pi _{-1}({\textbf{E}}_{-1})={\textbf{E}}_{-1}$ so that we only observe an oscillating (in time) Gaussian wave profile propagating at speed ${\widetilde{c}}_0$. Note that in this case, the period of the oscillation is necessarily equal to 2*k*, i.e., twice the transmission delay between layers. Finally in panel (c), we observe a super-position of the two Gaussian profiles propagating at speed $c_0^1$ and ${\widetilde{c}}_0$.

**Fig. 26:**
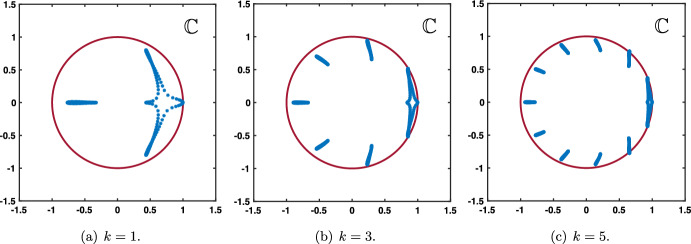
Destabilization of the spectrum by increasing the delay *k* while keeping fixed the hyper-parameters to $(\alpha ,\beta ,\lambda )=(0.3,0.1,0.3)$ (Color figure online)

**Case** $k\ge 2$. Studying the above system (36) in full generality is a very difficult task. We refer to Fig. 24c and d for an illustration in the case $k=2$ with three tangency points associated to $\theta =0$ lying on the unit circle. Increasing the delay *k* while keeping fixed the other hyper-parameters $(\alpha ,\beta ,\lambda )$ will generically tend to destabilize the spectrum (as shown in Fig. 26).

### Continuous in Time Interpretation

As done before, we now re-examine our model (with transmission delays) in the time-continuous limit. First, we recall our notations for the scaled parameters

$$\begin{aligned} {\widetilde{\beta }}:=\frac{\beta }{\Delta t}, \quad {\widetilde{\lambda }}:=\frac{\lambda }{\Delta t}, \text { and } {\widetilde{\alpha }}:=\frac{\alpha }{\Delta t}, \end{aligned}$$

where $\Delta t>0$ is some time step. Next we introduce the following rescaled time delay (representing the transmission time for neural signals between adjacent areas)

$$\begin{aligned} \tau := k \Delta t. \end{aligned}$$

Identifying $e_j^n$ as the approximation of some continuous fonction ${\textbf{e}}_j(t_n)$ at $t_n=n\Delta t$, we readily derive a delayed version of (20), namely

$$\begin{aligned} \frac{\textrm{d}}{\textrm{d}t}{} \textbf{e}_j(t){} & {} ={\widetilde{\beta }}\textbf{e}_{j-1}(t)-({\widetilde{\beta }}+{\widetilde{\lambda }})\textbf{e}_j(t)+{\widetilde{\alpha }}\textbf{e}_{j-1}(t-\tau )\\{} & {} \quad +{\widetilde{\lambda }}\textbf{e}_{j+1}(t-\tau )-{\widetilde{\alpha }}{} \textbf{e}_{j}(t-2\tau ), \quad t>0, \quad j\in {\mathbb {Z}}. \end{aligned}$$

In what follows, we first investigate the case of homogeneous oscillations, which are now possible because of the presence of time delays into the equation. Then, we turn our attention to oscillatory traveling waves.

#### Homogeneous Oscillations

One key difference of the above delayed equation compared to (20) is that spatially homogeneous solutions (i.e., solutions $\textbf{e}_j(t)$ that are independent of the layer *j*) may now have a non trivial dynamics, such as a broadly synchronized oscillation resembling brain rhythmic activity. Indeed, looking for solutions which are independent of *j*, we get the delayed ordinary differential equation

$$\begin{aligned} \frac{\textrm{d}}{\textrm{d}t}{} \textbf{e}(t)=-{\widetilde{\lambda }}\textbf{e}(t)+({\widetilde{\alpha }}+{\widetilde{\lambda }})\textbf{e}(t-\tau )-{\widetilde{\alpha }}{} \textbf{e}(t-2\tau ), \quad t>0. \end{aligned}$$

Looking for pure oscillatory exponential solutions $\textbf{e}(t)=e^{{\textbf{i}}\omega t}$ for some $\omega \in {\mathbb {R}}$ we obtain

$$\begin{aligned} {\textbf{i}}\omega =-{\widetilde{\lambda }}+({\widetilde{\alpha }}+{\widetilde{\lambda }})e^{-{\textbf{i}}\tau \omega }-{\widetilde{\alpha }}e^{-2{\textbf{i}}\tau \omega }. \end{aligned}$$

This leads to the system of equations

$$\begin{aligned} \left\{ \begin{aligned} 0&=-{\widetilde{\lambda }}+({\widetilde{\alpha }}+{\widetilde{\lambda }})\cos (\tau \omega )-{\widetilde{\alpha }}\cos (2\tau \omega ),\\ \omega&=-({\widetilde{\alpha }}+{\widetilde{\lambda }})\sin (\tau \omega )+{\widetilde{\alpha }}\sin (2\tau \omega ). \end{aligned} \right. \end{aligned}$$

Introducing $\varrho ={\widetilde{\lambda }}/{\widetilde{\alpha }}>0$, we observe that the above system writes instead

37 $$\begin{aligned} \left\{ \begin{aligned} 0&=-\varrho +(1+\varrho )\cos (\tau \omega )-\cos (2\tau \omega ),\\ \omega&={\widetilde{\alpha }} \left( -(1+\varrho )\sin (\tau \omega )+\sin (2\tau \omega )\right) . \end{aligned} \right. \end{aligned}$$

Using trigonometry identities, the first equation can be factorized as

$$\begin{aligned} 0=(1-\cos (\tau \omega ))(\varrho -1-2\cos (\tau \omega )). \end{aligned}$$

We distinguish several cases. If $\varrho >3$, then the above equation has solutions if and only if $\tau \omega =2k\pi$ for $k\in {\mathbb {Z}}$. Inspecting the second equation, we see that necessarily $k=0$ and $\omega =0$ is the only possible solution. When $\varrho =3$, we notice that the equation reduces to $0=(1-\cos (\tau \omega ))^2$, and the solutions are again given by $\tau \omega =2k\pi$ for $k\in {\mathbb {Z}}$, which yields $\omega =0$ because of the second equation. Now, if $\varrho \in (0,3)$, we deduce that either $\tau \omega =2k\pi$ for $k\in {\mathbb {Z}}$ or

$$\begin{aligned} \cos (\tau \omega )=\frac{\varrho -1}{2}. \end{aligned}$$

In the first case, we recover that $\omega =0$. Assuming now that $\omega \ne 0$, i.e., a true oscillation with non-zero frequency, we derive that

$$\begin{aligned} \tau \omega =\pm \textrm{arccos}\left( \frac{\varrho -1}{2}\right) +2k\pi , \quad k\in {\mathbb {Z}}. \end{aligned}$$

Injecting the above relation into the right-hand side of the second equation yields that

$$\begin{aligned} \omega ={\widetilde{\alpha }} \left( -(1+\varrho )\sin (\tau \omega )+\sin (2\tau \omega )\right) =\mp {\widetilde{\alpha }} \sqrt{(1+\varrho )(3-\varrho )}, \end{aligned}$$

and thus necessarily

$$\begin{aligned} (\tau ,\omega ) = \left( \frac{-\textrm{arccos}\left( \frac{\varrho -1}{2}\right) +2k\pi }{ {\widetilde{\alpha }} \sqrt{(1+\varrho )(3-\varrho )}},\pm {\widetilde{\alpha }} \sqrt{(1+\varrho )(3-\varrho )} \right) , \quad k\in {\mathbb {Z}}. \end{aligned}$$

**Fig. 27:**
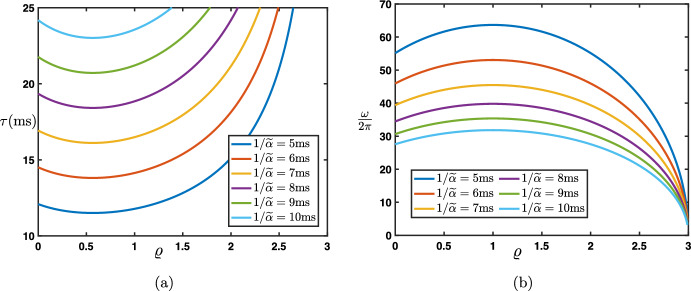
**a** Representation of the (minimal) time delay $\tau$ expressed in milliseconds as a function of the parameter $\varrho$ for various values of $1/{\widetilde{\alpha }}$ ranging from 5 to 10 ms. We observe that for values of $\varrho$ in the range (1/2, 1) the corresponding time delay $\tau$ takes values between 12 and 23 ms. **b** Representation of the frequency $\omega /2\pi$ (in Hertz) as a function of the parameter $\varrho$ for various values of $1/{\widetilde{\alpha }}$ ranging from 5 to 10 ms. We observe that for values of $\varrho$ in the range (1/2, 1) the corresponding frequency $\omega /2\pi$ takes values between 30 and 60 Hz (Color figure online)

We recover the fact that the system (37) is invariant by $\omega \mapsto -\omega$. Since $\textrm{arccos}\left( \frac{\varrho -1}{2}\right) \in [0,\pi ]$, we deduce that the smallest positive $\tau$ is always achieved at $k=1$. We computed for several values of ${\widetilde{\alpha }}$ the corresponding values of $\tau$ and $\omega$ (for $k=1$) as a function of $\varrho$, which are presented in Fig. 27a and b. We observe that for values of $\varrho$ in the range (1/2, 1) the corresponding time delay $\tau$ takes values between 12 and 23 ms for values of $1/{\widetilde{\alpha }}$ ranging from 5 to 10 ms. Correspondingly, in the same range of values for $\varrho$, the frequency $\omega /2\pi$ takes values between 30 and 60 Hz.

This tells us that, when the time delay $\tau$ is chosen to be around 10–20 ms, compatible with **biological** values for communication delays between adjacent cortical areas, and when hyperparameters ${\widetilde{\alpha }}$ and ${\widetilde{\lambda }}$ are suitably chosen (${\widetilde{\alpha }}$ in particular must be strong enough to allow rapid feed-forward error correction updates, i.e., around $1/{\widetilde{\alpha }}<8ms$, while ${\widetilde{\lambda }}$ can be chosen more liberally, as long as it stays $<3{\widetilde{\alpha }}$), then the network produces globally synchronized oscillations, comparable to experimentally observed brain rhythms in the $\gamma$-band regime (30–60 Hz). In this context, it is interesting to note that theoretical and neuroscientific considerations have suggested that error correction in predictive coding systems is likely to be accompanied by oscillatory neural activity around this same $\gamma$-frequency regime (Bastos et al. 2012).

Of course, a globally-synchronized gamma oscillation across all layers is a mathematical abstraction that has no equivalent in the brain. In electrophysiological experiments, gamma-band activity is typically found to be locally generated; according to the Communication-Through-Coherence (CTC) theory (Fries 2015), gamma oscillations can sometimes synchronize their phase across two or more successive processing stages. Our observations provide theoretical insight about the parameter range that would enable the emergence of between-layer gamma synchronization in neural network models, compatible with CTC.

#### Oscillatory Traveling Waves

However, experimental and computational studies have also suggested that oscillatory signatures of predictive coding could be found at lower frequencies, in the so-called $\alpha$-band regime, around 7–15 Hz. Furthermore, these oscillations are typically not homogeneous over space, as assumed in the previous section, but behave as forward- or backward-travelling waves with systematic phase shifts between layers (Alamia and VanRullen 2019). To explore this idea further, we now investigate the possibility of having traveling wave solutions of the form

$$\begin{aligned} {\textbf{e}}_j(t)=e^{{\textbf{i}}(\omega t +j\theta )}, \quad t>0, \quad j\in {\mathbb {Z}}, \end{aligned}$$

for some $\omega \in {\mathbb {R}}$ (representing the wave’s temporal frequency) and $\theta \in [0,2\pi )$ (representing the wave’s spatial frequency, i.e., its phase shift across layers), and we are especially interested in deriving conditions under which one can ensure that $\theta \ne 0$ (since otherwise, we would be again facing the homogeneous oscillation case). We only focus on the case ${\widetilde{\beta }}=0$ (as postulated, e.g., in Rao and Ballard’s (1999) work) and leave the case ${\widetilde{\beta }}>0$ for future investigations. As a consequence, the equation reduces to

$$\begin{aligned} \frac{\textrm{d}}{\textrm{d}t}{} \textbf{e}_j(t)=-{\widetilde{\lambda }}\textbf{e}_j(t)+{\widetilde{\alpha }}\textbf{e}_{j-1}(t-\tau )+{\widetilde{\lambda }}\textbf{e}_{j+1}(t-\tau )-{\widetilde{\alpha }}{} \textbf{e}_{j}(t-2\tau ), \quad t>0, \quad j\in {\mathbb {Z}}. \end{aligned}$$

Plugging in the ansatz $\textbf{e}_j(t)=e^{{\textbf{i}}\left( \omega t+j \theta \right) }$, we obtain:

$$\begin{aligned} {\textbf{i}}\omega = {\widetilde{\alpha }} \left( e^{-{\textbf{i}}(\omega \tau +\theta )}-e^{-2{\textbf{i}}\omega \tau } \right) +{\widetilde{\lambda }} \left( e^{-{\textbf{i}}(\omega \tau -\theta )}-1 \right) . \end{aligned}$$

Taking real and imaginary parts, we obtain the system

$$\begin{aligned} \left\{ \begin{aligned} 0&={\widetilde{\alpha }} \left( \cos (\omega \tau +\theta )-\cos (2\omega \tau ) \right) +{\widetilde{\lambda }} \left( \cos (\omega \tau -\theta )-1 \right) ,\\ \omega&=-{\widetilde{\alpha }} \left( \sin (\omega \tau +\theta )-\sin (2\omega \tau ) \right) -{\widetilde{\lambda }} \sin (\omega \tau -\theta ). \end{aligned}\right. \end{aligned}$$

Once again, we introduce $\varrho :=\frac{{\widetilde{\lambda }}}{{\widetilde{\alpha }}}\ge 0$ where we implicitly assumed that we always work in the regime ${\widetilde{\alpha }}>0$. Then, we note that the right-hand side of the first equation of the above system can be factored as

$$\begin{aligned}{} & {} {\widetilde{\alpha }} \left( \cos (\omega \tau +\theta )-\cos (2\omega \tau ) \right) +{\widetilde{\lambda }} \left( \cos (\omega \tau -\theta )-1 \right) =-2{\widetilde{\alpha }}\sin \left( \frac{\theta -\omega \tau }{2}\right) \\{} & {} \quad \left( \varrho \sin \left( \frac{\theta -\omega \tau }{2}\right) +\sin \left( \frac{\theta +3\omega \tau }{2}\right) \right) . \end{aligned}$$

As a consequence, either $\sin \left( \frac{\theta -\omega \tau }{2}\right) =0$, that is $\omega \tau =\theta +2k\pi$ for $k\in {\mathbb {Z}}$, which then leads, from the second equation, to $\omega =0$ and $\theta =0$ since we restrict $\theta \in [0,2\pi )$, or $\sin \left( \frac{\theta -\omega \tau }{2}\right) \ne 0$. In the latter case, assuming that $\omega \tau \ne \theta +2k\pi$ for $k\in {\mathbb {Z}}$, we get that

$$\begin{aligned} 0=\varrho \sin \left( \frac{\theta -\omega \tau }{2}\right) +\sin \left( \frac{\theta +3\omega \tau }{2}\right) . \end{aligned}$$

We will now study several cases.

**Fig. 28:**
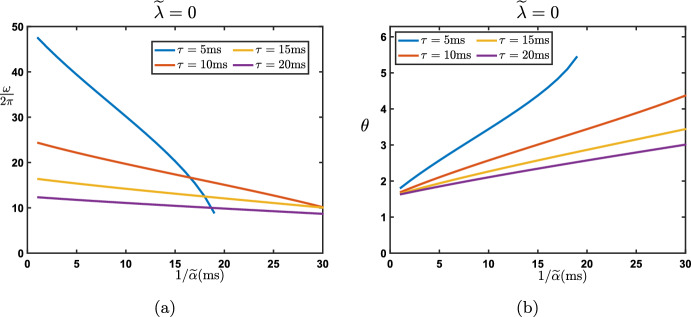
Representation of the temporal frequency $\omega /2\pi$ (in Hz) and the spatial frequency $\theta \in [0,2\pi )$ (panel (**a**) and (**b**) respectively) in the case ${\widetilde{\lambda }}=0$ as a function of $1/{\widetilde{\alpha }}$ (in ms) for several values of the time delay $\tau$ (Color figure online)

**Case** $\varrho =0$. (In other words, this case implies ${\widetilde{\lambda }}=0$, that is, a system with no feedback error correction.)

From $\sin \left( \frac{\theta +3\omega \tau }{2}\right) =0$, we deduce that $\theta =-3\omega \tau +2k\pi$ for some $k\in {\mathbb {Z}}$, and reporting into the second equation of the system, we end up with

$$\begin{aligned} \omega =2{\widetilde{\alpha }} \sin (2\omega \tau ). \end{aligned}$$

We always have the trivial solution $\omega =0$ with $\theta =0$. In fact, when $4{\widetilde{\alpha }}\tau \le 1$, $\omega =0$ is the only solution of the above equation. On the other hand, when $4{\widetilde{\alpha }}\tau >1$, there can be multiple non trivial solutions. At least, for each $({\widetilde{\alpha }},\tau )$ such that $4{\widetilde{\alpha }}\tau >1$ there always exist a unique $\omega _c({\widetilde{\alpha }},\tau )\in \left( 0, \frac{\pi }{2\tau }\right)$ solution of the above equation. This gives a corresponding $\theta _c^k=-3\omega _c({\widetilde{\alpha }},\tau )\tau +2k\pi$ with $k\in {\mathbb {Z}}$, and retaining the corresponding value of $\theta$ in the interval $[0,2\pi )$, we have $\theta _c=-3\omega _c({\widetilde{\alpha }},\tau )\tau +2\pi$. We refer to Fig. 28 for an illustration of the solutions $(\omega ,\theta )$ for several values of the parameters.

Interestingly, we see that for values of the time delay $\tau$ between 10 and 20 ms, consistent with biological data, the observed oscillation frequency is lower than in the previous case, and now compatible with the $\alpha$-frequency regime (between 10 and 20 Hz). Furthermore, the phase shift between layers $\theta$ varies roughly between 2 and 4 radians. As phase shifts below $\pi$ or above $\pi$ radians indicate respectively backward- or forward-travelling waves, we see that the exact value of the parameters $\tau$ and ${\widetilde{\alpha }}$ critically determines the propagation direction of the travelling waves: stronger feedforward error correction (lower values of $1/{\widetilde{\alpha }}$) and longer communication delays $\tau$ will tend to favor backward-travelling waves; and vice-versa, weaker feedforward error correction (higher values of $1/{\widetilde{\alpha }}$) and shorter communication delays $\tau$ will favor forward-travelling waves.

**Case** $\varrho =1$. Now we assume that ${\widetilde{\lambda }}\ne 0$, that is, the system now includes feedback error correction. At first, we consider the simpler case when $\varrho =1$, that is when ${\widetilde{\alpha }}={\widetilde{\lambda }}$, where the equation can also be solved easily. Indeed, we have either

$$\begin{aligned} \frac{\omega \tau -\theta }{2}=\frac{\theta +3\omega \tau }{2}+2k\pi , \quad k\in {\mathbb {Z}}, \end{aligned}$$

or

$$\begin{aligned} \frac{\omega \tau -\theta }{2}=\pi -\frac{\theta +3\omega \tau }{2}+2k\pi , \quad k\in {\mathbb {Z}}. \end{aligned}$$

This equivalent to

$$\begin{aligned} \theta =-\omega \tau +2k\pi , \quad k\in {\mathbb {Z}}, \end{aligned}$$

or

$$\begin{aligned} \omega \tau =\frac{\pi }{2}+k\pi , \quad k\in {\mathbb {Z}}. \end{aligned}$$

Let assume first that $\theta =-\omega \tau +2k\pi$ for some $k\in {\mathbb {Z}}$, then the second equation of the system gives $\omega =0$ since ${\widetilde{\alpha }}={\widetilde{\lambda }}$ when $\varrho =1$, and thus we end up with $\theta =0$. Now, if $\omega \tau =\frac{\pi }{2}+k\pi$ for some $k\in {\mathbb {Z}}$, the second equation leads to

$$\begin{aligned} \omega =-2{\widetilde{\alpha }}\cos (\theta +k\pi ), \end{aligned}$$

from which we deduce that necessarily we must have

$$\begin{aligned} \frac{(2k+1)\pi }{-4{\widetilde{\alpha }}\tau }=\cos (\theta +k\pi ), \quad k\in {\mathbb {Z}}. \end{aligned}$$

We first remark that if $4{\widetilde{\alpha }}\tau <\pi$, then the above equation has no solution. On the other hand if $4{\widetilde{\alpha }}\tau \ge \pi$, one can obtain solutions to the above equation. Indeed, let us denote

$$\begin{aligned} p:=\left\lfloor \frac{4{\widetilde{\alpha }}\tau }{\pi } \right\rfloor \ge 1 \end{aligned}$$

the integer part of $\frac{4{\widetilde{\alpha }}\tau }{\pi }$. Then, for each $k\in {\mathbb {Z}}$ such that $|2k+1|\le p$, we have

$$\begin{aligned} \theta =\pm \textrm{arccos}\left( (-1)^{k+1} \frac{(2k+1)\pi }{4{\widetilde{\alpha }}\tau }\right) +2m\pi , \quad m\in {\mathbb {Z}}, \end{aligned}$$

with corresponding $\omega$ given by

$$\begin{aligned} \omega =\frac{\pi }{2\tau }+\frac{k\pi }{\tau }. \end{aligned}$$

That is, the set of solutions is given by

$$\begin{aligned} (\omega ,\theta )=\left( \frac{\pi }{2\tau }+\frac{k\pi }{\tau },\pm \textrm{arccos}\left( (-1)^{k+1} \frac{(2k+1)\pi }{4{\widetilde{\alpha }}\tau }\right) +2m\pi \right) , \end{aligned}$$

for each $k\in {\mathbb {Z}}$ such that $|2k+1|\le \left\lfloor \frac{4{\widetilde{\alpha }}\tau }{\pi } \right\rfloor$ and $m\in {\mathbb {Z}}$. If we only retain the smallest $\omega$ and the corresponding value of $\theta \in [0,2\pi )$, we must take $k=0$, and we have two solutions

$$\begin{aligned}{} & {} (\omega ,\theta )=\left( \frac{\pi }{2\tau }, \textrm{arccos}\left( -\frac{\pi }{4{\widetilde{\alpha }}\tau }\right) \right) , \text { and } (\omega ,\theta )=\left( \frac{\pi }{2\tau }, -\textrm{arccos}\left( -\frac{\pi }{4{\widetilde{\alpha }}\tau }\right) +2\pi \right) \\{} & {} \quad \text { if } 4{\widetilde{\alpha }}\tau \ge \pi . \end{aligned}$$

We note that in this case the temporal frequency only depends on the time delay $\tau$ since it is given by $\frac{\omega }{2\pi }=\frac{1}{4\tau }$ and ranges from 12.5 Hz ($\alpha$-band regime) to 25 Hz ($\beta$-band regime) for values of $\tau$ between 10 and 20 ms (as long as ${\widetilde{\alpha }}$ is fixed such that $4{\widetilde{\alpha }}\tau \ge \pi$ is verified). The corresponding spatial frequencies are $\textrm{arccos}\left( -\frac{\pi }{4{\widetilde{\alpha }}\tau }\right) \in (\pi /2,\pi )$ and $-\textrm{arccos}\left( -\frac{\pi }{4{\widetilde{\alpha }}\tau }\right) +2\pi \in (3\pi /2,2\pi )$.

In summary, when the feedforward and feedback error correction strengths are matched (that is when ${\widetilde{\alpha }}={\widetilde{\lambda }}$) and sufficiently high (such that $4{\widetilde{\alpha }}\tau \ge \pi$), then the system will show two simultaneous travelling waves at the same frequency in the $\alpha$-band or $\beta$-band regime, but travelling in opposite directions, one as a feedforward wave and the other as a feedback wave.

**Case** $\varrho >1$. Here, the feedback error correction $\lambda$ is stronger than the feedforward $\alpha$. In this case, we remark that

$$\begin{aligned}{} & {} \varrho \sin \left( \frac{\theta -\omega \tau }{2}\right) +\sin \left( \frac{\theta +3\omega \tau }{2}\right) =\left( \varrho +\cos (2\omega \tau )\right) \sin \left( \frac{\theta -\omega \tau }{2}\right) \\{} & {} \quad +\cos \left( \frac{\theta -\omega \tau }{2}\right) \sin (2\omega \tau ). \end{aligned}$$

Since $\varrho >1$ and $\omega \tau \ne \theta +2k\pi$ for $k\in {\mathbb {Z}}$, we have $\left( \varrho +\cos (2\omega \tau )\right) \sin \left( \frac{\theta -\omega \tau }{2}\right) \ne 0$, and thus $\cos \left( \frac{\theta -\omega \tau }{2}\right) \sin (2\omega \tau )\ne 0$ otherwise we would reach a contradiction since we try to solve

$$\begin{aligned} \varrho \sin \left( \frac{\theta -\omega \tau }{2}\right) +\sin \left( \frac{\theta +3\omega \tau }{2}\right) =0. \end{aligned}$$

As a consequence, $\cos \left( \frac{\theta -\omega \tau }{2}\right) \ne 0$ and we can rewrite the above equation as

$$\begin{aligned} \tan \left( \frac{\theta -\omega \tau }{2}\right) =-\frac{\sin (2\omega \tau )}{\varrho +\cos (2\omega \tau )}, \end{aligned}$$

so that

$$\begin{aligned} \theta =\omega \tau -2\textrm{arctan}\left( \frac{\sin (2\omega \tau )}{\varrho +\cos (2\omega \tau )}\right) +2k\pi , \quad k\in {\mathbb {Z}}. \end{aligned}$$

Injecting this expression for $\theta$ into the second equation, we find, after simplification, that

$$\begin{aligned} \omega ={\widetilde{\alpha }}\frac{2\sin (2\omega \tau )\left( 1-\varrho ^2\right) }{2\varrho \cos (2\omega \tau )+\varrho ^2+1}. \end{aligned}$$

We first remark that $\omega =0$ is always a solution, giving $\theta =0$. Now, inspecting the right-hand of the above expression, we get that

$$\begin{aligned} \frac{2{\widetilde{\alpha }}\left( 1-\varrho ^2\right) }{2\varrho \cos (2\omega \tau )+\varrho ^2+1}<0, \text { for all } \omega \in {\mathbb {R}}. \end{aligned}$$

As a consequence, we look for the negative minima of the function $\omega \mapsto \frac{\sin (2\omega \tau )}{2\varrho \cos (2\omega \tau )+\varrho ^2+1}$ which are given by $\omega _0=\frac{\pi }{2\tau }+\frac{1}{2\tau }\textrm{arccos}\left( \frac{2\varrho }{1+\varrho ^2}\right) +\frac{k\pi }{\tau }$ for $k\in {\mathbb {Z}}$, at such minima, one gets that

$$\begin{aligned} \frac{\sin (2\omega _0\tau )}{2\varrho \cos (2\omega _0\tau )+\varrho ^2+1}=\frac{1}{1-\varrho ^2}. \end{aligned}$$

This implies that if $4{\widetilde{\alpha }}\tau <\pi +\textrm{arccos}\left( \frac{2\varrho }{1+\varrho ^2}\right)$, then there is no other solution than $(\omega ,\theta )=(0,0)$. As a consequence, one needs to assume $4{\widetilde{\alpha }}\tau \ge \pi +\textrm{arccos}\left( \frac{2\varrho }{1+\varrho ^2}\right)$ to ensure the existence of at least one non trivial solution. We remark that this condition is consistent with our condition $4{\widetilde{\alpha }}\tau \ge \pi$ derived in the case $\varrho =1$.

**Case** $0<\varrho <1$. We start once again from the equation

$$\begin{aligned} 0=\left( \varrho +\cos (2\omega \tau )\right) \sin \left( \frac{\theta -\omega \tau }{2}\right) +\cos \left( \frac{\theta -\omega \tau }{2}\right) \sin (2\omega \tau ). \end{aligned}$$

This time, it is possible that $\varrho +\cos (2\omega \tau )=0$, which gives necessarily that

$$\begin{aligned} \omega \tau =\pm \frac{1}{2}\textrm{arccos}(-\varrho )+k\pi , \quad k\in {\mathbb {Z}}. \end{aligned}$$

But if $\varrho +\cos (2\omega \tau )=0$, then one has $0=\cos \left( \frac{\theta -\omega \tau }{2}\right) \sin (2\omega \tau )$.

Let us first assume that $\cos \left( \frac{\theta -\omega \tau }{2}\right) =0$, such that $\theta =\omega \tau +(2k+1)\pi$ for $k\in {\mathbb {Z}}$. Now, looking at the second equation, we find that

$$\begin{aligned} \omega = 2{\widetilde{\alpha }}\sin (2\omega \tau )=\pm 2{\widetilde{\alpha }}\sqrt{1-\varrho ^2}, \end{aligned}$$

which implies that it is possible only if

$$\begin{aligned} \tau = \frac{\textrm{arccos}(-\varrho )+k\pi }{4{\widetilde{\alpha }}\sqrt{1-\varrho ^2}}, \quad k\ge 0. \end{aligned}$$

As a conclusion, if $\tau$ and $0<\varrho <1$ satisfy $\tau = \frac{\textrm{arccos}(-\varrho )+k\pi }{4{\widetilde{\alpha }}\sqrt{1-\varrho ^2}}$ for some positive integer $k\ge 0$, then

$$\begin{aligned}{} & {} (\omega ,\theta )=\left( 2{\widetilde{\alpha }}\sqrt{1-\varrho ^2},\frac{1}{2}\textrm{arccos}(-\varrho )-\pi \right) , \text { and } \\{} & {} (\omega ,\theta )=\left( -2{\widetilde{\alpha }}\sqrt{1-\varrho ^2},-\frac{1}{2}\textrm{arccos}(-\varrho )+\pi \right) , \end{aligned}$$

are corresponding solutions of the problem.

Next, let us assume that $\sin (2\omega \tau )=0$, implying that $2\omega \tau =k\pi$ for $k\in {\mathbb {Z}}$. Now we readily remark that since $0<\varrho <1$, we have $\textrm{arccos}(-\varrho )\in (\pi /2,\pi )$. As a consequence, we should have

$$\begin{aligned} \pm \textrm{arccos}(-\varrho )+2k\pi = p\pi , \quad k,p\in {\mathbb {Z}}, \end{aligned}$$

this is impossible and thus $\sin (2\omega \tau )\ne 0$ and we are back to the case treated before.

We now assume that $\varrho +\cos (2\omega \tau )\ne 0$. In that case, we can proceed as in the case $\varrho >1$ and obtain that

$$\begin{aligned} \theta =\omega \tau -2\textrm{arctan}\left( \frac{\sin (2\omega \tau )}{\varrho +\cos (2\omega \tau )}\right) +2k\pi , \quad k\in {\mathbb {Z}}, \end{aligned}$$

which gives

$$\begin{aligned} \omega ={\widetilde{\alpha }}\frac{2\sin (2\omega \tau )\left( 1-\varrho ^2\right) }{2\varrho \cos (2\omega \tau )+\varrho ^2+1}. \end{aligned}$$

Once again, $(\omega ,\theta )=(0,0)$ is always a solution. What changes in this case is that now

$$\begin{aligned} \frac{2{\widetilde{\alpha }}\left( 1-\varrho ^2\right) }{2\varrho \cos (2\omega \tau )+\varrho ^2+1}>0, \text { for all } \omega \in {\mathbb {R}}. \end{aligned}$$

This time, one needs to look at the positive maxima of the map $\omega \mapsto \frac{\sin (2\omega \tau )}{2\varrho \cos (2\omega \tau )+\varrho ^2+1}$ which are given by $\omega _0=\frac{\pi }{2\tau }-\frac{1}{2\tau }\textrm{arccos}\left( \frac{2\varrho }{1+\varrho ^2}\right) +\frac{k\pi }{\tau }$ for $k\in {\mathbb {Z}}$, at such maxima, one gets that

$$\begin{aligned} \frac{\sin (2\omega _0\tau )}{2\varrho \cos (2\omega _0\tau )+\varrho ^2+1}=\frac{1}{1-\varrho ^2}. \end{aligned}$$

As a consequence, if $4{\widetilde{\alpha }}\tau <\pi -\textrm{arccos}\left( \frac{2\varrho }{1+\varrho ^2}\right)$, then there is no other solution than $(\omega ,\theta )=(0,0)$. To obtain at least one non trivial positive solution, one needs to impose that $4{\widetilde{\alpha }}\tau \ge \pi -\textrm{arccos}\left( \frac{2\varrho }{1+\varrho ^2}\right)$. Once again, this condition is consistent with the condition $4{\widetilde{\alpha }}\tau \ge \pi$ derived in the case $\varrho =1$. We can also derive a second simple condition which ensures the existence of a non trivial solution by looking at the behavior near $\omega \sim 0$ where we have

$$\begin{aligned} {\widetilde{\alpha }}\frac{2\sin (2\omega \tau )\left( 1-\varrho ^2\right) }{2\varrho \cos (2\omega \tau )+\varrho ^2+1}\sim \frac{4{\widetilde{\alpha }}\tau \left( 1-\varrho \right) }{1+\varrho }\omega . \end{aligned}$$

Thus if

$$\begin{aligned} 4{\widetilde{\alpha }}\tau >\frac{1+\varrho }{1-\varrho }, \end{aligned}$$

then there exists at least one positive solution $\omega \in (0,\pi /2\tau )$ to the above equation (and also one negative solution in $(-\pi /2\tau ,0)$ by symmetry). Note that the condition $4{\widetilde{\alpha }}\tau >\frac{1+\varrho }{1-\varrho }$ is consistent with the condition $4{\widetilde{\alpha }}\tau >1$ derived in the case $\varrho =0$.

**Fig. 29:**
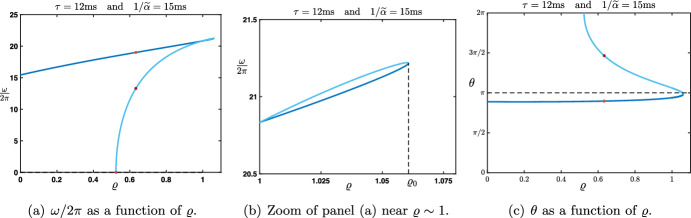
Representation of the temporal frequency $\omega /2\pi$ (in Hz) and the spatial frequency $\theta \in [0,2\pi )$ (panel (**a**) and (**c**) respectively) as a function of $\varrho$ for fixed values of the time delay $\tau =12$ ms and $1/{\widetilde{\alpha }}=15$ ms. Panel (**b**) represents a zoom of panel (**a**) near $\varrho \sim 1$ where the two branches terminate (Color figure online)

**Examples** To illustrate the different scenarios and their possible interpretations in terms of brain oscillations, we take here two distinct examples corresponding to the situations described above. We report in Fig. 29 the non trivial branches of solutions corresponding to positive values of $\omega$, as a function of $\varrho$ for fixed values of the time delay $\tau =12$ ms and $1/{\widetilde{\alpha }}=15$ ms. These values are biologically plausible and correspond to the values used in Alamia and VanRullen (2019). Upon denoting

$$\begin{aligned} \varrho _c:=\frac{1-4{\widetilde{\alpha }}\tau }{1+4{\widetilde{\alpha }}\tau }\in (0,1), \end{aligned}$$

for all $\varrho \in [0,\varrho _c)$, we get the existence of a unique branch of solution (blue curve) for the temporal frequency $\omega /2\pi$. A second branch (light blue curve) of solutions emerges precisely at $\varrho =\varrho _c$. These two branches cross at $\varrho =1$ where $\omega /2\pi =\frac{1}{4\tau }$ and terminate at a value of $\varrho =\varrho _0\sim 1.06$ (see Fig. 29b). The branch of solutions which exists for all values of $\varrho \in [0,\varrho _0]$ has an associated spatial frequency which is almost constant and whose value is around $\sim 2.82\in (0,\pi )$. On the other hand, the branch of solutions which only exists for values of $\varrho \in (\varrho _c,\varrho _0]$ has an associated spatial frequency which lies in $(\pi ,2\pi )$. Let us remark that at $\varrho =1$, the spatial frequencies of the two solutions are different and symmetric with respect to $\pi$. Furthermore, at $\varrho =\varrho _0\sim 1.06$ where the two branches collide the associated spatial frequency is $\theta \sim \pi$. Let us finally note that for $\varrho \in [1,\varrho _0]$, the spatial frequencies of the two branches are almost identical, although the secondary branch is slightly above the primary one.

**Fig. 30:**
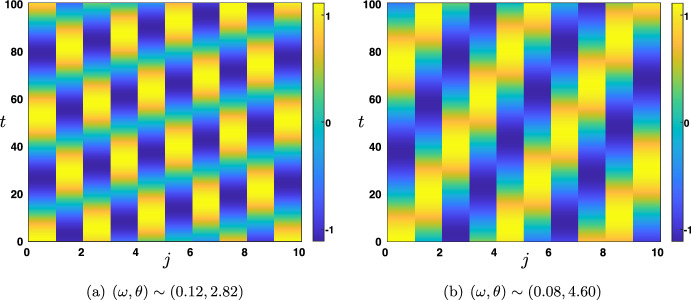
Space-time plot of $\cos (\omega t+\theta j)$ for values of $(\omega ,\theta )$ which correspond to the orange and dark red points of Fig. 29 lying respectively on the blue and light blue curves, time *t* is in ms. In (**a**), the temporal frequency is $\omega /2\pi \sim 19$ Hz while in (**b**) it is $\omega /2\pi \sim 13.3$ Hz. In (**a**), since $\theta \in (0,\pi )$, we observe a backward propagation of the wave while in (**b**) we have a forward propagation since $\theta \in (\pi ,2\pi )$ (Color figure online)

Correspondingly we illustrate in Fig. 30, the space-time plot for two points along the two different branches which correspond to the orange and dark red points in Fig. 29. The corresponding values are $(\omega ,\theta )\sim (0.12,2.82)$ and $(\omega ,\theta )\sim (0.08,4.60)$ and associated to the same value of $\varrho \sim 0.633$. In the first panel of Fig. 30a, which corresponds to the point on the branch of solution defined for all $\varrho \in [0,\varrho _0]$, since the corresponding value of the spatial frequency is $\theta \in (0,\pi )$, we observe an apparent backward propagation, while in the second panel of Fig. 30b, we observe a forward propagation. This corresponds to the point on the lower branch of the solutions defined for values of $\varrho \in (\varrho _c,\varrho _0]$ with associated spatial frequency $\theta \in (\pi ,2\pi )$. From a biological point of view, this indicates that the more interesting range of the parameters is the one with $\varrho \in (\varrho _c,\varrho _0]$ and the corresponding branch of solutions which emerges at $\varrho =\varrho _c$ from the trivial solution $(\omega ,\theta )\sim (0,0)$ since in this case we obtain an oscillatory traveling wave with forward propagation into the network.

**Fig. 31:**
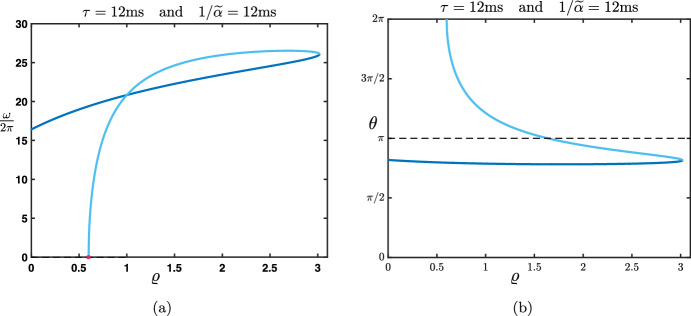
Representation of the temporal frequency $\omega /2\pi$ (in Hz) and the spatial frequency $\theta \in [0,2\pi )$ (panel (**a**) and (**b**) respectively) as a function of $\varrho \in [0,1]$ for fixed values of the time delay $\tau =12$ ms and $1/{\widetilde{\alpha }}=12$ ms (Color figure online)

In Fig. 31, we show the global structure of the branches for a second example, with fixed values of the time delay $\tau =12$ ms and $1/{\widetilde{\alpha }}=12$ ms, which are still biologically relevant values. We observe that the two branches terminate at a value of $\varrho =\varrho _0\sim 3.03$ with a crossing at $\varrho =1$. For $\varrho \in [1,\varrho _0]$, the primary branch (blue curve) has a temporal frequency below the secondary branch (light blue curve), the difference in frequencies is almost 5 Hz for values of $\varrho \sim 2$. Even more interestingly, we see that the corresponding spatial frequencies along the secondary branch are decreasing from $2\pi$ to a final value below $\pi$ at $\varrho _0$ indicating that by increasing the value of $\varrho$ we can reverse the direction of propagation from forward to backward oscillatory traveling waves. The transition occurs for $\varrho \sim 1.65$, that is for values of $1/{\widetilde{\lambda }}\sim$7–8 ms. It is further noticed that the associated temporal frequencies in the backward regime are around 25 Hz ($\beta$-frequency regime) much higher than for forward traveling waves whose temporal frequencies range from 0 to 20 Hz (and include the $\alpha$-frequency regime).

**Summary** In this section, we saw that including temporal delays in the time-continuous version of the system produces non-trivial dynamics that can be characterized analytically. Contrary to the discrete version of the system, which can only be analyzed for a few discrete time delays $k=1,2,...$, the continuous version is informative for a wide range of delays, compatible with biological data, and the resulting frequencies are very diverse. In particular, we observed homogenous synchronized oscillations in the gamma band (30–60 Hz) that emerged when the feed-forward error correction term ${\widetilde{\alpha }}$ was strong enough (roughly, with $1/{\widetilde{\alpha }} < 8 ms$). But we also found situations in which the oscillatory activity was not homogenous, but propagated as a travelling wave through the network. With biologically plausible values for the various parameters, the waves could propagate forward in the alpha-band (7–15 Hz) frequency range, and when the feedback error correction term ${\widetilde{\lambda }}$ was strong enough (e.g., $1/{\widetilde{\lambda }}<8$ ms while $1/{\widetilde{\alpha }}=12$ ms), they started moving backward at a faster frequency in the beta-band (15–30 Hz). Altogether, this pattern of results is compatible with various (sometimes conflicting) observations from the Neuroscience literature (Alamia and VanRullen 2019; Bastos et al. 2012), and informs us about the conditions in which the corresponding dynamic behaviors might emerge in such bio-inspired neural networks with predictive coding dynamics incorporating communication delays.

## Discussion

### Contributions

We proposed a mathematical framework to explore the properties and stability of neural network models of the visual system comprising a hierarchy of visual processing areas (or “layers”), mutually connected according to the principles of predictive coding. Using a discrete model, as is typically done in the recent deep learning literature, we introduced the amplification factor function, which serves to characterize the interesting (i.e., “marginally stable”) regions as a function of the model hyperparameters. When considered on an infinite domain, we showed that the response of our linear neural network to a Dirac delta initialization presents a universal behavior given by a Gaussian profile with fixed variance and which spreads at a given speed. Both speed and variance could be explicitly characterized in terms of the model hyperparameters. This universal Gaussian profile was then the key to understand the long-time dynamics of the linear neural network set on a semi-infinite domain with a fixed constant source term at the left boundary of the network.

At first, we ignored the influence of neuronal selectivity and used feed-forward and feedback connection matrices set to the identity matrix. When $\beta =0$ (no feedforward update after the network initialization), we observed that hyperparameters $\alpha$ and $\lambda$ compete for forward and backward propagation, respectively. When $\beta >0$, the constant feedforward input makes things more complex, with $\lambda$ (feedback error correction) now competing with $\beta +\alpha$ (feedforward drive and feedforward error correction). In the special case when $\alpha +\lambda =1$, a second (but spurious) mode of propagation with rapidly alternating activity can emerge, whose direction is determined by the competition between $\alpha$ and $\beta +\lambda$.

Next, to evaluate the influence of a more complex and functionally relevant connectivity matrix, we defined *neural assemblies* reflecting the eigenvectors of the matrix. Each of these neural assemblies can be analyzed separately, and its behavior depends on the corresponding eigenvalue (in addition to the hyperparameters $\alpha$, $\beta$ and $\lambda$, as explained above). Different assemblies can simultaneously support different dynamics, so that some may propagate information forward, others may not propagate at all (acting as a filter on the inputs), while yet others might propagate backward (e.g., carrying “priors” set by preceding activations). We again saw a number of cases where “fringe” or spurious behavior arose, e.g., rapid alternations in activity, and understood that this could be caused by the discrete nature of our model, when the time steps defining the model’s temporal resolution are too coarse.

The time-continuous version of the model helped us overcome this issue, and characterize dynamics in the limit of infinitely small time steps. The amplification factor function is still crucial in this situation, but it produces more robust results, without fringe behavior or spurious oscillations. In particular, the analysis of stability and propagation direction/speed was greatly simplified in this continuous case.

The same time-continuous model also allowed us to investigate the inclusion of communication delays between layers. In this case, we demonstrated the emergence of genuine oscillatory dynamics and travelling waves in various frequency bands compatible with neuroscientific observations (alpha-band from 7 to 15 Hz, beta-band from 15 to 30 Hz and gamma-band from 30 to 60 Hz).

Finally, we considered fully continuous versions of the model, not only in time but also in space, both across network depth (across neuronal layers) and width (across neurons in the same layer). This mathematical abstraction revealed that our model could be understood as a transport equation, and that it produced diffusion dynamics.

### Biological Interpretations

The mathematical framework that we proposed naturally lends itself to interpretation in biological terms. The model’s hyperparameters reflect the strength of feedforward and feedback signalling in the brain. These are determined not only by axonal density and synaptic strength (that vary slowly throughout development and learning), but can also be gated by other brain regions and control systems, e.g., through the influence of neurotransmitters, and thus vary much more dynamically. For instance, the feedforward drive $\beta$ could be more active to capture sensory information immediately after each eye movement, and decrease over time until the next eye movement (Knoell et al. 2011); similarly, feedback error correction $\lambda$ could dominate over the feedforward error correction $\alpha$ for one given second (e.g., because top-down attention drives expectation signals) and decrease in the next second (e.g., because unexpected sensory inputs have been detected) (Tschantz et al. 2022). In this dynamic context, it is fundamental to be able to characterize the dependence of the system’s behavior on the exact hyperparameter values. Fortunately, our framework reveals that when the hyperparameters vary, the stability of the system, and its ability to propagate signals and maintain activity, change in predictable ways. Some hyperparameter combinations would not support signal propagation at all; others would render the system unstable, e.g., because of runaway excitation. Under the assumption that the brain behaves as a predictive coding system, our equations inform us about the parameter regimes compatible with this paradigm.

Using our time-continuous model, we found that predictive coding dynamics associated with inter-areal communication delays result in oscillatory activity. This finding resonates with both experimental observations and neuroscientific theories (Bastos et al. 2012; Alamia and VanRullen 2019).

Bastos et al. (2012, 2015) suggested that feedforward error correction could be accompanied by gamma-band oscillations; this suggestion was verified in our model, with synchronized gamma rhythms appearing when the corresponding hyperparameter ${\widetilde{\alpha }}$ was strong enough (and with a frequency that monotonically increased from 30 to 60 Hz when the value of $1/{\widetilde{\alpha }}$ decreased from 10 to 5 ms). However, considering that the communication delay $\tau$ between two adjacent brain regions is a fixed property of the system (a reasonable first approximation), our analysis shows that this oscillatory mode will only happen for a narrow range and a very precise combination of hyperparameter values ${\widetilde{\alpha }}$ and ${\widetilde{\lambda }}$ (see Fig. 27). Similarly in the brain, synchronized gamma-band oscillations between areas are sometimes observed during electrophysiological recordings (Bastos et al. 2015), and sometimes not (Ray and Maunsell 2015).

By relaxing the phase delay between layers, our equations also revealed the potential emergence of oscillatory travelling waves across the network, similar to those observed in human EEG experiments (Alamia and VanRullen 2019; Pang et al. 2020; Alamia et al. 2020, 2023). Again, for a fixed communication delay $\tau$, these waves may only happen for specific values and combinations of the hyperparameters ${\widetilde{\alpha }}$ and ${\widetilde{\lambda }}$. In certain regimes (see e.g., Fig. 31 with $1/{\widetilde{\alpha }}=1/{\widetilde{\lambda }}=12$ ms), two waves might coexist at the same frequency, but going in opposite directions. This matches experimental reports of co-occurring feedforward and feedback waves in the brain (Alamia et al. 2020, 2023). Upon increasing the feedback strength ${\widetilde{\lambda }}$, we saw that an initial alpha-band (7–15 Hz) feed-forward wave could accelerate (towards the beta-band, 15–30 Hz) and eventually reverse its direction, producing a feedback wave. Similar reversal phenomena have also been reported for oscillatory waves in the human brain (Pang et al. 2020; Alamia et al. 2020, 2023).

### Limitations and Future Extensions

“All models are wrong, but some are useful” (Box et al. 1979). Our model, like all mathematical models, is based on simplifications, approximations and assumptions, and can only be valid under those assumptions. Some (if not all) of these assumptions are questionable, and future work will need to determine the robustness of the model, or its potential modifications, when relaxing these assumptions.

Even though we assumed that our bio-inspired neural network follows the general principles of predictive coding (Rao and Ballard 1999), our system’s hyperparameters can in fact be modulated to accommodate many variants of this framework (Wen et al. 2018; Choksi et al. 2021; Heeger 2017; Tschantz et al. 2022). One other important assumption that we made was to simplify the connectivity matrices between neuronal layers—which determines the selectivity of each neuron, and thus the functionality of the entire system. Even when we moved past the “identity” assumption, the connection matrices that we adopted were constrained to be symmetric, and most importantly, were assumed to be similar from one layer to the next. This made our equations tractable, but it constitutes a clear restriction, and a departure from practical applications of such bio-inspired deep neural networks that will need to be addressed in future extensions. As already emphasized in the introduction, another important limitation that we wish to relax in future works is the fact that we have considered a linear model although real biological networks or deep neural networks are intrinsically nonlinear. Going beyond the linear analysis that we have presented here would need the development of new theoretical techniques which constitutes a major open problem to be addressed in forthcoming works.

**Fig. 32:**
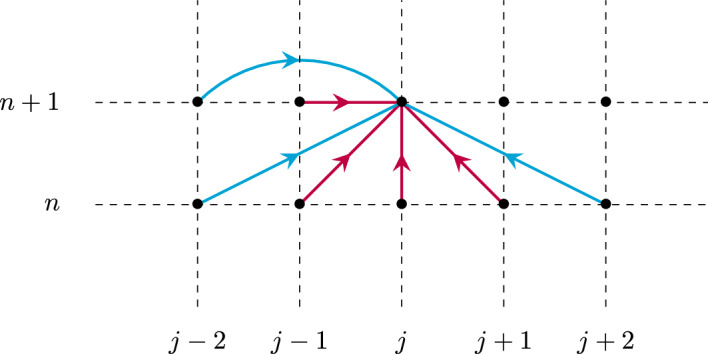
Illustration of the network structure of model (38) where blue arrows indicate the new long-range interactions coming from layer $j\pm 2$ (Color figure online)

Aside from exploring richer patterns of connectivity between adjacent layers, another natural extension of the model could be to incorporate long-range interactions, beyond the immediately adjacent layers. For instance, one could explore a simple second-order layer model (illustrated in Fig. 32), whose scalar version reads:

38 $$\begin{aligned} e_j^{n+1}-\beta _1e_{j-1}^{n+1}-\beta _2e_{j-2}^{n+1}= & {} \alpha _1e_{j-1}^{n}+\alpha _2e_{j-2}^{n}+(1-\beta ^*-\lambda ^*- \alpha ^* )e_j^{n}\nonumber \\{} & {} + \lambda _1 e_{j+1}^{n}+ \lambda _2 e_{j+2}^{n}, \quad j\in {\mathbb {Z}}, \end{aligned}$$

where we have set $\beta ^*:=\beta _1+\beta _2$, $\alpha ^*:=\alpha _1+\alpha _2$ and $\lambda ^*:=\lambda _1+\lambda _2$. Once again the fate of such a system (38) would be dictated by the amplification factor function

$$\begin{aligned} \rho (\theta )=\frac{\alpha _1 e^{-{\textbf{i}}\theta }+\alpha _2e^{-2{\textbf{i}}\theta }+1-\beta ^*-\lambda ^*- \alpha ^* + \lambda _1 e^{{\textbf{i}}\theta }+ \lambda _2e^{2{\textbf{i}}\theta }}{1-\beta _1e^{-{\textbf{i}}\theta }-\beta _2e^{-2{\textbf{i}}\theta }}, \quad \theta \in [-\pi ,\pi ]. \end{aligned}$$

This, as well as higher-order interaction models, possibly including “hub” regions like the thalamus that would be mutually interconnected with all layers in the hierarchy (Hwang et al. 2017), are promising directions for follow-up studies.

### Conclusion

The mathematical framework proposed here, guided by both computational considerations and neuroscientific inspiration, can be of use to both fields. In machine learning, the framework may serve to provide guarantees about the stability of a predictive coding system given its chosen hyperparameters, or to choose a valid range for these hyperparameters. For neuroscientists, our equations can be used directly to understand biological vision and to make predictions about biological behavior in various situations compatible with predictive coding. But this general mathematical framework (a number of hierarchically connected layers with source terms, boundary conditions, feedforward and feedback connectivity matrices, analyzed via its amplification factor function) may also be adapted to fit other models of biological perception and cognition beyond predictive coding. We hope that the various derivations made in the present work can serve as a template for future applications in this direction. And more generally, that this study may be helpful to the larger computational neuroscience community.

## Footnotes

1 Note that the notions of stability/instability and marginal stability introduced in the fully discrete setting naturally extend to the semi-continuous setting.

## References

[CR1] Alamia A, VanRullen R (2019). Alpha oscillations and traveling waves: signatures of predictive coding?. PLoS Biol.

[CR2] Alamia A, Timmermann C, Nutt DJ, VanRullen R, Carhart-Harris RL (2020). DMT alters cortical travelling waves. Elife.

[CR3] Alamia A, Terral L, d’Ambra MR, VanRullen R (2023). Distinct roles of forward and backward alpha-band waves in spatial visual attention. Elife.

[CR4] Bastos A, Usrey W, Adams R, Mangun GR, Fries P, Friston K (2012). Canonical microcircuits for predictive coding. Neuron.

[CR5] Bastos A, Vezoli J, Bosman CA, Schoffelen JM, Oostenveld R, Dowdall JR, Fries P (2015) Visual areas exert feedforward and feedback influences through distinct frequency channels. Neuron 85(2):390–401 10.1016/j.neuron.2014.12.018 25556836

[CR6] Besse C, Faye G, Roquejoffre J-M, Zhang M (2022) The logarithmic Bramson correction for Fisher-KPP equations on the lattice $${\mathbb{Z}}$$. arXiv:2206.04358

[CR7] Box GEP , Launer RL, Wilkinson GN (1979). Robustness in the strategy of scientific model building. Robustness in statistics.

[CR8] Bullier J (2001). Feedback connections and conscious vision. Trends Cogn Sci.

[CR9] Choksi B, Mozafari M, Biggs O’May C, Ador B, Alamia A, VanRullen R (2021) Predify: augmenting deep neural networks with brain-inspired predictive coding dynamics. Adv Neural Inf Process Syst 34:14069–14083

[CR10] Coeuret L (2022) Local limit theorem for complex valued sequences. arXiv:2201.01514

[CR11] Coeuret L (2023) Tamed instability for finite difference approximations of hyperbolic equations with boundary conditions. arXiv:2304.02612

[CR12] Coulombel J-F, Faye G (2022). Generalized Gaussian bounds for discrete convolution powers. Rev Mat Iberoam.

[CR13] Coulombel J-F, Faye G (2023) Sharp stability for finite difference approximations of hyperbolic equations with boundary conditions. IMA J Numer Anal 43(1):187–224

[CR14] Diaconis P, Saloff-Coste L (2014). Convolution powers of complex functions on $${\mathbb{Z} }$$. Math Nachr.

[CR15] Felleman DJDC , Van Essen (1991). Distributed hierarchical processing in the primate cerebral cortex. Cereb Cortex.

[CR16] Fries P (2015). Rhythms for cognition: communication through coherence. Neuron.

[CR17] Goldberg M, Tadmor E (1985). Convenient stability criteria for difference approximations of hyperbolic initial-boundary value problems. Math Comput.

[CR18] Goldberg M, Tadmor E (1987). Convenient stability criteria for difference approximations of hyperbolic initial-boundary value problems. II. Math Comput.

[CR19] He K, Zhang X, Ren S, Sun J (2016) Deep residual learning for image recognition. In: Proceedings of the IEEE conference on computer vision and pattern recognition, pp 770–778

[CR20] Heeger DJ (2017). Theory of cortical function. Proc Natl Acad Sci.

[CR21] Hwang K, Bertolero MA, Liu WB, D’Esposito M (2017) The human thalamus is an integrative hub for functional brain networks. J Neurosci 37(23):5594–5607 10.1523/JNEUROSCI.0067-17.2017 PMC5469300 28450543

[CR22] Knoell J, Binda P, Morrone MC, Bremmer F (2011). Spatiotemporal profile of peri-saccadic contrast sensitivity. J Vis.

[CR23] Millidge B, Seth A, Buckley CL (2021) Predictive coding: a theoretical and experimental review. arXiv preprint arXiv:2107.12979

[CR24] Pang Z (2022) Predictive coding in the brain and deep neural networks. Dissertation thesis, Université Paul Sabatier-Toulouse III

[CR25] Pang Z, Alamia A, VanRullen R (2020). Turning the stimulus on and off changes the direction of $$\alpha $$ traveling waves. Eneuro.

[CR26] Pang Z, O’May CB, Choksi B, VanRullen R (2021). Predictive coding feedback results in perceived illusory contours in a recurrent neural network. Neural Netw.

[CR27] Randles E, Saloff-Coste L (2015). On the convolution powers of complex functions on $${\mathbb{Z} }$$. J Fourier Anal Appl.

[CR28] Rao RP, Ballard DH (1999). Predictive coding in the visual cortex: a functional interpretation of some extra-classical receptive-field effects. Nat Neurosci.

[CR29] Ray S, Maunsell JH (2015). Do gamma oscillations play a role in cerebral cortex?. Trends Cogn Sci.

[CR30] Riesz F , Sz-Nagy B (1955). Functional analysis.

[CR31] Thomée V (1965). Stability of difference schemes in the maximum-norm. J Differ Equ.

[CR32] Tschantz A, Millidge B, Seth AK, Buckley CL (2022) Hybrid predictive coding: inferring, fast and slow. arXiv arXiv:2204.02169v2 10.1371/journal.pcbi.1011280 PMC10395865 37531366

[CR33] Wen H, Han K, Shi J, Zhang Y, Culurciello E, Liu Z (2018) Deep predictive coding network for object recognition. In: International conference on machine learning, pp 5266–5275

